# West Antarctic Ice Sheet advance since the early Pliocene

**DOI:** 10.1038/s41467-026-74100-1

**Published:** 2026-06-06

**Authors:** Mengwei Zhang, Julia S. Wellner, Karsten Gohl, Steven M. Bohaty, Becky A. Hopkins, Chuang Xuan, Li Wu, Anna Ruth W. Halberstadt, Theresa M. King, John M. Fegyveresi, Masako Yamane, Michelle L. Penkrot, Junling Pei, Zhenyu Yang, Yue Zhao, Denise K. Kulhanek, Adam Klaus, Liang Gao

**Affiliations:** 1 Key Laboratory of Polar Geology and Marine Mineral Resources (China University of Geosciences, Beijing), Ministry of Education, Beijing, China; 2 School of Ocean Sciences, China University of Geosciences, Beijing, China; 3 Department of Earth and Atmospheric Sciences, University of Houston, Houston, TX USA; 4 Alfred Wegener Institute Helmholtz Centre for Polar and Marine Research, Bremerhaven, Germany; 5 Institute of Earth Sciences, Heidelberg University, Heidelberg, Germany; 6 School of Ocean and Earth Science, University of Southampton, Southampton, UK; 7 Department of Earth, Ocean and Ecological Sciences, University of Liverpool, Liverpool, UK; 8 Laboratory for Coastal Ocean Variation and Disaster Prediction, College of Oceanography and Meteorology; Guangdong Ocean University, Zhanjiang, Guangdong China; 9 State Key Laboratory of Marine Geology, Tongji University, Shanghai, China; 10 Department of Earth and Planetary Sciences, Jackson School of Geosciences, The University of Texas at Austin, Austin, TX USA; 11 College of Marine Science, University of South Florida, St. Petersburg, FL USA; 12 School of Earth and Sustainability, Northern Arizona University, Flagstaff, AZ USA; 13 Institute for Space-Earth Environmental Research, Nagoya University, Furocho, Chikusa, Nagoya, Aichi Japan; 14 International Ocean Discovery Program, Texas A&M University, College Station, TX USA; 15 State Key Laboratory of Nuclear Resources and Environment, East China University of Technology, Nanchang, China; 16 College of Resources, Environment and Tourism, Capital Normal University, Beijing, China; 17 Institute of Geosciences, Christian-Albrechts-University Kiel, Kiel, Germany

**Keywords:** Cryospheric science, Palaeoclimate, Palaeomagnetism

## Abstract

Current understanding of past ice dynamics in the Amundsen Sea—the most vulnerable part of the West Antarctic ice sheet (WAIS)—remains incomplete, hampering future predictions. Here, sediments from International Ocean Discovery Program Site U1532 on the continental rise reveal that ice-sheet dynamics strongly influenced detrital magnetic minerals supply. Minimal magnetite concentration during ~4.1-3.8 Ma reflects the smallest WAIS extent in the Amundsen Sea sector since ~4.33 Ma. Since ~4.1 Ma, WAIS expansion was closely linked to long-term global cooling. After ~3.2 Ma, elevated fine-grained magnetite indicates a sustained increase in sediment contribution from the Thwaites Glacier catchment. The abrupt ice-sheet expansion at ~3.2 Ma may reflect the crossing of a climatic threshold that triggered qualitative transformations in ice-sheet development. Cessation of major WAIS growth after ~0.9 Ma aligns with stabilized long-term CO_2_ levels and ocean temperatures. Collectively, these results underscore the high sensitivity of the Amundsen Sea sector to global climate forcing.

## Abstract (web summary)

This study shows that West Antarctic Ice Sheet history in the Amundsen Sea sector since 4.33 million years ago was closely linked to global temperature change, with a major expansion threshold crossing around 3.2 million years ago.

## Introduction

**Fig. 1:**
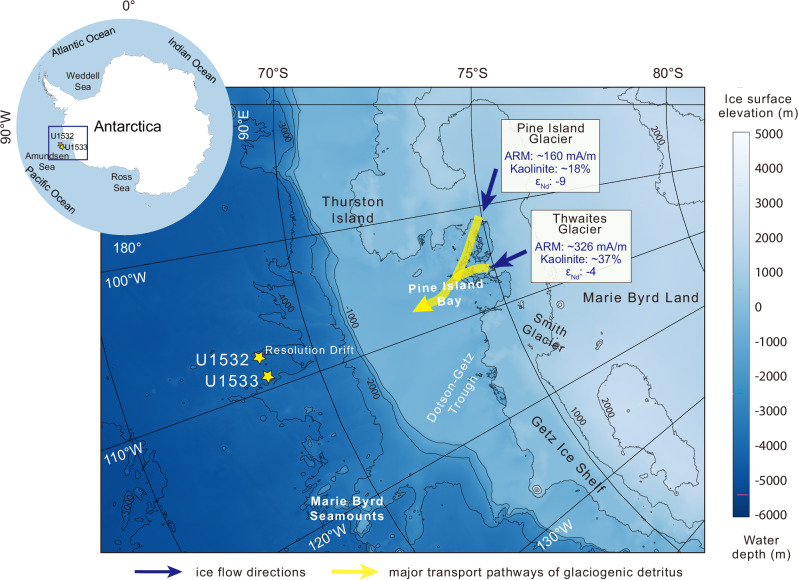
A regional map of the Amundsen Sea Embayment (ASE) and coastal Marie Byrd Land (MBL). Resolution Drift in the Amundsen Sea sector and the drill sites (U1532 and U1533, yellow stars) of the International Ocean Discovery Program (IODP) Expedition 379 are shown. Colors and contours show elevation relative to sea level derived from International Bathymetric Chart of the Southern Ocean (IBCSO) v2^83^. IBCSO v2 data are licensed under a Creative Commons Attribution 4.0 International License (https://creativecommons.org/licenses/by/4.0/). Changes were made for region cropping and color scheme. Negative contour values represent bathymetry (water depth), whereas positive values represent ice sheet surface elevation. Blue arrows indicate the ice flow directions of the major ice streams. Yellow arrows show the major transport pathways of glaciogenic detritus in Pine Island Bay^38^. The text boxes summarize the notable geochemical (detrital sediment Nd isotope compositions, ε_Nd_), clay mineralogical (kaolinite content), and rock magnetic properties (anhysteretic remanent magnetization, ARM) used to distinguish the provenance fingerprints of Pine Island Glacier and Thwaites Glacier^38^.

The West Antarctic Ice Sheet (WAIS), grounded largely below sea level, is highly sensitive to oceanic warming and represents a critical potential source of global sea-level rise^1–4^. The most dynamic sector of the WAIS is the Amundsen Sea Embayment (ASE, Fig. 1), where Thwaites and Pine Island Glaciers are undergoing rapid retreat and ice loss^4,5^. Satellite observations indicate that this region has been the largest contributor to Antarctic ice mass loss over the past several decades^6^, with its current phase of retreat having begun as early as the 1940s^7,8^. A total collapse of the ASE sector could contribute ~1.5 m to global sea levels^9^, with potential to destabilize the entire WAIS^10,11^. While Holocene sedimentary records establish the long-term sensitivity of the WAIS to warm Circumpolar Deep Water (CDW) incursions^12,13^, the recent acceleration of retreat has been directly linked to observed warming and increased access of CDW to the continental shelf^14,15^. While million-year Antarctic ice volume trends, inferred from benthic δ^18^O, are generally controlled by CO_2_ concentrations^16^, direct geological evidence remains limited. Existing Amundsen Sea records focus on the late Pleistocene and recent decades, while longer-term records from the Oligocene or Cretaceous offer glimpses of a very different, ice-free West Antarctica^17,18^. A critical knowledge gap exists between these scales, leading to significant discrepancies among ice sheet models regarding the timing of past collapses and the magnitude of future sea-level rise^1,2,19^. Consequently, obtaining direct geological evidence of the WAIS’s behavior throughout the Neogene and Quaternary is essential to reconstruct its history, understand its fundamental dynamics, and ultimately improve forecasts of its future response to climate change.

Site U1532, drilled on a sediment drift of the Amundsen Sea continental rise during International Ocean Discovery Program (IODP) Expedition 379 (Fig. 1), has provided a continuous, high-resolution sedimentary sequence extending back to ~5.7 Ma^20,21^. This exceptional archive offers a unique opportunity to investigate the long-term dynamics of the WAIS. In this study, we investigate changes in magnetic minerals to assess paleoenvironmental changes and WAIS dynamics since ~4.33 Ma. Environmental magnetism provides information on changes in the magnetic mineral grain size, mineralogical composition and concentration of magnetic particles in the sediments, and thus about provenance and environmental changes. Specifically, the anhysteretic remanent magnetization (ARM), which serves as a proxy for fine-grained magnetite concentration, is used here to reconstruct ice sheet dynamics.

## Results and discussion

**Fig. 2:**
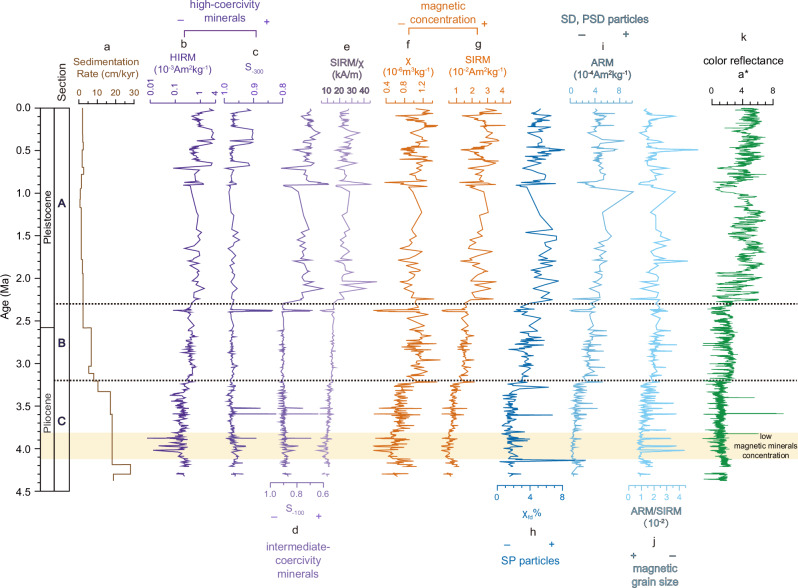
Variation of environmental magnetic parameters since ~ 4.33 Ma (the upper 300 m of the core) at Site U1532. **a** Sedimentation rates. **b** Hard isothermal remanent magnetization (HIRM). **c** S_-300_. **d** S_-100_. **e** Ratio of saturation isothermal remanent magnetization (SIRM) to mass magnetic susceptibility (χ). **f** χ. **g** SIRM. **h** Percentage frequency dependent magnetic susceptibility (χ_fd_%). **i** Anhysteretic remanent magnetization (ARM). **j** ARM/SIRM. **k** Shipboard color reflectance a*^20^. Sections A-C are separated by dashed lines: A (39.01-0 m, 2.29-0 Ma), B (88.02-39.01 m, 3.21-2.29 Ma) and C (291.43-88.02 m, 4.33-3.21 Ma). Magnetic mineralogy indicators HIRM, S_-300_, S_-100_ and SIRM/χ are plotted in purple lines; magnetic concentration indicators χ and SIRM are plotted in orange lines; magnetic grain size indicators χ_fd_%, ARM, and ARM/SIRM are plotted in blue lines. Higher HIRM and lower S_-300_ indicate more high-coercivity minerals; lower S_-100_ indicate more intermediate-coercivity minerals; variable SIRM/χ indicates changes in mineral composition; higher χ and SIRM for higher magnetic concentrations; higher χ_fd_% and ARM for more superparamagnetic (SP) and single domain (SD)/pseudo single domain (PSD) particles; higher ARM/SIRM for smaller magnetic grain size. The light-yellow shaded area (~4.12-3.82 Ma) marks a period with extremely low concentrations of magnetic minerals, indicating a period of extreme warmth. See Supplementary Table 2 for age-depth model. Source data are provided as a Source Data file.

Based on the changes of values of magnetic properties measured on cube samples, we divide the upper 291.43 m (since ~4.33 Ma) of the core into three sections: A, B, and C (Fig. 2). Variations in these properties are described from Section C to A, following the chronological sequence of the core. In Section C (291.43–88.02 m), low hard isothermal remanent magnetization (HIRM) values are observed. S_-300_ values approach 1, and S_-100_ values average 0.9. Low values are recorded for mass magnetic susceptibility (χ), saturation isothermal remanent magnetization (SIRM), percentage frequency dependent magnetic susceptibility (χ_fd_%), ARM, ARM/SIRM, and SIRM/χ (~10 kA/m). In Section B (88.02-39.01 m), HIRM values are higher than in Section C. S_-300_ and S_-100_ values show little change. SIRM/χ increases to ~14 kA/m. Higher values are recorded for χ, SIRM, χ_fd_%, ARM, and ARM/SIRM relative to Section C. In Section A (39.01-0 m), HIRM values are higher than in Section B. S_-300_ remains similar, while S_-100_ decreases noticeably. SIRM/χ rises to ~25 kA/m. χ is slightly lower than in Section B, whereas SIRM, χ_fd_%, and ARM are higher. ARM/SIRM is moderately lower than in Section B. For clarity, Supplementary Table 1 summarizes the minimum, maximum, and mean values of each magnetic property.

### Segmented variation in magnetic mineral composition, concentration, and grain size

Supplementary Table 3 provides the interpretations of magnetic parameters used in this study, and the formulas for the derived parameters are given in Methods. In Section C, the low HIRM values, combined with S_-300_ values approaching 1, indicate a very low concentration of high-coercivity magnetic minerals^22^ (Supplementary Text 1 and Supplementary Fig. 1). The low values of χ and SIRM indicate low magnetic concentration^22^. Low χ_fd_% and ARM values reveal a deficit of superparamagnetic (SP)^23^ and single domain (SD) particles^22^, while low ARM/SIRM values suggest a large magnetic grain size^22^. An interval (Fig. 2, light-yellow shaded area) exhibits notably low χ, SIRM and ARM values, indicating a significantly reduced magnetic mineral concentration in the sediments.

**Fig. 3:**
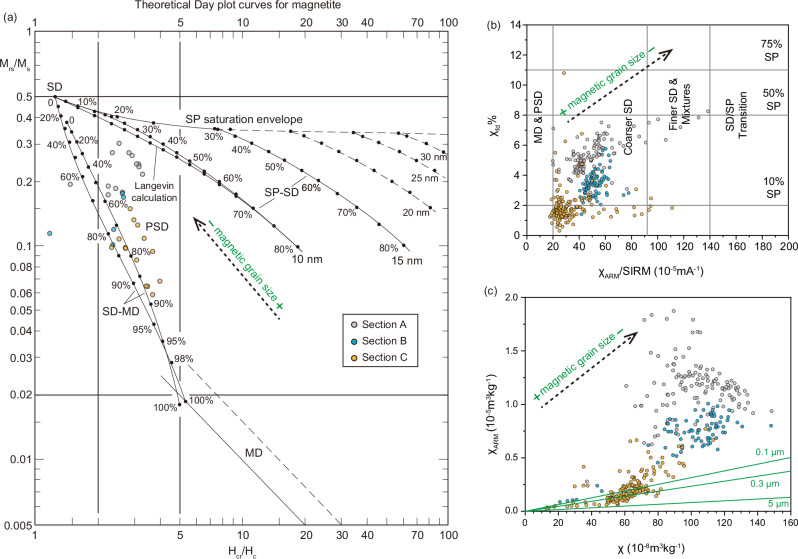
Bi-plots of magnetic parameters and inter-parametric ratios indicating the magnetic grain size at Site U1532. **a** Day plot^84^ with calculated curves for magnetite. Axis labels: M_rs_ = saturation remanent magnetization; M_s_ = saturation magnetization; H_cr_ = coercivity of remanence and H_c_ = coercivity. **b** Scatter plot of the ratio of susceptibility of anhysteretic remanent magnetization (χ_ARM_) to isothermal remanent magnetization (SIRM) vs. percentage frequency dependent magnetic susceptibility (χ_fd_%)^85,86^. **c** Bi-plot of mass magnetic susceptibility (χ) vs. χ_ARM_ (known as the King plot^87^). Green lines suggest the magnetic grain size, and points falling above the green lines represent finer magnetic mineral particles. Yellow symbol: Section C; blue symbol: Section B; gray symbol: Section A. An upcore fining trend in magnetic grain size is observed from Section C to Section A. χ_ARM_ was calculated by dividing the anhysteretic remanent magnetization (ARM) by the strength of the bias direct current field (0.05 mT, equivalent to 39.8 A/m), and it shares the same meaning as ARM. SP: superparamagnetic grains. SD: single domain. PSD: pseudo single domain. MD: multi domain. Source data are provided as a Source Data file.

Compared to Section C, sediments in Section B exhibit more high-coercivity minerals (Fig. 2b and c), a higher magnetic concentration (Fig. 2f and g), with a notably finer grain size (Fig. 2h–j and 3). The low values of the ratio SIRM/χ (~10-15 kA/m) in sections B and C are characteristic of (titano)magnetite and/or maghemite^24^. The magnetic particle sizes in samples of three sections are less than 5 μm, with most of them smaller than 0.3 μm (Fig. 3c).

In Section A, the significant decrease in S_-100_ values, without a corresponding change in S_-300_, suggests an increase in intermediate-coercivity (~100-300 mT) minerals. The presence of an additional isothermal remanent magnetization (IRM) decomposition component near ~130 mT (Supplementary Text 2 and Supplementary Figs. 2a and 3), along with χ-T curve results (Supplementary Text 3 and Supplementary Fig. 4), indicates the presence of maghemite. Thermal demagnetization identified hematite as the source of the elevated SIRM/χ values (~25 kA/m) in Section A (Supplementary Text 4 and Supplementary Fig. 5). The negative correlation between SIRM/χ and S_-100_ suggests synchronous changes in hematite and maghemite (Supplementary Fig. 6). In general, the magnetic assemblage in Section A is characterized by a high concentration and a smaller grain size (Fig. 2). The different trends in χ and SIRM should be due to changes in mineral composition. The downcore shift from more brownish to gray colors at a sub-bottom depth of ~39 m (~2.29 Ma, the boundary of sections B and A) is evident from an apparent drop in color reflectance a* values^20^ (Fig. 2k), which probably marks the Fe-redox boundary^25^. In most pelagic settings, the oxic/anoxic boundary occurs at decimeter-scale depths; however, in Antarctic drift deposits, it is typically deeper (~10 m or more) due to low labile organic matter content^26^. Therefore, sediments above the boundary exhibit a brownish color caused by elevated hematite and maghemite, consistent with observations from the JR298 core off the Antarctic Peninsula^26^, despite the notably deeper oxic/anoxic boundary (~39 m) at Site U1532. The ARM/SIRM values in Section A are not high compared to Section B because the SIRM in Section A is significantly larger due to the influence of hematite.

The Curie temperature of magnetite^22^ revealed by the χ-T curves (Supplementary Text 3 and Supplementary Fig. 4), together with the unblocking of over 98% of the soft magnetic component at ~590 °C (Supplementary Text 4 and Supplementary Fig. 5), suggests that the magnetic properties are largely dominated by magnetite. The presence of central ridges in first-order reversal curve (FORC) diagrams indicate the existence of magnetofossils (Supplementary Text 2 and Supplementary Fig. 2). However, transmission electron microscopy (TEM) observations (Supplementary Text 5 and Supplementary Fig. 7), combined with the lack of correlation between the IRM contribution of biogenic magnetite and χ, indicate that magnetofossils are much rarer compared to detrital magnetite (Supplementary Text 6, Supplementary Figs. 8 and 9). The contribution of biogenic magnetite was then disregarded when discussing the sources of magnetic minerals. Therefore, magnetic analysis reveals the presence of detrital and biogenic magnetite, detrital and authigenic hematite, and authigenic maghemite. Downcore dissolution of magnetic minerals does not have significant effect on the characterization of magnetic concentration and grain size because of the low concentration of total organic carbon, interstitial water sulfate, and the synchronous variation in the content of magnetic minerals and kaolinite (Supplementary Text 7, Supplementary Figs. 10 and 11).

### Magnetite tracks ice sheet dynamics

The magnetic minerals of the Amundsen Sea continental rise are predominantly of terrigenous origin (Supplementary Text 6). Here, terrigenous sedimentation is controlled by glacial processes that generate glaciogenic detritus on land and the continental shelf, which is then transported downslope via gravity flows—such as slumps and turbidity currents—through deep-sea channels to the continental slope and rise^27–30^. This process occurs primarily when the ice sheet advances onto the continental shelf during glacial periods^27–30^. On the continental rise, particles still in suspension were captured by eastward flowing bottom currents to form sediment drifts^27–30^. Sedimentary facies analysis of Expedition 379 cores indicates that terrigenous debris was primarily delivered to the continental rise by glacial-advance gravity flows and deglacial hyperpycnal plumes generated while the grounding line remained near the shelf break and meltwater discharge intensified^31^. In contrast, during interglacial periods, ice-sheet retreat resulted in a diminished flux of terrigenous material transported downslope, thereby promoting the enrichment of biogenic components within the drift sediments^32^. It is recognized that current systems near the core site—including the Antarctic Circumpolar Current (ACC), the Antarctic Slope Current (ASC), and bottom waters influenced by Ross Sea-derived Antarctic Bottom Water (AABW)^33^—can, in principle, influence sediment transport and deposition. However, detrital Nd-Pb isotope data from Site U1532 sediments show that throughout the Plio-Pleistocene, sediment supply variability driven by ocean currents was negligible at the core site, with downslope transport of material eroded from the hinterlands of the Pine Island and Thwaites basins being the dominant source^34^.

**Fig. 4:**
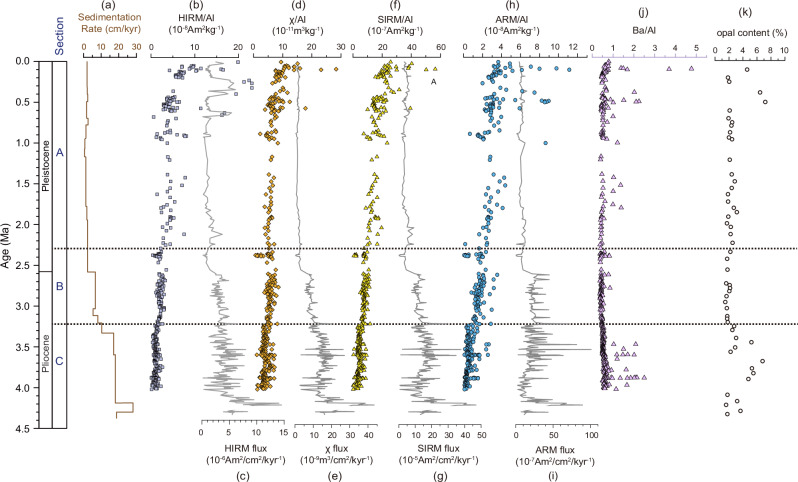
Terrigenous and biogenic input variations since ~4.33 Ma at Site U1532. **a** Sedimentation rates. **b** Hard isothermal remanent magnetization (HIRM)/Al. **c** HIRM flux. **d** Mass magnetic susceptibility (χ)/Al. **e** χ flux. **f** Saturation isothermal remanent magnetization (SIRM)/Al. **g** SIRM flux. **h** Anhysteretic remanent magnetization (ARM)/Al. **i** ARM flux. **j** Ba/Al, indicative of paleoproductivity. **k** Opal content. The flux of magnetic minerals was obtained by multiplying the environmental magnetic parameters by the sedimentation rate and dry density. HIRM flux represents the flux of high-coercivity minerals, while χ flux and SIRM flux represent the flux of total magnetic minerals. ARM flux represents the flux of fine-grained magnetite. Source data are provided as a Source Data file.

Magnetite concentration increased in Section B compared to Section C, whereas sedimentation rates decreased over the same interval (Fig. 2a)^20^. The parallel decline in magnetic mineral fluxes and sedimentation rates (Fig. 4) indicates a common glacial source and an overall reduction in terrigenous input. The decoupling between decreasing flux and increasing magnetite concentration could be explained by several mechanisms. (1) Reduced dilution by biogenic components. Although colder Pleistocene conditions could, in principle, lower diatom productivity (e.g., along the western Antarctic Peninsula margin after ~3 Ma^35,36^) and thus reduce dilution, this is unlikely to be the dominant mechanism at Site U1532. First, productivity proxy Ba/Al show no significant change across the interval (Fig. 4j). Second, our samples are primarily from glacial periods, when the Amundsen Sea continental rise is already characterized by minimal opal content and is dominated by terrigenous sedimentation^37^. From Section C to Section B, the decrease in biogenic material is far insufficient to account for the observed changes in magnetic mineral concentration (Supplementary Fig. 12). Thus, reduced biogenic dilution cannot explain the increase in magnetite concentration. (2) A change in sediment provenance. First, examination of magnetic fluxes reveals a decoupling: while χ flux, SIRM flux, and HIRM flux all decrease in parallel with sedimentation rate after ~3.2 Ma, ARM flux remains slightly elevated between ~3.2 and ~2.6 Ma compared to the preceding interval (Fig. 4). This demonstrates that the supply of SD magnetite was decoupled from bulk terrigenous input. Meanwhile, grain-size indicators confirm a fining of the magnetic mineral assemblage after ~3.2 Ma (Figs. 2j and 3). Second, normalizing concentration parameters to conservative terrigenous elements (Al, Ti) yields ratios that suggest the magnetic mineral content per unit mass of terrigenous detritus. These ratios show an increase after ~3.2 Ma, consistent with the raw magnetic data (Fig. 2, Supplementary Figs. 13 and 14). Thus, while the total terrigenous flux decreased, the terrigenous fraction itself became more magnetic-mineral-rich. Together, these observations point to a change in sediment provenance after ~3.2 Ma.

**Fig. 5:**
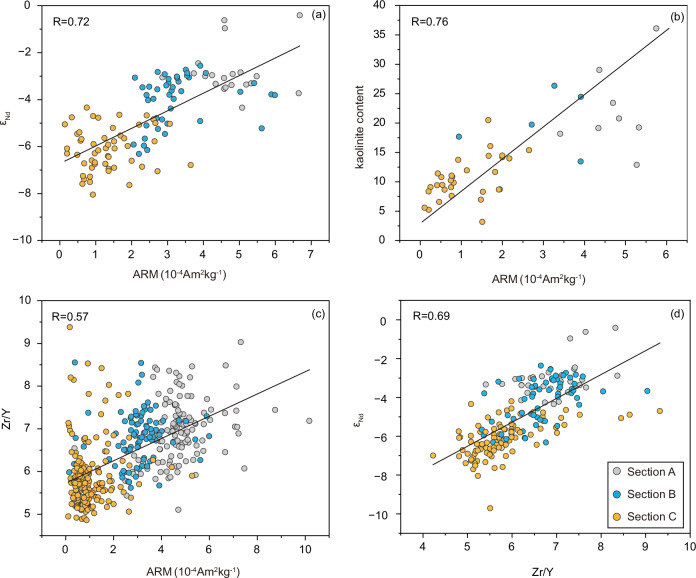
Variations in terrigenous sediment composition. **a** Biplot of anhysteretic remanent magnetization (ARM) vs. detrital sediment Nd isotope compositions (ε_Nd_). **b** ARM vs. kaolinite content^20^. **c** ARM vs. Zr/Y; **d** Zr/Y vs. ε_Nd_. R: Pearson’s correlation coefficient. Yellow symbol: section C; blue symbol: section B; gray symbol: section A. Data of ε_Nd_ is from^34,44^ (Supplementary Text 8). Source data are provided as a Source Data file.

Glaciogenic detritus delivered by Thwaites Glacier is characterized by higher detrital sediment Nd isotope compositions (ε_Nd_) values, kaolinite content, and mafic minerals compared to that of Pine Island Glacier (Fig. 1)^34,38,39^. These distinctive fingerprints allow us to trace the contribution of each source through time. Clay mineralogy has been utilized for provenance tracing of sediments in the Amundsen Sea continental rise^31,37,40–42^. The content of clay mineral kaolinite is generally associated with chemical weathering of rock in warm and humid climates^43^, and it has been suggested that pre-Oligocene sedimentary strata on the ASE shelf and its hinterland are the sources for the relatively high kaolinite contents found in the offshore sediments of the Amundsen Sea^41,42^. Both shipboard kaolinite content and ε_Nd_ show positive correlations with magnetic concentration parameters (Fig. 5 and Supplementary Fig. 15). Zr/Y ratios, which are elevated in western Amundsen Sea detritus (6.4-9.2) compared to eastern Amundsen Sea (4.4-7.7)^39^, also increase after ~3.2 Ma and correlate positively with ARM and ε_Nd_ (Fig. 5 and Supplementary Fig. 15). During the Pliocene, despite differences in isotopic signatures between glacial and interglacial periods, both glacial and interglacial sediments at Site U1532 exhibit low ε_Nd_ values (∼−7 to −4)^44^. In contrast, throughout the Pleistocene, ε_Nd_ values remain stable and high (∼−4 to −2), regardless of glacial-interglacial cycles^34^. The synchronous increase in magnetic mineral concentration and ε_Nd_ thus confirms that the post-3.2 Ma magnetite enrichment reflects a long-term change in sediment provenance.

Among all concentration-related magnetic parameters, ARM shows the strongest correlations with detrital ε_Nd_, kaolinite, and Zr/Y (Fig. 5), suggesting that SD magnetite dominates the magnetic mineral assemblage of this provenance. ARM shows consistent long-term trends with ARM/Al and ARM/Ti (Supplementary Fig. 16). Most importantly, integrated aerogeophysical surveys have revealed a zone of Cretaceous mafic magmatism extending ~200 km inland from the coast beneath the Thwaites Glacier catchment^45^. Such mafic rocks are characterized by high magnetic concentrations, providing a direct geological source for the magnetite-rich detritus at Site U1532. Meanwhile, sedimentary basins situated ~150–200 km inland of the coast, covering 20% of the TG catchment^45,46^, are a potential source of kaolinite^31,42^. Prior erosion of mafic rock bodies likely generated mafic debris, which subsequently mixed with other sediment sources, such as the adjacent sedimentary basin fill^47^. This mixed material was then transported to the sampling site by glacial processes. Although direct ε_Nd_ data from the Thwaites mafic intrusions are lacking, coeval mafic rocks (~113-98 Ma) in the Fosdick Mountains (western Marie Byrd Land) formed during the same rift event yield ε_Nd_ values of −1.6 to +2.5^48,49^, suggesting that the Thwaites intrusions likely have comparable radiogenic signatures and thus contributed to the post-3.2 Ma ε_Nd_ increase. Early Pliocene sediments at Site U1532 reveal that sedimentation on the Amundsen Sea continental rise was strongly coupled to Thwaites Glacier dynamics^31^.

The decrease in sedimentation rate at Site U1532 was explained by a change in ice-sheet configuration^34^: as the WAIS expanded to its near-modern size during the Plio-Pleistocene transition, glacial-interglacial fluctuations became confined to the marine realm, reducing erosion of continental bedrock and thus lowering total sediment supply^34^. This effect was likely amplified by progressive glacial over-deepening of the shelf, which hindered grounding-line advance to the shelf edge during subsequent glacial maxima. Over-deepening and the associated reduction in glacially influenced sedimentation are well documented on the Ross Sea shelf from the Pliocene to present^50,51^. Additionally, the volcanic rocks beneath Thwaites Glacier are inherently more resistant to glacial erosion^45^, which may have further contributed to the reduced sedimentation rate. In short, the decrease in magnetic mineral flux was caused by reduced sedimentation rate (Fig. 4), whereas the increase in magnetic mineral concentration resulted from a provenance shift.

We therefore adopt ARM as the proxy for ice-sheet dynamics: its increase after ~3.2 Ma records the progressive expansion of the WAIS into the magnetite-rich mafic province beneath Thwaites Glacier (Supplementary Fig. 17), reflecting a reorganization of ice flow and erosion patterns as the ice sheet approached its modern configuration. The strong correlations between ARM and global climate proxies (deep ocean temperature, T_w_; sea surface temperature change, ΔSST; benthic δ^18^O and sea level) thus reflect the coupling between WAIS dynamics and global environmental change (Supplementary Fig. 18).

### Mechanisms of WAIS variation and climate coupling

**Fig. 6:**
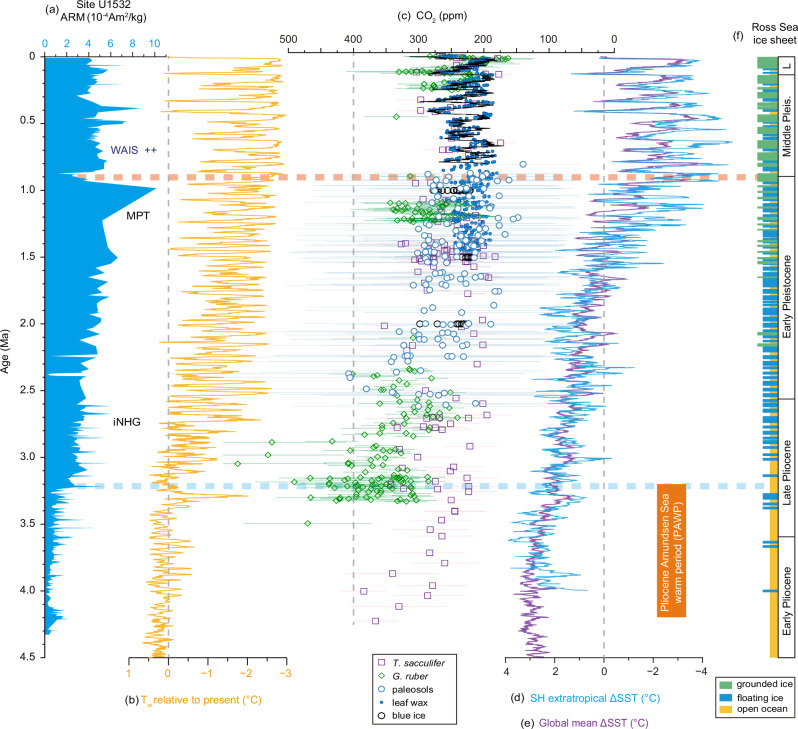
West Antarctic Ice Sheet (WAIS) evolution since ~4.33 Ma recorded by anhysteretic remanent magnetization (ARM) at Site U1532. **a** ARM values of Site U1532, used as indicator for ice sheet advance/retreat. **b** Deep ocean temperature (T_w_) relative to present (0 ka BP)^88^. **c** Proxy and ice-core CO_2_ data. Purple squares and green diamonds are harmonized boron isotopic composition (δ^11^B) values on planktonic foraminifera *T. sacculifer* and *G. ruber*, respectively^89^. Blue hollow circles are based on measurements on paleosols from the Chinese Loess Plateau^90^. Blue dots are from carbon isotopic composition (δ^13^C) of leaf wax^91^. Black lines are ice-core CO_2_ record over the past 800 ka^92^ and black hollow circles are early Pleistocene blue-ice CO_2_ snapshots^93^. **d** Southern Hemisphere (SH) extratropical sea surface temperature change (ΔSST)^52^. **e** Global mean ΔSST^52^; gray dashed line: preindustrial level^52^. **f** Modeled sediment facies in the Ross Sea, close to AND-1B (yellow = open ocean; blue = floating ice; green = grounded ice)^2^. Yellow and blue/green here correspond to the AND 1B diatomite (yellow) and diamictite (green) intervals^53^. Pliocene Amundsen Sea Warm Period (PAWP): based on the seismic correlation of sediment layers from the drill sites to the continental shelf, grounding zone wedges have been identified in the shelf sediments that predominantly formed during ice sheet retreat within an extended Pliocene warm period from 4.2-3.2 Ma^32^. MPT: Mid-Pleistocene Transition. iNHG: the intensification of the Northern Hemisphere glaciation. Blue dashed line marks a transition point to a larger WAIS at ~3.2 Ma. Orange dashed line marks a transition point to Pleistocene ice sheet at ~0.9 Ma.

Atmospheric CO_2_-driven changes in ocean temperature and circulation are proposed as key factors influencing WAIS variability^2,16,52–55^. ARM-derived WAIS variations exhibit both long-term trends and short-term fluctuations. A prolonged warm interval during ~4.2-3.2 Ma has been observed around Antarctica^32,35,53,56–58^, and simulations of grounding line dynamics in response to Pliocene warm conditions suggest that retreat was a consistent and extensive feature across major Antarctic catchments during this interval^59^. The extremely low ARM indicates that the period during ~4.1-3.8 Ma marks the minimal WAIS extent since ~4.33 Ma. Provenance data from Site U1532 indicate a more extensive WAIS during ~4.1-3.6 Ma compared to the ~4.6-4.4 Ma warm interval^31^. However, the absence of magnetic data from ~4.6-4.4 Ma precludes a direct comparison. During the extended Pliocene warm period, ARM values occasionally attained values comparable to those of the Pleistocene (Fig. 6a), indicating that the ice sheet may have temporarily advanced to a larger size during some of the cooler glacial stages.

After ~4.1 Ma, a long-term trend of ice advance persisted until the Mid-Pleistocene Transition (MPT, ~0.9 Ma; Fig. 6). The link between temperature decline and ice-sheet growth is readily comprehensible, as cooling promotes ice accumulation and stability. For WAIS dynamics, T_w_ is arguably more influential, as basal melting driven by upwelling warm water is considered a primary driver of WAIS retreat^12–15^. The interval of ~4.1–3.2 Ma witnessed only very minor ice-sheet expansion, while T_w_ showed no pronounced long-term trend. Modeling indicates that T_w_ approaching present-day levels would suffice to trigger WAIS collapse^60–62^, whereas T_w_ during ~4.5-3.2 Ma were mostly higher than today, corresponding to the phase of WAIS retreat. A major ice sheet growth transition occurred at ~3.2 Ma (Fig. 6, blue dashed line). Coinciding with extensive Ross Sea ice expansion at ~3.3 Ma^53,56^, this event provides direct evidence for continent-wide WAIS growth, which is consistent with ice-sheet models^1^. The identification of ~3.2 Ma transition for ice-sheet expansion indicates that CO_2_ and temperature declines likely crossed critical thresholds, initiating a rapid growth phase. Antarctica’s marine ice sheets are proposed to have a critical CO_2_ threshold at ~400 ppm, with growth occurring below and accelerated retreat above this level due to ocean warming^54^. CO_2_ levels have remained largely below ~400 ppm since ~3.2 Ma (Fig. 6), favoring WAIS growth. Deep-ocean cooling occurring at ~3.3 Ma may have facilitated the growth of the WAIS at ~3.2 Ma.

The expansion of the WAIS from ~3.2 to 0.9 Ma coincided with sustained declines in T_w_, ΔSST and atmospheric CO_2_ levels (Fig. 6). Cooling from declining CO_2_, reinforced by ice-albedo and height/mass-balance feedbacks, rapidly amplified ice-sheet growth during orbital periods favorable to accumulation^63–66^. The consequent expansion of the Antarctic Ice Sheet enhances the formation of salty and dense AABW and increases carbon storage capacity in the deep ocean^52,67–72^. During the Late Pliocene, a northward shift of the Southern Westerly Winds (SWW)^55^ would have displaced the zone of Ekman-driven upwelling away from the Antarctic continent, reducing the onshore transport of warm CDW. Concurrently, a reconstructed decline in ACC strength after ~3.0 Ma, attributed to a Southern Ocean reconfiguration, would have further diminished the southward advection and upwelling of CDW^2,53,55^. Additionally, the global cooling and the intensification of Northern Hemisphere Glaciation (iNHG) since the Late Pliocene weakened NADW formation^73,74^, which, in turn, could have contributed to a reduction in CDW temperature. Foraminiferal assemblages from the Ross Sea shelf reveal that the strengthening trend of the ASC after ~1.82 Ma acted as a partial barrier to the upwelling of warm water^75^. A self-reinforcing cooling cycle was established: declining CO_2_ and temperatures promoted ice-sheet growth, which in turn activated atmospheric and oceanic positive feedbacks that sustained the Plio-Pleistocene cooling trend. After ~0.9 Ma, ΔSST and atmospheric CO_2_ both exhibited a modest increase, while T_w_ ceased declining—coinciding with a slight reduction in the long-term extent of the WAIS (Fig. 6). It should be noted that we do not discuss ice sheet variations at the glacial-interglacial scale of the Pleistocene herein. Therefore, the Amundsen Sea record corroborates that the WAIS evolution since the Late Pliocene has been strongly coupled with global climate change.

In summary, our study provides a long-term continuous geological record of WAIS evolution since the early Pliocene in the Amundsen Sea sector of West Antarctica, and offers proximal geological support for modeled Antarctic ice volume variations. Magnetic data reflect the properties of subglacial bedrock and can constrain ice-sheet extent by indicating sediment provenance. However, due to limited knowledge of subglacial geology, integration with subglacial bedrock drilling studies is necessary. Limited data density restricts robust glacial-interglacial interpretation and impedes precise identification of the ice-advance transition at ~3.2 Ma. A recent study indicates that a regime shift has reduced Antarctic sea-ice extent beyond the range of natural variability observed in past centuries^76^. In some respects, this Antarctic ice loss has been more abrupt, non-linear, and potentially irreversible compared to Arctic sea-ice loss^76^. Due to the hysteresis of ice-sheet growth, even if the climate were to return to present-day conditions following warming, the ice sheet would not regrow to its current volume^61^. Future high-resolution studies of the WAIS growth boundary conditions at ~3.2 Ma could therefore provide a crucial reference for current climate mitigation targets. Given the high sensitivity of the WAIS to global climate, it remains imperative to curb anthropogenic carbon emissions to mitigate future ice loss and its consequent global impacts.

## Methods

### Drill site description

IODP Expedition 379 successfully retrieved continuous sedimentary sequences from two drill sites, U1532 and U1533, on the Amundsen Sea continental rise^21^. Drift sediments on the Antarctic margin are deposited at high rates, which makes them high-priority drill targets to obtain continuous paleo-oceanographic and paleo-ice sheet records of high temporal resolution^20^. Site U1532 was drilled on the crest of “Resolution Drift”, a sediment drift previously imaged by seismic data^20,33^. Site U1533 is located 62 km west-southwest of Site U1532 on the westernmost lower flank of the Resolution Drift^21^. Both Sites U1532 and U1533 contain continuous sedimentary sequences dating back to the late Miocene, exhibiting similar lithofacies assemblages and demonstrating high comparability in depositional characteristics and paleoenvironmental context^21^. The linear sedimentation rate at this site is significantly higher than that at Site U1533, resulting in a more detailed sedimentary record. Therefore, we chose Site U1532 over Site U1533, which was drilled on the flank of Resolution Drift and spans nearly the same time, to reconstruct past WAIS dynamics. Site U1532 is located at ~270 km north of the ASE shelf edge (3961 m water depth, 68°35.7′S, 107°31.5′W; Fig. 1). The site reached a total depth of 794 m below the sea floor and recovered sediments with a maximum age of ~5.7 Ma^20,32^. Sediments retrieved at Site U1532 are mostly of terrigenous origin, with occasional occurrences of pelagic or hemipelagic components, including microfossils^20,32^. Lithofacies cyclicity was observed along the U1532 core^21^. Thin greenish (in the uppermost part of the site brownish) muddy units were characterized by the presence of siliceous microfossils, bioturbation, and ice-rafted debris (IRD)^21^. These greenish units have been interpreted to represent interglacial deposits^21^. They alternate with thick, gray laminated, predominantly terrigenous silty-clay units interpreted to represent glacial periods^21^. Age constraints used in this study were taken from the shipboard age-depth models^21^ and other nine tie points identified by Rahaman et al.^34^ (Supplementary Table 2).

### Rock magnetism

We collected 414 discrete cube samples from the upper 291.43 m (spanning the last ~4.3 Ma) of Holes U1532A-U1532C for rock magnetic investigations. The magnetic susceptibility (χ_lf_, χ_hf_) was measured at low frequency (976 Hz) and high frequency (15616 Hz) with a MFK1-FA Kappabridge. All samples then were subjected to three-axis demagnetization applying a 140 mT alternating magnetic field. An ARM was imparted to samples using a 0.05 mT direct current (DC) bias field superimposed on a decaying 100 mT alternating magnetic field, and measured with JR-6A Dual-Speed Spinner Magnetometer. After that, an IRM was sequentially imparted to the z-axis of the samples at 2.4 T, −100 mT, −300 mT, using IM10-30 Pulse magnetizer, and measured with JR-6A, respectively. The magnetization intensity after 2.4 T is taken as the SIRM. Experiments above were conducted at the Paleomagnetism and Environmental Magnetism Laboratory (PMEML) of the China University of Geosciences, Beijing (CUGB).

The L-ratio^77^ is defined as

1 $$L-{ratio}=\frac{{SIRM}-{{IRM}}_{-300}}{{SIRM}-{{IRM}}_{-100}},$$

the χ_fd_%^23^ is defined as

2 $${{{\rm{\chi }}}}_{{fd}}\%=100\times \left[\frac{\left({{{\rm{\chi }}}}_{{lf}}-{{{\rm{\chi }}}}_{{hf}}\right)}{{{{\rm{\chi }}}}_{{lf}}}\right]\%,$$

the S_-300 (−100)_ (ref. ^78^) is defined as

3 $${S}_{-300\,(-100)}=\left[\frac{-{{IRM}}_{-0.3T\,(-0.1T)}}{{{SIRM}}_{2.4T}}+1\right]\times \frac{1}{2},$$

and the HIRM^22^ is defined as

4 $${HIRM}=\frac{{{SIRM}}_{2.4T}+{{IRM}}_{-0.3T}}{2}.$$

Nine representative samples were taken at depths of 3.11, 11.09, 24.60, 34.00, 53.03, 73.01, 116.00, 172.93 and 259.03 m, for the following experiments. Hysteresis loops were measured over the range of -1 T and +1 T with a field increment of 10 mT and 1 s averaging time. IRM acquisition curves were measured in 70 logarithmic steps with a maximum field of 1 T and decomposed into a set of lognormal components following Maxbauer et al.^79^. FORC diagrams were obtained with a 1 T saturation field, B_u_ = [−40 mT, 40 mT], B_c_ = [0, 80 mT], with 130 FORCs measured and 1 s averaging time. Experiments above were conducted with the Lake Shore 8600 vibrating sample magnetometer (VSM) at the Paleomagnetism Laboratory of Institute of Geomechanics, Chinese Academy of Geological Sciences. FORC diagrams were produced by Igor Pro 9 FORCinel version 3.08 (ref. ^80^). Seventeen additional samples through the core were tested for IRM acquisition curves.

For two samples (3.12, 34.00 m), high-temperature-dependence of magnetic susceptibility (χ-T) curves were measured using a KLY-4S Kappabridge in an argon atmosphere at the PMEML of CUGB. The sample holder and thermocouple contributions to magnetic susceptibility were subtracted.

Six representative samples were taken at depths of 0.55, 6.47, 10.39, 30.50, and 164.99 m, for the high-temperature three-axis IRM Lowrie test (ref). First, using an IM10-30 Pulse magnetizer, a 2.4 T, 0.4 T, and 0.12 T forward DC pulse field were separately applied along the z-, x-, y-axis in sequence. Then, the remanent magnetization of the samples was measured inside a magnetic shielded room, followed by stepwise thermal demagnetization, with remanence measured after each step. The stepwise thermal demagnetization temperatures set in this experiment were set as follows: 26 (room temperature), 50, 100, 150, 200, 250, 300, 350, 400, 450, 500, 530, 560, 590, 620, 640, 660, 680 and 690°C.

### XRF core scanning

XRF scanning yields elemental counts, which are used to track relative down-core elemental fluctuations^81^. The measurements were taken at the IODP Gulf Coast Repository in College Station, Texas, USA, with a fourth- and one third-generation Avaatech XRF core scanner. Detailed technical specifications of the instrument can be found at http://www.avaatech.com/. The archive half core sections were scanned at every 2 cm intervals from 0 to 258.3 meters. For this study, barium (Ba) was measured at 50 kV; zirconium (Zr) and yttrium (Y) are were measured at 30 kV; titanium (Ti) and aluminum (Al) are were measured at 10 kV.

### TEM observations

Two samples, one from Section A (24.60 m) and one from Section B (53.03 m), were subjected to magnetic extraction for TEM observations. Sediments were dispersed in a glass beaker containing ~300 ml of distilled water and placed in an ultrasonic bath and vibrated for ~15 min. A plastic bag containing a rare earth magnet was then immersed into the solution and stirred for 3 minutes to collect the magnetic minerals. This step was repeated several times until the magnetic minerals were almost completely extracted. The extract was then washed with distilled water into a 5-ml centrifuge tube. A rare earth magnet was placed outside the wall near the bottom of the tube. After two hours of settling, the non-magnetic particles at the bottom of the tube were removed using a pipette. This process was repeated 2-3 times to increase the concentration of magnetic minerals in the final extracts. After that, magnetic extracts containing a little water were transferred to a small container. A TEM grid was carefully placed on the surface of the solution with the carbon film side facing down, and a rare earth magnet was suspended 1 cm above the grid to attract magnetic minerals for 5 min. TEM observations were conducted at the Electron Microscopy Laboratory, Institute of Geology and Geophysics, Chinese Academy of Sciences (EML, IGGCAS) in Beijing, using a JEOL 2100 TEM. The techniques employed include bright-field (BF) imaging, selected-area electron diffraction (SAED), energy dispersive X-ray spectra (EDS), and high-resolution transmission electron microscopy (HRTEM) imaging.

### Opal content

Biogenic silica (opal) content was measured on 53 samples selected at roughly uniform age intervals, using molybdate-blue spectrophotometer method^82^. The analyses were performed at the Marine Geochemistry Laboratory, College of Ocean and Meteorology, Guangdong Ocean University.

## Supplementary information

Supplementary Information

## Source data

Transparent Peer Review file

Source Data

## Data Availability

Shipboard data and XRF data used in this study can be found at the International Ocean Discovery Program http://publications.iodp.org/proceedings/379/datasets.html. The magnetic data of discrete samples of Site U1532 generated in this study have been deposited in the Figshare repository under 10.6084/m9.figshare.30304840. These data are also provided in the Source Data file. Source data are provided with this paper.

## Code availability

All software packages utilized in this study are publicly accessible, and no original code is reported in this study. IRM decomposition was carried out on https://maxunmix.shinyapps.io/MAX_UnMix_final_version/ following Maxbauer et al.^79^ FORC diagrams were produced by Igor Pro 9 FORCinel version 3.08 (ref. ^80^), available at this link: https://nanopaleomag.esc.cam.ac.uk/nanopaleomag-home/forcinel/download/
