# Supplementary material for: West Antarctic Ice Sheet advance since the early Pliocene
Source: Nat Commun. 2026 Jun 6;17:7240. doi: 10.1038/s41467-026-74100-1 (PMC13396233; doi:10.1038/s41467-026-74100-1)
Supplement: Supplementary file 1 — Supplementary Information [file 41467_2026_74100_MOESM1_ESM.pdf]

Supplementary Material

West Antarctic Ice Sheet advance since the early Pliocene

Zhang et al.
Corresponding author: Liang Gao; email: lgao@live.cn; gaoliang@cugb.edu.cn

This document contains additional tables, figures and texts for "West Antarctic Ice Sheet advance since the early Pliocene". The following items are included:

- Supplementary Table 1. Minimum, maximum and mean values of environmental magnetic parameters of sections A, B and C.
- Supplementary Table 2. Age constraints for Site U1532.
- Supplementary Table 3. Magnetic parameters and their interpretation.
- Supplementary Figure 1. Determination of dominant control on HIRM and S_{-ratio}.
- Supplementary Figure 2. IRM decomposition, magnetic hysteresis, and FORC analyses of representative samples.
- Supplementary Figure 3. IRM decomposition of samples from sections A-C.
- Supplementary Figure 4. Temperature-dependence of magnetic susceptibility of selected samples in section A.
- Supplementary Figure 5. Magnetic hysteresis, IRM acquisition, and Lowrie's three-axis thermal demagnetization of representative samples.
- Supplementary Figure 6. Bi-plots of S_{-100} vs. SIRM/χ for samples of section A.
- Supplementary Figure 7. TEM analyses of extracted magnetic minerals from samples at 24.60 m and 53.03 m depth.
- Supplementary Figure 8. Correlation of detrital magnetic minerals input and magnetofossils with total magnetic mineral concentration.
- Supplementary Figure 9. Bi-logarithmic plot of χ_{ARM}/χ_{lf} vs. χ_{ARM}/χ_{fd}% for Site U1532 sediments.
- Supplementary Figure 10. Downcore variation of magnetic susceptibility, kaolinite content, total organic carbon, interstitial water sulfate, Mn and Fe concentrations at Site U1532.
- Supplementary Figure 11. Downcore variation of environmental magnetic parameters at 3550-4000 ka (137.97-219.55 m of the core) at Site U1532.
- Supplementary Figure 12. Calculation of influence on ARM by biogenic dilution effect.
- Supplementary Figure 13. Normalized concentration parameters to conservative terrigenous elements Al and Ti.
- Supplementary Figure 14. Correlation of normalized concentration parameters with conservative terrigenous elements Al and Ti.
- Supplementary Figure 15. Correlation of magnetic concentration-related parameters with detrital ε_{Nd}, kaolinite content and Zr/Y.
- Supplementary Figure 16. Consistent long-term trends between ARM and terrigenous-normalized magnetic parameters (ARM/Al, ARM/Ti).

- Supplementary Figure 17. Schematic diagram of the change in ice-sheet growth and erosion patterns from the Pliocene to the Pleistocene.
- Supplementary Figure 18. Correlation of ARM with global climate proxies.
- Supplementary Text 1. Determination of dominant control on HIRM and S_{-300}.
- Supplementary Text 2. Results of IRM acquisition curves, hysteresis and FORCs.
- Supplementary Text 3. Results of high-temperature-dependence of magnetic susceptibility curves.
- Supplementary Text 4. Results of Lowrie's three-axis thermal demagnetization.
- Supplementary Text 5. TEM observations results.
- Supplementary Text 6. The source of magnetic minerals at Site U1532 and the evidence for negligible proportion of magnetofossils.
- Supplementary Text 7. Does the downcore dissolution of magnetic minerals affect the characterization of magnetic concentration and grain size based on magnetic parameters?
- Supplementary Text 8. Age control for correlation between magnetic and detrital ε_{Nd} data.

Supplementary Table 1: Minimum, maximum and mean values of environmental magnetic parameters of sections A, B and C. Changes in the values of HIRM, S_{-100} and SIRM/χ in Section A indicate change of magnetic mineral composition. Section B has higher HIRM, SIRM/χ, χ, SIRM, χ_{fd} %, ARM and ARM/SIRM than section C.

		Section A 39.01-0 m 2.29-0 Ma			Section B 88.02-39.01 m 3.21-2.29 Ma			Section C 291.43-88.02 m 4.33-3.21 Ma		
parameter	unit	Min	Max	Mean	Min	Max	Mean	Min	Max	Mean
HIRM	$10^{-3}Am^2kg^{-1}$	0.08	3.57	0.90	0.08	0.66	0.39	0.01	0.48	0.19
S_{-300}		0.90	1.00	0.97	0.83	0.99	0.97	0.82	1.00	0.97
S_{-100}		0.56	0.92	0.73	0.68	0.94	0.90	0.64	0.95	0.89
SIRM/χ	kA/m	14.76	50.71	24.76	3.69	16.76	13.51	1.82	14.38	10.22
χ	$10^{-6}m^3kg^{-1}$	0.37	1.51	1.04	0.13	1.57	1.01	0.13	1.50	0.66
SIRM	$10^{-2}Am^2kg^{-1}$	1.20	4.14	2.54	0.05	2.12	1.41	0.02	2.15	0.70
χ_{fd} %	%	2.30	8.25	5.23	1.41	5.76	3.47	0.31	10.79	1.92
ARM	$10^{-4}Am^2kg^{-1}$	0.37	10.17	4.74	0.14	5.99	3.01	0.11	5.27	1.00
ARM/SIRM	10^{-2}	0.11	5.51	1.98	1.61	3.80	2.14	0.70	4.42	1.40

Supplementary Table 2: Age constraints for Site U1532. P1-P11 are shipboard key paleomagnetic age data inferred from the correlation of magnetic polarity at Site U1532[1]. T1-T9[2] were established based on the correlation between geochemical parameters and global benthic $\delta^{18}O$ curve[3].

Tie points ID	Magneto stratigraphic tie points	Midpoint depth CSF-A(m)	Age (Ma)	Sedimentation rates (cm/ka)
**T1**		2.022	0.123	
**T2**		6.03	0.324	2.0
**T3**		7.667	0.402	2.1
**T4**		9.834	0.491	2.4
**T5**		13.424	0.696	1.8
**T6**		14.905	0.778	1.8
**P1**	C1n base	15.64	0.781	24.5
**T7**		18.099	0.95	1.5
**T8**		19.079	1.07	0.8
**T9**		19.679	1.17	0.6
**P2**	C2n top	27.6	1.778	1.3
**P3**	C2n base	30.74	1.945	1.9
**P4**	C2An.1n top	45.41	2.581	2.3
**P5**	C2An.1n base	75.71	3.032	6.7
**P6**	C2An.2n top	80.24	3.116	5.4
**P7**	C2An.2n base	87.57	3.207	8.1
**P8**	C2An.3n top	100.35	3.33	10.4
**P9**	C2An.3n base	146.42	3.596	17.3
**P10**	C3An.1n top	253.58	4.187	18.1
**P11**	C3An.1n base	285.36	4.300	28.1

Supplementary Table 3: Magnetic parameters and their interpretation.

Parameter	Interpretation
**Hard isothermal remanent magnetization (HIRM)**	Absolute concentration of high-coercivity antiferromagnetic minerals (hematite and goethite)[4].
**S_{-ratio} (S_{-300} and S_{-100})**	Relative concentrations of high-coercivity antiferromagnetic minerals (hematite and goethite) compared to low-coercivity ferrimagnetic minerals (e.g., magnetite) with values close to 1 indicating lower coercivity[5]. S_{-100} can be used to indicate the relative content of intermediate-coercivity minerals.
**L-ratio**	Validator for the explanations of HIRM and S_{-ratio}[6]. Relatively stable L-ratio values allow for original interpretations of HIRM and S_{-ratio}[6]. In contrast, variable L-ratio values can reflect alterations in the stoichiometry, density, and/or grain size distribution of the high-coercivity magnetic fraction, thus indicate changes in the provenance of high-coercivity magnetic minerals[6].
**SIRM/χ**	Indicator for magnetic mineralogy: very low values (~10 kA/m) of the ratio are characteristic of (titano)magnetite and maghemite, while hematite, goethite and pyrrhotite exhibits relatively higher values[7]. Besides, the magnetic mineral grain size has influence on this parameter[7].
**Volumetric magnetic susceptibility (κ) and mass magnetic susceptibility (χ)**	Overall concentration of magnetizable minerals[4]. Of the three magnetic parameters: susceptibility, SIRM and ARM, susceptibility shows the least dependence on magnetic mineral grain size[7].
**Saturation isothermal remanent magnetization (SIRM)**	Overall concentration of magnetic minerals, dominated by ferrimagnetic material if present[4]. It is grain-size dependent being particularly sensitive to magnetite grains smaller than a few tens of microns[8].
**Percentage frequency dependent magnetic susceptibility (χ_{fd} %)**	Relative concentration of superparamagnetic (SP, < 20 nm) grains, values of < 2% indicate the absence of SP grains whereas values of 2-10% suggest an admixture of SP and coarser magnetic grains[9].
**Anhysteretic remanent magnetization (ARM)**	Concentration of fine-grained (submicron to few microns) magnetite grains, being particularly sensitive to single domain (SD, 20-100 nm) and pseudo-single domain (PSD) particles[10,11].
**ARM/SIRM**	Magnetic grain size indicator (larger values = smaller grains), preferentially in the submicron to 10 μm range[4].

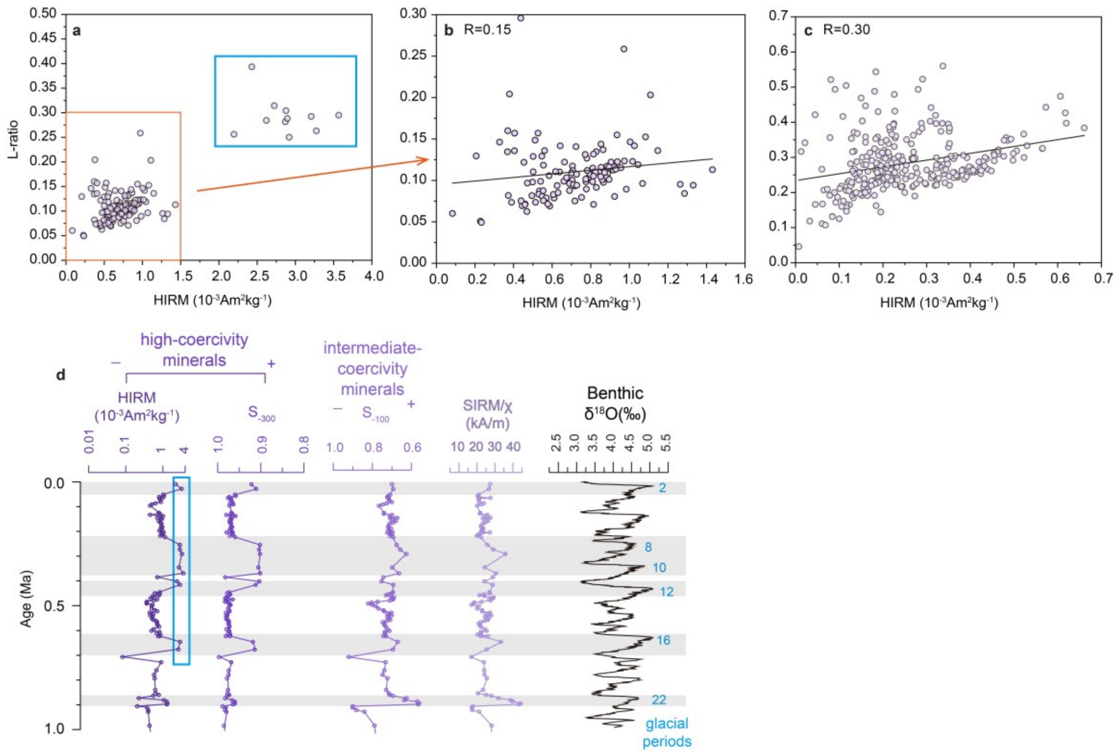

Supplementary Figure 1. Determination of dominant control on HIRM and S_{-300}. (a) hard isothermal remanent magnetization (HIRM) vs. L-ratio of samples in section A; (b) HIRM vs. L-ratio of samples in section A without high values in blue box; (c) HIRM vs. L-ratio of samples in sections B and C; (d) Downcore variation of environmental magnetic parameters since 1 Ma at Site U1532. Magnetic mineralogy indicators HIRM, S_{-300}, S_{-100} and ratio of saturation isothermal remanent magnetization (SIRM) and mass magnetic susceptibility (χ) are plotted in purple lines, respectively; global benthic $\delta^{18}O$ curve[3], with grey bars marking several glacial periods. The blue boxes in (b) and (c) enclose identical data points of high HIRM values. R: Pearson's correlation coefficient.

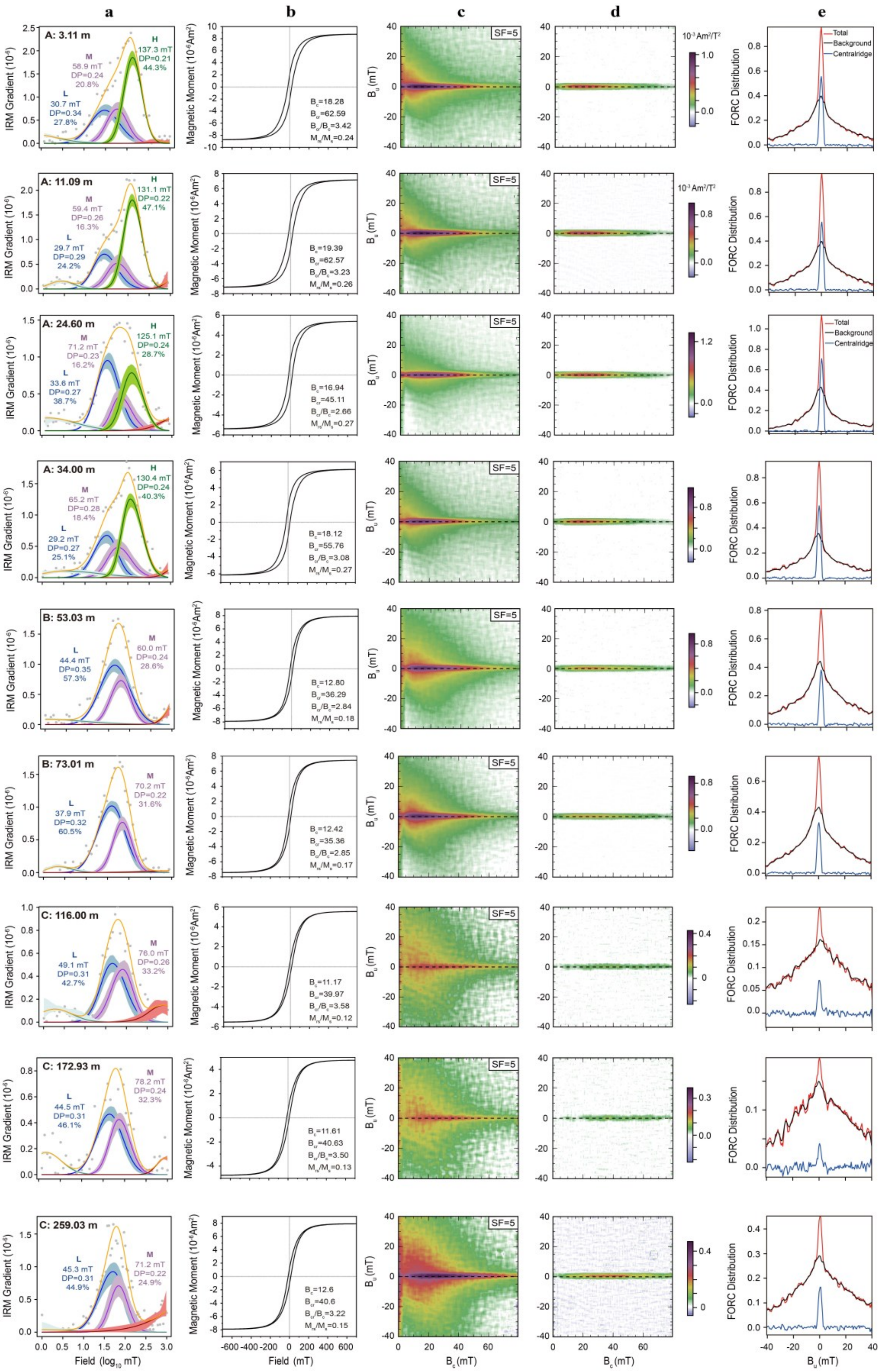

Supplementary Figure 2. IRM decomposition, magnetic hysteresis, and FORC analyses of representative samples. (a) isothermal remanent magnetization (IRM) coercivity components

decomposition using MAX UnMix[12]. Gray solid circles represent measured data, and the orange line is the fitted total coercivity spectrum. In samples from section A, five fitted components, including a very-low-coercivity component, detrital magnetite, biogenic magnetite, maghemite and a very-high-coercivity component, are reflected by cyan, blue (component L), purple (component M), green (component H) and red lines, respectively. In samples from sections B and C, four components, including a very-low-coercivity component, detrital magnetite, biogenic magnetite and a very-high-coercivity component, are reflected by cyan, blue (component L), purple (component M) and red lines, respectively. The shaded area corresponds to the 95% confidence interval on each component; (b) Hysteresis loops, including ratios of saturation remanent magnetization to saturation magnetization (M_{rs}/M_s), coercivity of remanence (B_{cr}), and coercivity (B_c); (c) FORC diagrams, with a smoothing factor (SF) of 5; (d) the central ridge component extracted from the respective FORC diagrams[13]; (e) profiles of magnetostatic-interaction field (B_u) distributions at a coercivity (B_c) of 20 mT: the central-ridge component (blue), the background component with vertical spread (black), and the total of the two (red).

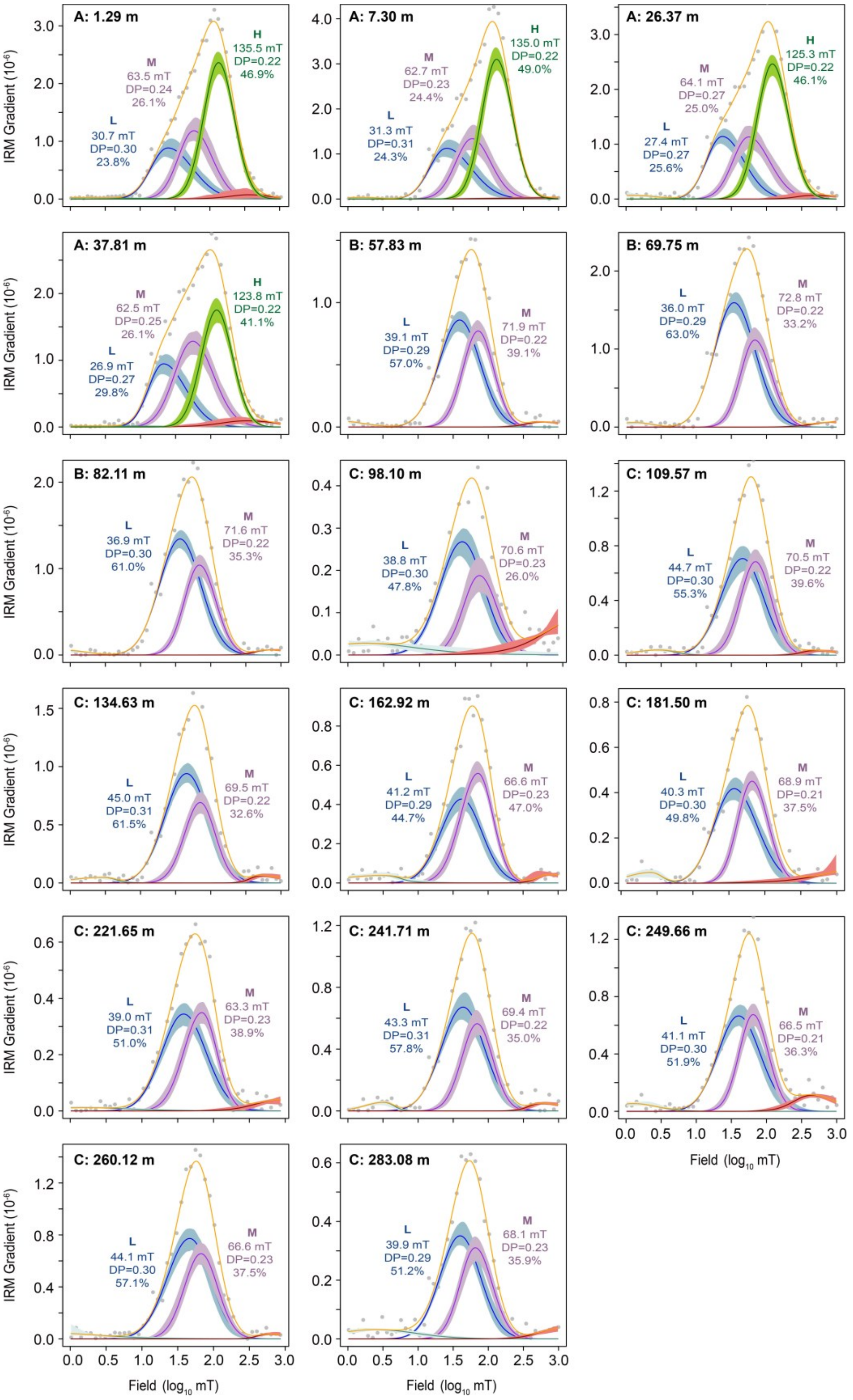

Supplementary Figure 3. IRM decomposition of samples from sections A-C. See text and caption of Figure S2 for details. IRM: isothermal remanent magnetization.

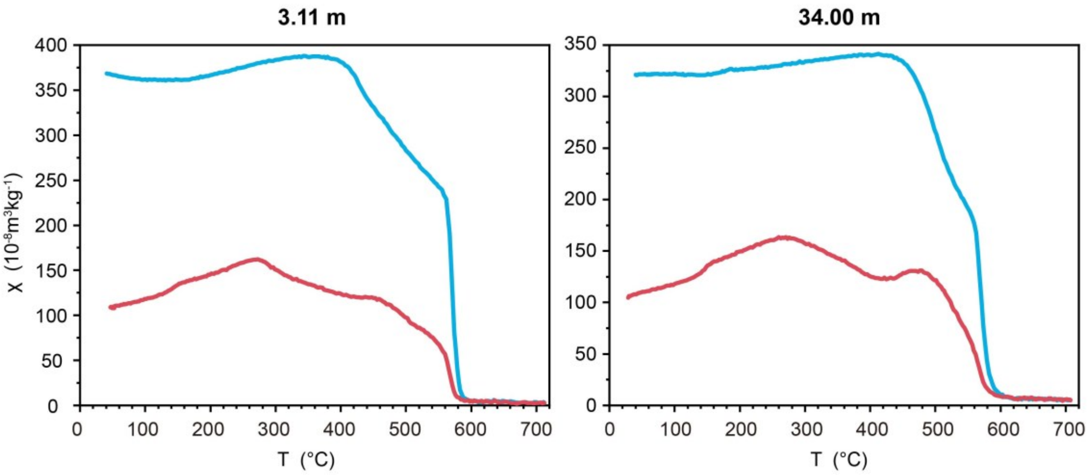

Supplementary Figure 4. Temperature-dependence of magnetic susceptibility of selected samples in section A. Red lines are heating curves and blue lines are cooling curves.

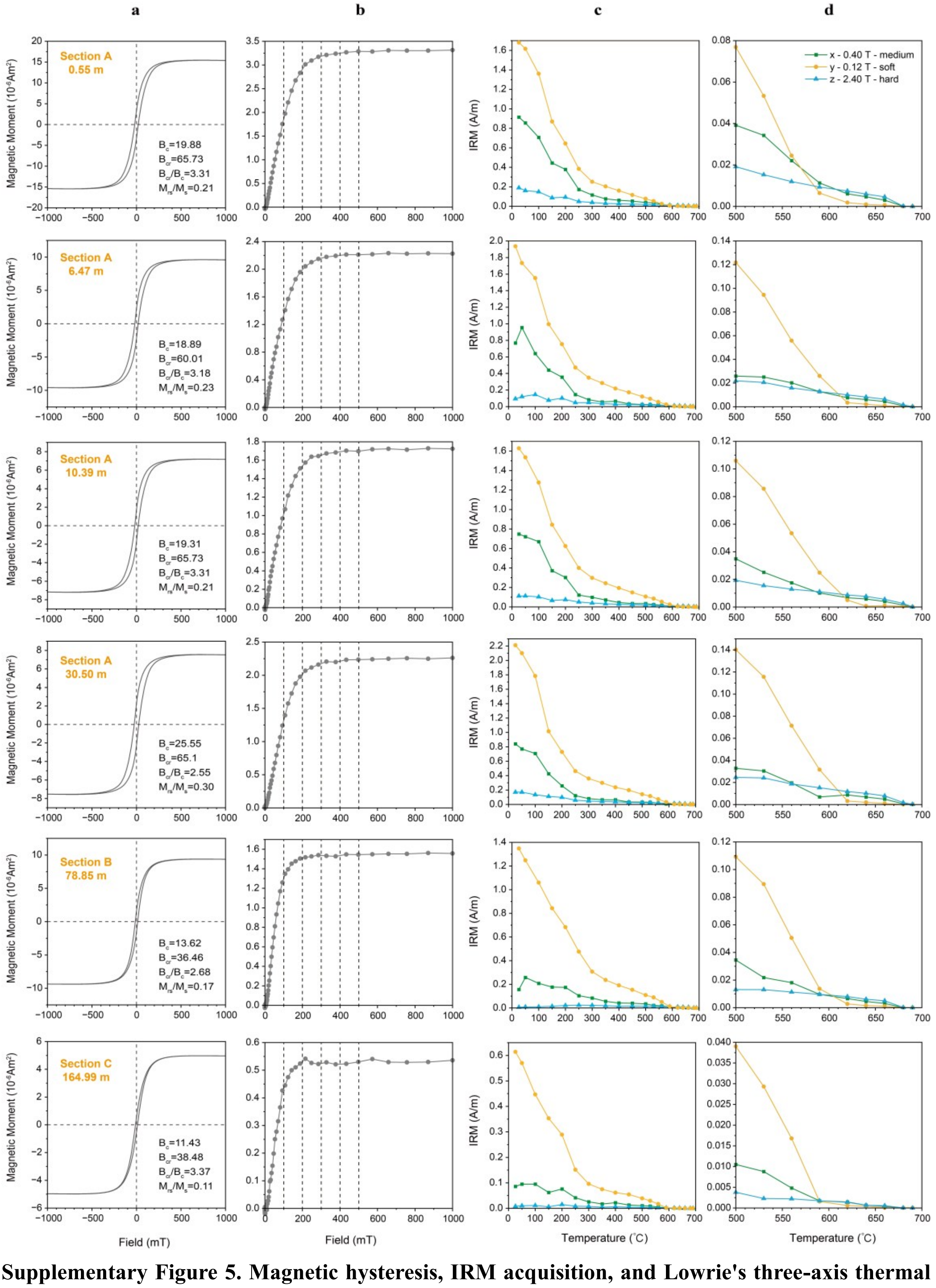

Supplementary Figure 5. Magnetic hysteresis, IRM acquisition, and Lowrie's three-axis thermal demagnetization of representative samples. (a) Hysteresis loops, including ratios of saturation remanent magnetization to saturation magnetization (M_{rs}/M_s), coercivity of remanence (B_{cr}), and coercivity (B_c); (b) Isothermal remanent magnetization (IRM) acquisition curves; (c) Lowrie's three-axis thermal demagnetization curves[14]; (d) Localized enlargement of Lowrie's three-axis thermal demagnetization curves between 500-700°C.

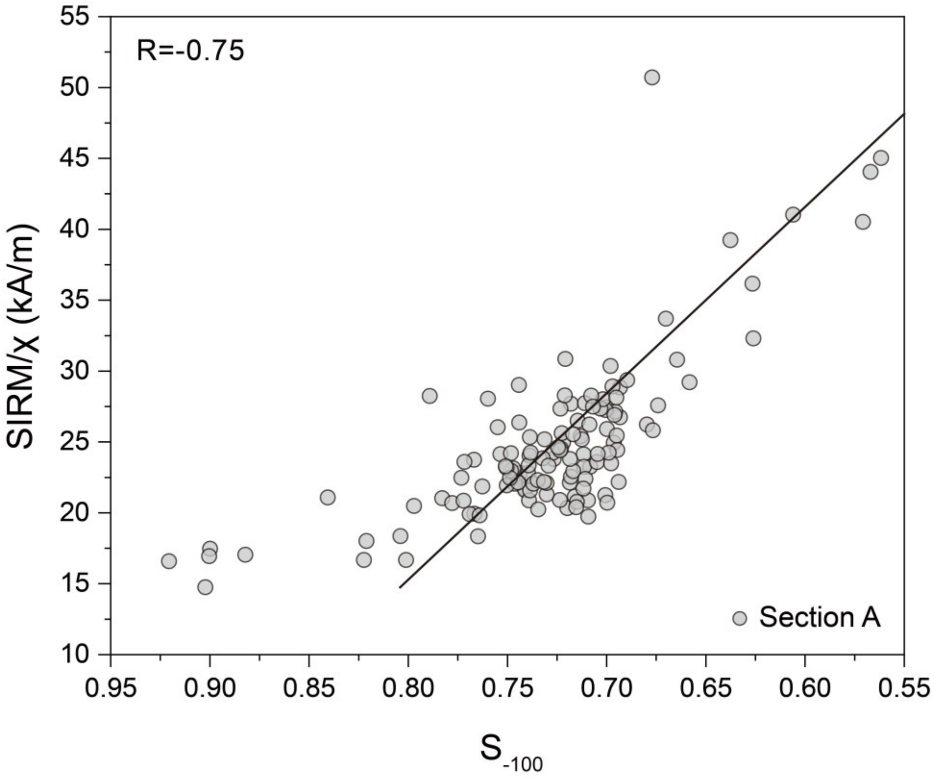

Supplementary Figure 6. Bi-plots of S_{-100} vs. SIRM/χ for samples of section A. R: Pearson's correlation coefficient. SIRM: saturation isothermal remanent magnetization; χ: mass magnetic susceptibility.

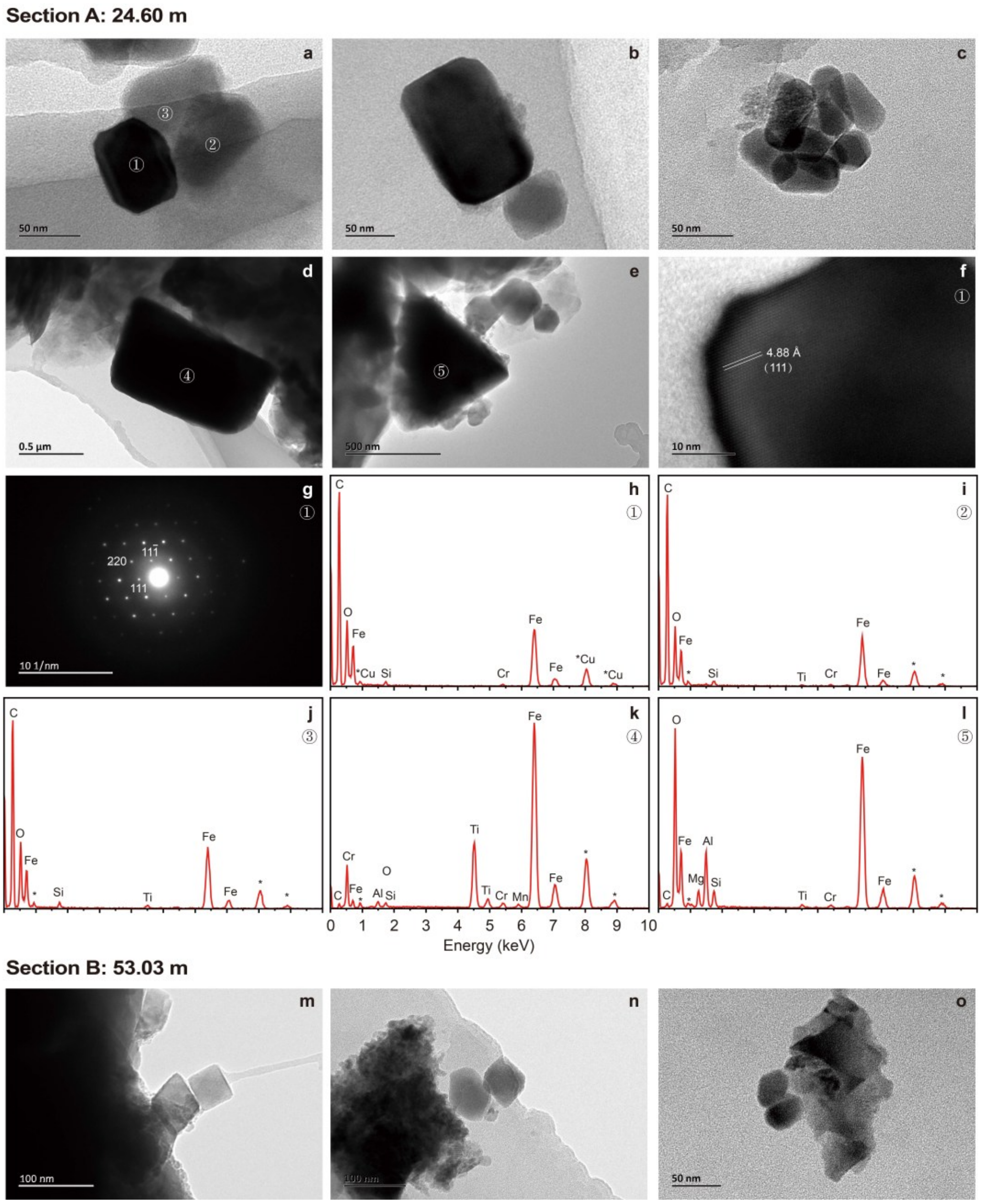

Supplementary Figure 7. TEM analyses of extracted magnetic minerals from samples at 24.60 m and 53.03 m depth. (a-c) Low-resolution bright-field TEM images of biogenic magnetite from 24.60 m sample; (d, e) Low-resolution bright-field TEM images of detrital magnetite from 24.60 m sample; (f) High-resolution bright-field TEM image of particle ①, the lattice fringes of 4.88 Å correspond to the {111} lattice plane of magnetite, indicating this particle is magnetite; (g) Selected-area electron diffraction (SAED) pattern of particle ①; (h-l) Energy dispersive spectra (EDS) of particles ①-⑤, Cu peaks originate from TEM grid, and C is from carbon film. Main elements Fe and O are indicative of magnetite; (m-o) Images of biogenic magnetite from 53.03 m sample.

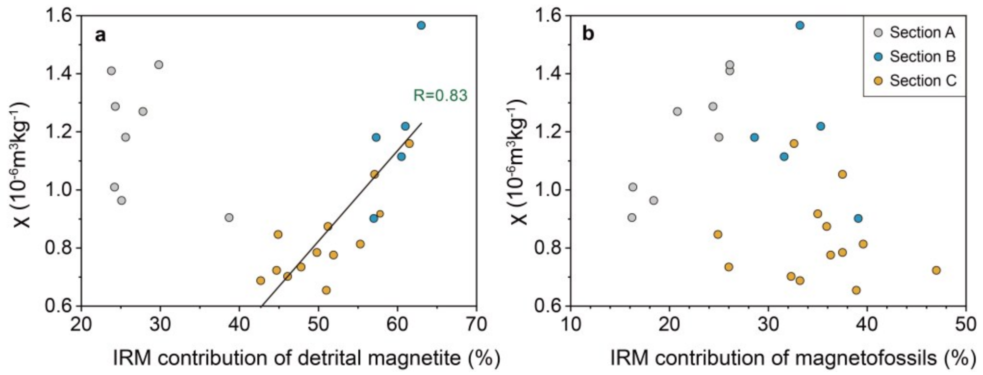

Supplementary Figure 8. Correlation of detrital magnetic minerals input and magnetofossils with total magnetic mineral concentration. (a) biplot of isothermal remanent magnetization (IRM) contribution of detrital magnetite vs. mass magnetic susceptibility (χ); (b) IRM contribution of magnetofossils vs. χ. R: Pearson's correlation coefficient. The points of samples in section A did not participate in the correlation calculation in this figure, because section A was affected by oxidation. Samples in section A had an additional main component H in the IRM decomposition.

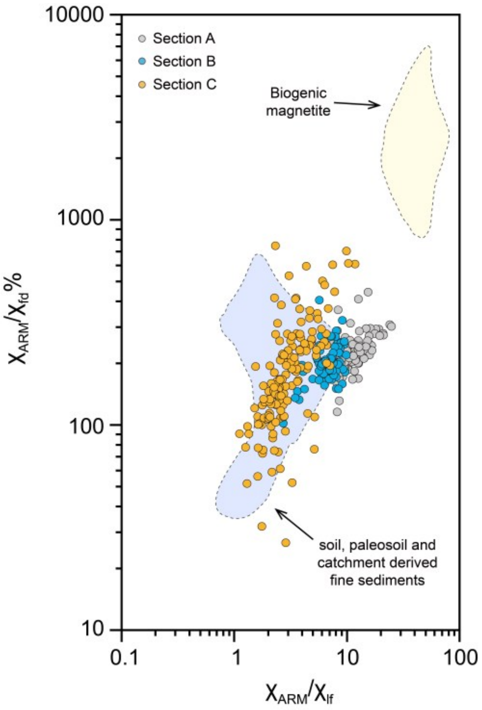

Supplementary Figure 9. Bi-logarithmic plot[15] of χ_{ARM}/χ_{lf} vs. χ_{ARM}/χ_{fd}% for Site U1532 sediments. The susceptibility of anhysteretic remanent magnetization (χ_{ARM}) was calculated by dividing the anhysteretic remanent magnetization (ARM) by the strength of the bias direct current field (0.05 mT = 39.8 A/m). Low-frequency mass magnetic susceptibility (χ_{lf}) is the same parameter as mass magnetic susceptibility (χ) in the main text. Frequency dependent magnetic susceptibility (χ_{fd}) was calculated as the difference between χ_{lf} and high-frequency mass magnetic susceptibility (χ_{hf}).

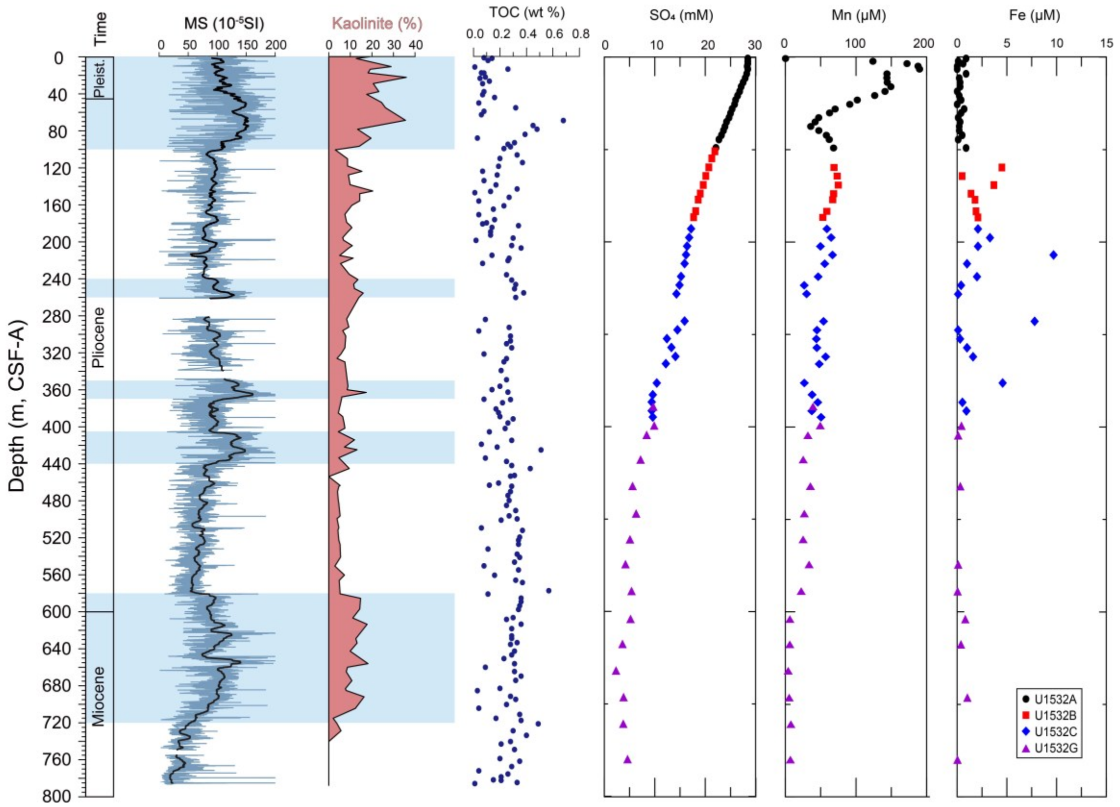

Supplementary Figure 10. Downcore variation of magnetic susceptibility[16], kaolinite content[16], total organic carbon[16], interstitial water sulfate[16], Mn and Fe concentrations[16] at Site U1532. Blue bands mark intervals of elevated MS and kaolinite content. The full record (0-800 m) of Site U1532 is presented here to clarify that downcore dissolution does not affect the characterization of magnetic concentration and grain size based on magnetic parameters. The focus of this study is the upper ~0-300 m core. MS: magnetic susceptibility; TOC: total organic carbon.

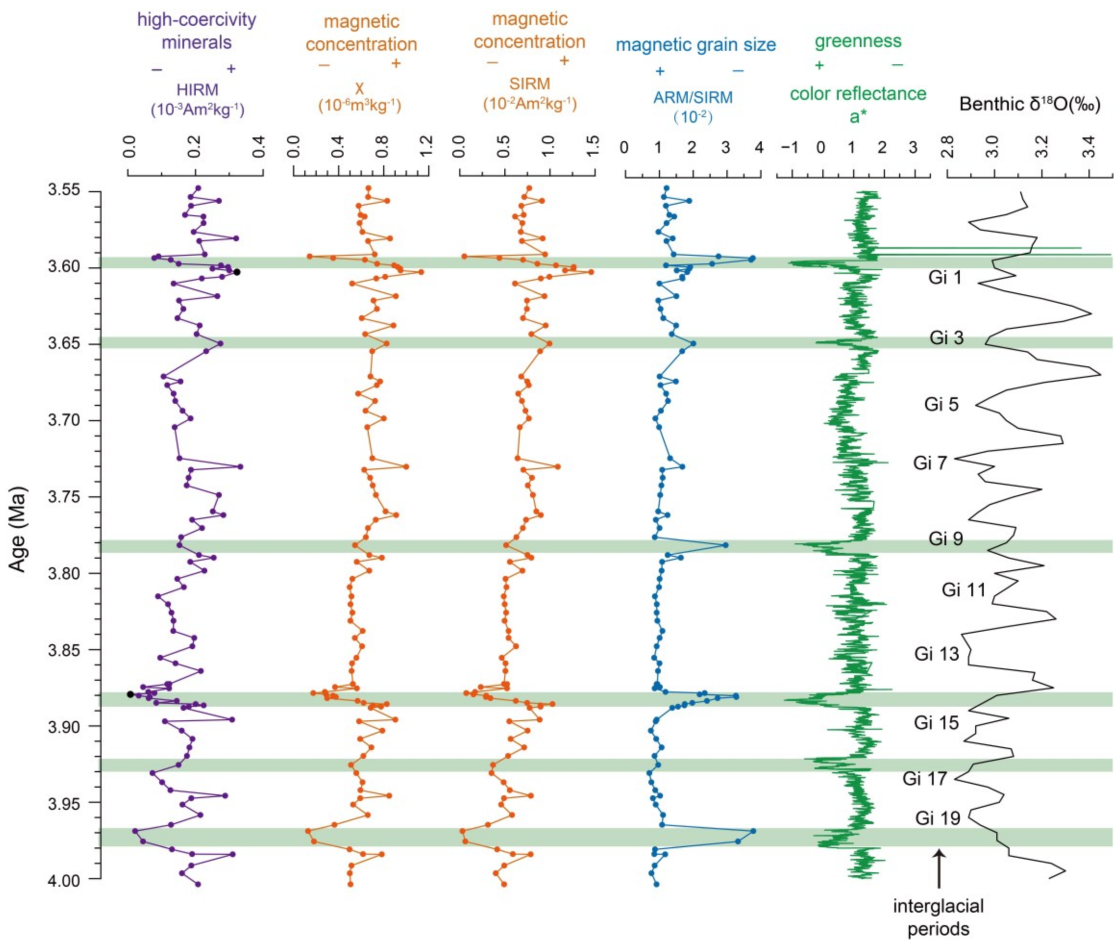

Supplementary Figure 11. Downcore variation of environmental magnetic parameters at 3.55-4.00 Ma (137.97-219.55 m of the core) at Site U1532. Magnetic mineralogy indicator hard isothermal remanent magnetization (HIRM), magnetic concentration indicators mass magnetic susceptibility (χ) and saturation isothermal remanent magnetization (SIRM), magnetic grain size indicator anhysteretic remanent magnetization (ARM)/SIRM. Shipboard color reflectance a*[16]. Global benthic $\delta^{18}O$ curve[3], containing interglacial periods of Gilbert (Gi) 1-19. Green bars in the figure, which indicate greenish layers in the core, were identified based on low values of the color reflectance a*. Lithofacies cyclicity was observed along the U1532 core[1]. Thin greenish muddy units were characterized by the presence of siliceous microfossils, bioturbation, and ice-rafted debris (IRD)[1]. These greenish units have been interpreted to represent interglacial deposits[1]. They alternate with thick, gray laminated, predominantly terrigenous silty-clay units interpreted to represent glacial periods[1]. In the greenish layers, the low HIRM values indicate low concentration of high-coercivity magnetic minerals. The low values of χ and SIRM indicate low concentration of magnetic minerals. The high values of ARM/SIRM suggest an increase of fine-grained magnetic minerals. In summary, the magnetic minerals in the greenish layers are characterized by low concentration and small grain size. This indicates that the magnetic mineral indicators at core depths of 137.97-219.55 m exhibit a clear glacial-interglacial variation pattern.

Component	Section C average (pre-3.2 Ma)	Section B average (post-3.2 Ma)	Change
**Opal**	3.53%	1.88%	-1.65%
**$CaCO_3$**	2.25%	0.23%	-2.02%
**TOC**	0.20%	0.25%	+0.05%
**Biogenic material**	~5.98%	~2.36%	-3.62%
**Terrigenous (balance)**	~94.02%	~97.64%	+3.62%

Relative increase in terrigenous fraction:

- Section C: 94.02%
- Section B: 97.64%
- Relative increase = (97.64 - 94.02) / 94.02 = **3.62% / 94.02 ≈ 3.85%**

Theoretical maximum contribution to ARM:

- ARM is derived from the terrigenous fraction, and assuming that the ARM intensity of the terrigenous fraction remains constant
- Then ARM concentration is proportional to the terrigenous proportion
- Maximum theoretical ARM increase = **3.85%**

Observed ARM increase:

- Section C mean ARM: 1×10^{-4} Am^2/kg
- Section B mean ARM: 3×10^{-4} Am^2/kg
- Observed increase = (3 - 1) / 1 × 100% = **200%**

Comparison:

- Theoretical maximum dilution contribution: **3.85%**
- Observed ARM increase: **200%**
- Observed increase exceeds the theoretical maximum by a factor of **>50**

Supplementary Figure 12. Calculation of influence on ARM by biogenic dilution effect. Calcium carbonate and total organic carbon (TOC) contents were obtained from shipboard measurements[16]. ARM: anhysteretic remanent magnetization.

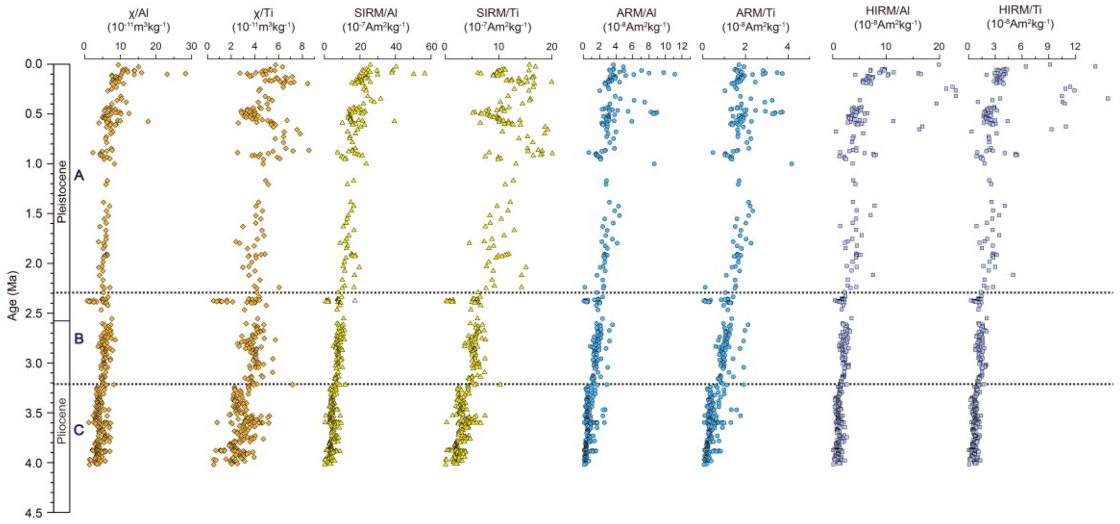

Supplementary Figure 13. Normalized concentration parameters to conservative terrigenous elements Al and Ti. χ: mass magnetic susceptibility; SIRM: saturation isothermal remanent magnetization; ARM: anhysteretic remanent magnetization; HIRM: hard isothermal remanent magnetization.

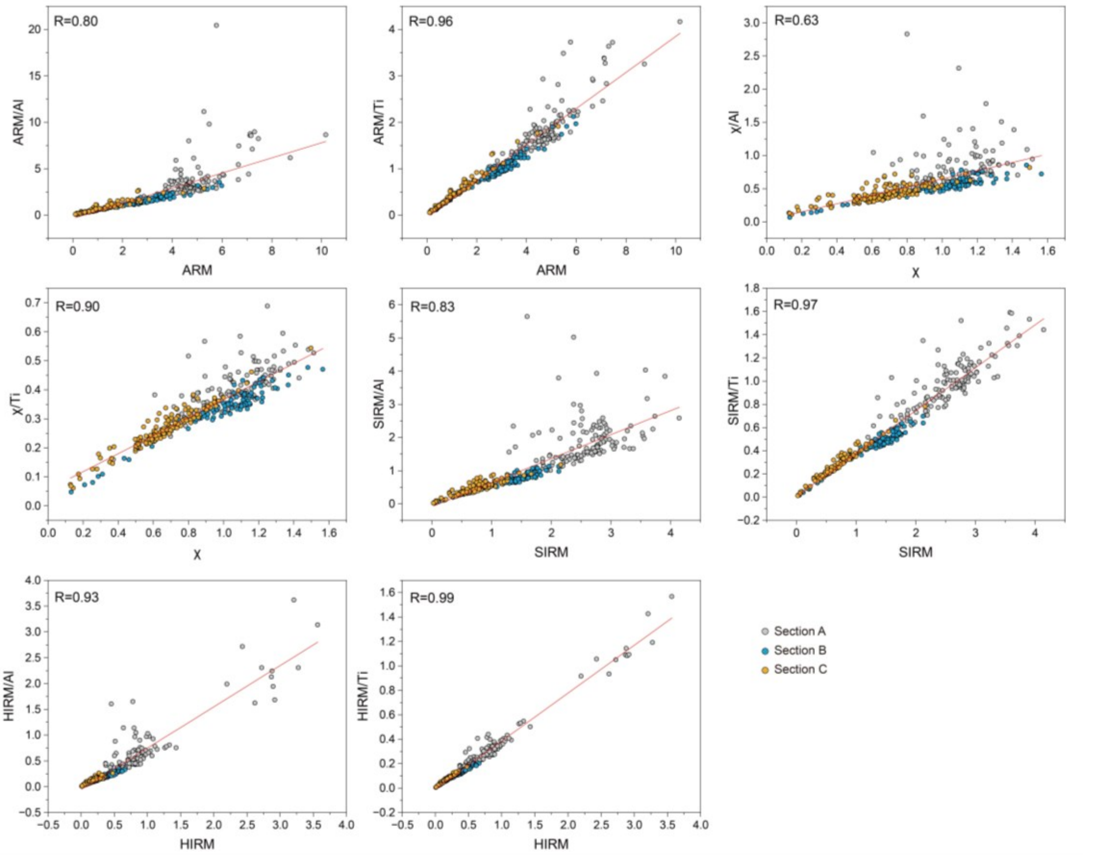

Supplementary Figure 14. Correlation of normalized concentration parameters with conservative terrigenous elements Al and Ti. R: Pearson's correlation coefficient. χ: mass magnetic susceptibility; SIRM: saturation isothermal remanent magnetization; ARM: anhysteretic remanent magnetization; HIRM: hard isothermal remanent magnetization.

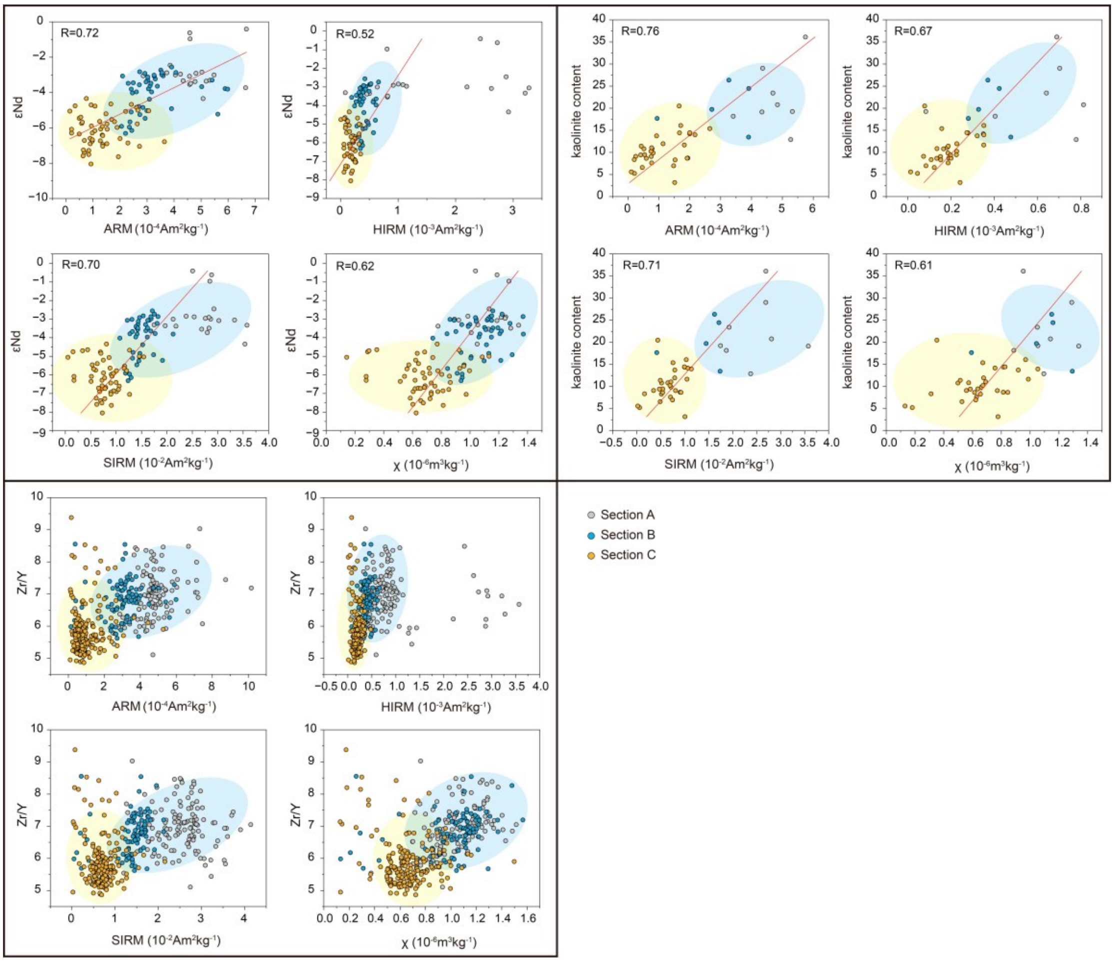

Supplementary Figure 15. Correlation of magnetic concentration-related parameters with detrital sediment Nd isotope compositions (ε_{Nd}), kaolinite content and Zr/Y. Yellow shading denotes the range of most data points before 3.2 Ma, and blue shading denotes the range after 3.2 Ma. Kaolinite content data is from[16]. Data of detrital sediment Nd isotope compositions (ε_{Nd}) is from[2,17]. R: Pearson's correlation coefficient.

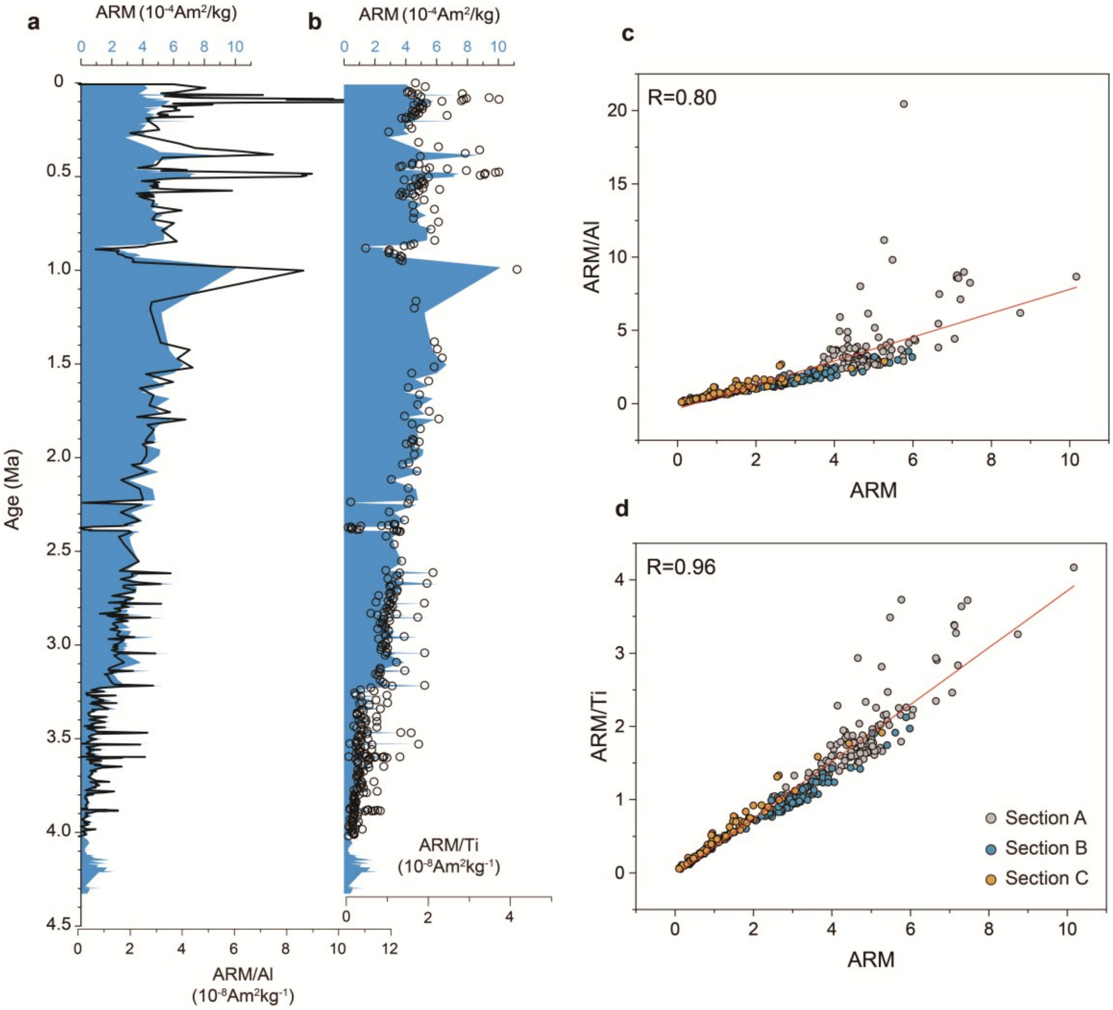

Supplementary Figure 16. Consistent long-term trends between anhysteretic remanent magnetization (ARM) and terrigenous-normalized magnetic parameters (ARM/Al, ARM/Ti). (a) Comparison of anhysteretic remanent magnetization (ARM, blue shading) and ARM/Al (black line). (b) Comparison of ARM (blue shading) and ARM/Ti (black circles). (c) Bi-plot of ARM vs. ARM/Al. (d) Bi-plot of ARM vs. ARM/Ti. R: Pearson's correlation coefficient.

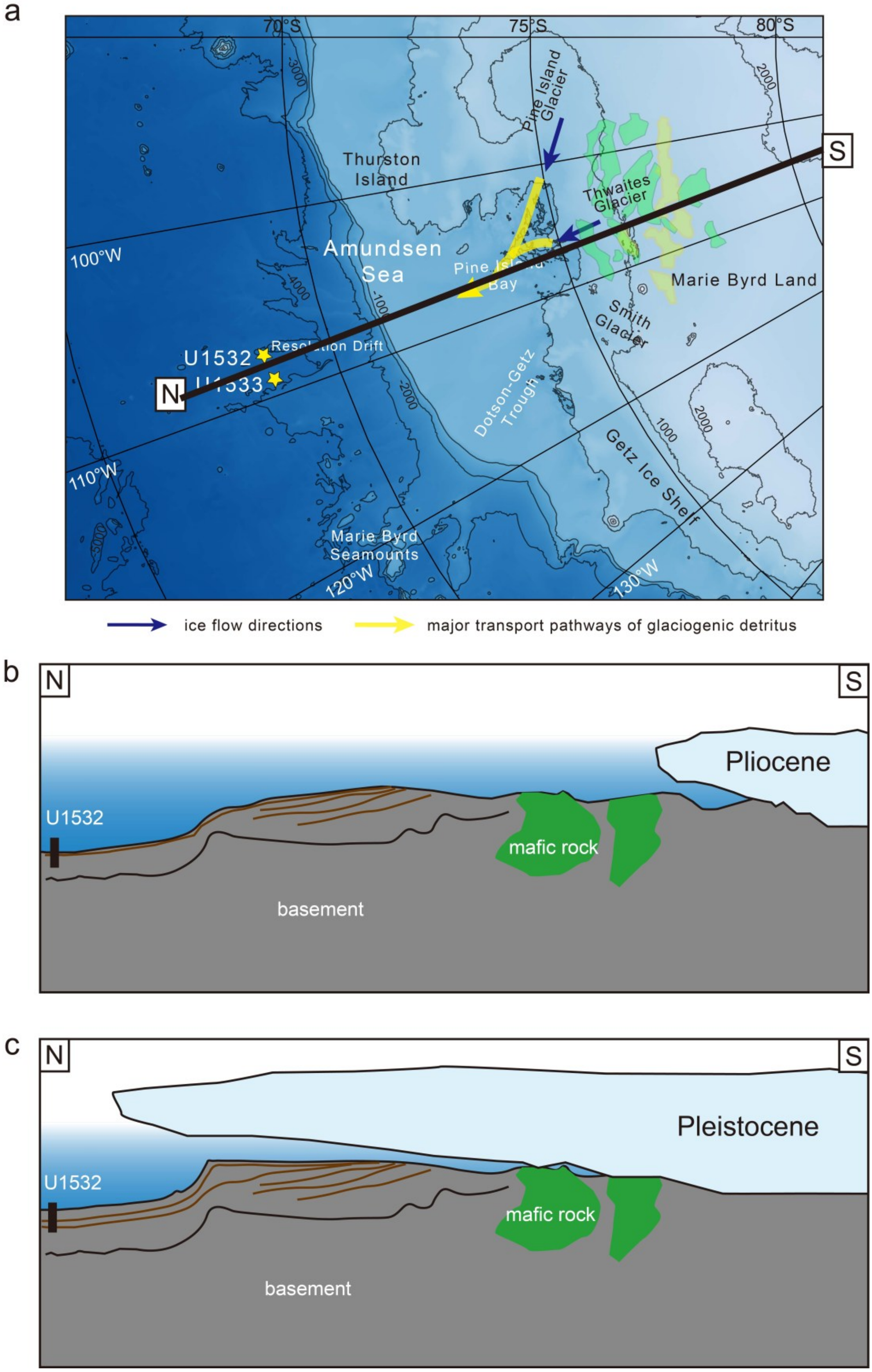

Supplementary Figure 17. Schematic diagram of the change in ice-sheet growth and erosion patterns from the Pliocene to the Pleistocene. (a) A regional map of the Amundsen Sea Embayment (ASE) and coastal Marie Byrd Land (MBL). Resolution Drift in the Amundsen Sea sector and the drill

sites (U1532 and U1533, yellow stars) of the International Ocean Discovery Program (IODP) Expedition 379 are shown. Colors and contours show elevation relative to sea level derived from International Bathymetric Chart of the Southern Ocean (IBCSO) v2[18]. IBCSO v2 data are licensed under a Creative Commons Attribution 4.0 International License (https://creativecommons.org/licenses/by/4.0/). Changes were made for region cropping and color scheme. Negative contour values represent bathymetry (water depth), whereas positive values represent ice sheet surface elevation. Blue arrows indicate the ice flow directions of the major ice streams. Green shaded areas represent mafic intrusive rocks, and yellow shaded areas represent sedimentary basins, based on Jordan et al.[19]. The black line delineates the extents of the schematic diagrams in (b) and (c). (b) A smaller ice sheet in the Pliocene. (c) An advanced ice sheet in the Pleistocene. The larger Pleistocene ice sheet eroded the mafic intrusive rocks and sedimentary basins beneath Thwaites Glacier, transporting substantial magnetite to Site U1532.

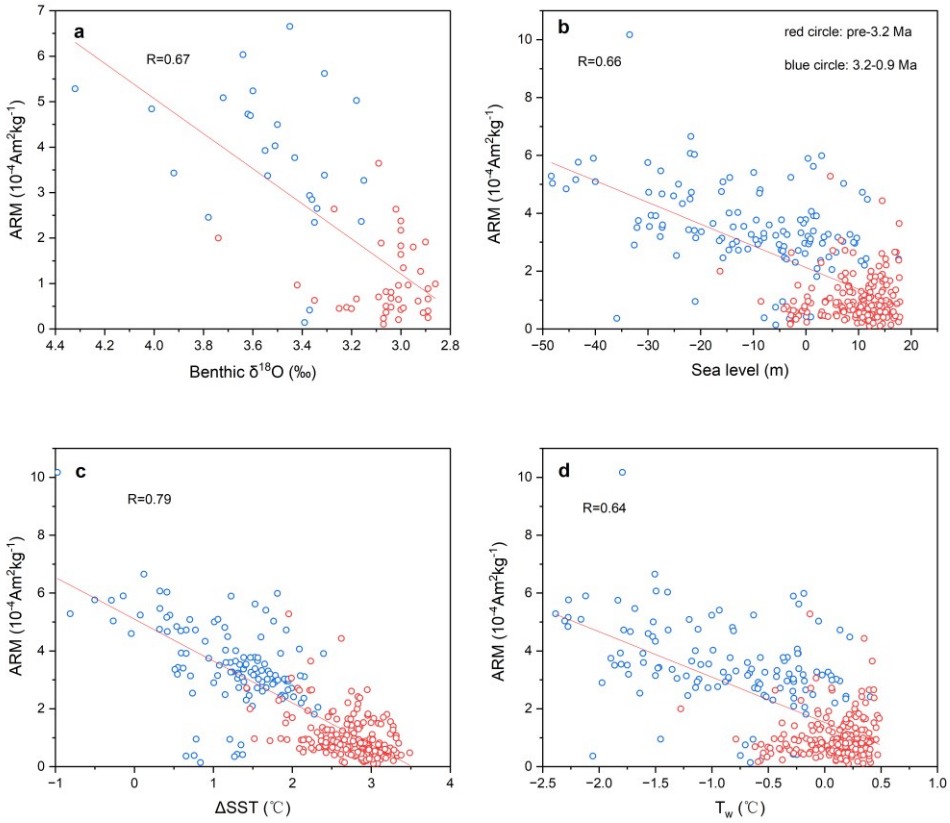

Supplementary Figure 18. Correlation of anhysteretic remanent magnetization (ARM) with global climate proxies. (a) Anhysteretic remanent magnetization (ARM) vs. benthic $\delta^{18}O$[3]; (b) ARM vs. sea level[20]; (c) ARM vs. global sea surface temperature change (Δ SST)[21]; (d) ARM vs. deep ocean temperature (T_w)[20]. R: Pearson's correlation coefficient.

Supplementary Text 1. Determination of dominant control on HIRM and S_{-300}

An assessment of hard isothermal remanent magnetization (HIRM) using a lower reference magnetic field (calculation of the L-ratio[6]) was used to determine the interpretation of HIRM and S_{-300} (Supplementary Fig. 1). Several notably high HIRM values are observed in section A, all occurring during glacial periods (Supplementary Figs. 1a and 1d). These peaks are accompanied by low S_{-300} and high SIRM/χ ratios, indicating an increased content of high-coercivity minerals[7]. Given the increased supply of terrigenous high-coercivity minerals during cold intervals with ice advance, the anomalously high HIRM values in section A likely resulted from combined effects of enhanced terrigenous input and oxidative conditions. The lack of correlation between L-ratio and HIRM indicates that HIRM and S_{-300} variations are primarily controlled by changes in antiferromagnetic mineral concentration rather than by variations in coercivity distribution (Supplementary Figs. 1b and 1c)[6].

Supplementary Text 2. Results of IRM acquisition curves, hysteresis and FORCs

To further confirm the types of magnetic minerals, we measured isothermal remanent magnetization (IRM) acquisition curves, hysteresis and first-order reversal curves (FORCs) for representative samples of three sections (Methods; Supplementary Figs. 2 and 3). Coercivity components can be decomposed from the first-order derivative of IRM acquisition curves[22,23]. A very-low-coercivity component (< 6 mT) and a very-high-coercivity component (> 0.4 T) are required for optimal fitting. The very-low-coercivity component could be ultrafine magnetite due to the influence of short relaxation times[24] or very coarse-grained multi-domain (MD) particles because their domain walls are easily moved at low field strengths. The very-high-coercivity component should be antiferromagnetic minerals. In the samples of section A, three major components are present (Fig. 4a, Supplementary Figs. 2a and 3), including a broad low-coercivity (<50 mT) component (component L), a narrow medium-coercivity component (component M), and a narrow high-coercivity (>100 mT) component (component H). A dispersion parameter (DP) closes to 0.2 is generally regarded as characteristic of magnetofossils, and a DP greater than 0.3 is detrital magnetite[25]. Component L and M should be detrital magnetite and magnetofossils, respectively. The coercivity of component H, along with the low S-100 values, suggests the presence of maghemite. Maghemitization occurring at the surface of magnetite grains leads to a lattice mismatch, which increases the internal stress within the magnetic particles, and causes increased coercivity of remanence[26]. Similar component was observed in Quaternary drift sediments off the Antarctic Peninsula[27] and in sediment core NGC86 from the Ontong-Java Plateau[28]. In sections B and C, two dominant components are observed, resembling L and M components, while component H disappears (Fig. 4a, Supplementary Figs. 2a and 3).

Hysteresis loops and FORCs are effective in discriminating magnetic mineral coercivity, grain size distribution and various magnetic interactions and their strength[29,30]. Consistent with the IRM decomposition, the hysteresis results of the samples from section A show different characteristics from sections B and C (Fig. 4b, Supplementary Figs. 2b and 5a). The higher values of Bcr in section A are consistent with the presence of maghemite and hematite supported by χ-T and three-axis thermal demagnetization results. The increased M_{rs}/M_s ratio suggests an increased proportion of SD particles.

The coarser terrigenous magnetic particles contribute to the broad flanks of the FORC distributions in sections B and C[29]. The steep vertical distributions shown in the lower left corner of FORC diagrams suggest the presence of SP particles[29]. FORC diagrams contain an obvious central ridge component along $B_u = 0$ (Fig. 4c, d, e and Supplementary Fig. 2), suggesting the presence of non-interacting SD particles in sections A and B (most likely magnetofossils)[13,31]. The low magnetization and inconspicuous central

ridge in samples from section C imply low magnetic concentration and few non-interacting SD particles[13,31].

Supplementary Text 3. Results of high-temperature-dependence of magnetic susceptibility curves

The high-temperature-dependence of magnetic susceptibility (χ-T) curves exhibit a significant decrease in susceptibility at about 590°C, which corresponds to the Curie point of magnetite[32] (Supplementary Fig. 4). We attribute the susceptibility rise of heating curves below 272°C to gradual unblocking of fine-grained (SP/SD) ferrimagnetic particles[33]. An asymmetric "hump" in the heating curves between 200-400°C, similar to that observed in Antarctic Peninsula drift sediments[27], suggests the presence of maghemite, which dissociates into magnetite and other products in an inert atmosphere. Inversion temperatures of maghemite to magnetite in an inert atmosphere range from 250°C to 900°C, depending on crystallinity, impurities, particle morphology, and grain size[27,34,35]. The subsequent increase in susceptibility is affected by both the thermal decomposition of iron-containing salts/clay minerals and the effect of magnetic particles unblocking[36]. The much higher χ values during cooling prove the production of new magnetite. The χ-T results indicate that magnetite is the major contributor to the susceptibility of the sediments, and maghemite is present in section A.

Supplementary Text 4. Results of Lowrie's three-axis thermal demagnetization

Higher values of B_c and B_{cr} of samples from section A compared to those from Sections B and C, indicating a greater contribution of high-coercivity minerals in Section A (Supplementary Fig. 5a). IRM acquisition curves of samples from sections B and C climb more steeply in 0-200 mT and reach saturation before 300 mT, suggesting a lack of hard magnetic minerals (Supplementary Fig. 5b). In contrast, the IRM curves of Section A samples do not saturate fully at high fields, consistent with the presence of high-coercivity minerals. Three-axis thermal demagnetization of the soft (<0.12 T), medium (0.12-0.4 T) and hard (0.4-2.4 T) coercivity fractions shows different unblocking temperatures (Supplementary Fig. 5c). In all samples, more than 98% of the remanence is unblocked by 590 °C, consistent with the characteristic unblocking behavior of magnetite; only 1-2% of the remanence persists as a weak tail, which is fully unblocked between 620-640 °C[37]. These observations confirm magnetite as the dominant magnetic carrier, with the high-temperature tail consistent with minor magnetite oxidation. According to the IRM decomposition results, the coercivity of the maghemite component in section A is ~130 mT, thus contributing to the medium coercivity fractions in thermal demagnetization result. Therefore, the proportions of the medium coercivity fractions in the section A samples are greater than that in other sections. Additionally, single-domain magnetite also contributes to the medium coercivity fractions[14]. The hard fractions in section A samples unblocked at 680-690°C, indicative of hematite[32] (Supplementary Fig. 5d). In section B and C samples, the hard fractions were very low. There is no indication of goethite and pyrrhotite in samples. Therefore, the elevated SIRM/χ values (~25 kA/m) in section A should be attributed to the increase of hematite[7]. We conclude that the magnetic mineral assemblage at Site U1532 is dominated by magnetite, with Section A (uppermost 39 m of the core) containing some maghemite and hematite.

Supplementary Text 5. TEM observations results

Two samples, at depths of 24.60 m and 53.03 m, possessing two representative types of IRM decomposition and hysteresis results, were selected for transmission electron microscopy (TEM) observation to identify different types of magnetite. The TEM image, lattice fringes, selected area

electron diffraction (SAED) pattern, and energy dispersive spectra (EDS) analysis of particle ① indicate a crystal structure and chemical composition consistent with biogenic magnetite (Supplementary Fig. 7). The presence of Ti in particles ②-⑤ suggested by the EDS indicates these particles are detrital (titano)magnetite. More elongated prismatic magnetofossils with higher coercivity were observed in the sample from 24.60 m depth (Supplementary Figs. 7b, c). Less magnetofossils were observed in the sample from 53.03 m depth and most of them are equiaxial and cuboctahedral (Supplementary Figs. 7m, n, o). This is consistent with higher central ridge in FORC diagrams of section A samples compared with samples from other sections. No bullet-shaped magnetofossil was found. In section A, the linear sedimentation rate is lower than 2.3 cm/kyr[16], and the reduced detrital input makes the biogenic magnetite easier to find under the microscope. In general, magnetofossils are much rarer than detrital magnetite at Site U1532.

Supplementary Text 6. The source of magnetic minerals at Site U1532 and the evidence for negligible proportion of magnetofossils

Magnetic minerals in marine sediments typically originate from two processes: endogenous and exogenous[4]. Endogenous magnetic minerals mainly form through chemical or biogeochemical processes, such as the production of fine-grained magnetite by magnetotactic bacteria and the formation of authigenic maghemite from the oxidation of magnetite[4]. In the presence of limited oxygen, the sulfidation-reduction of iron oxides successively leads to the formation of hexagonal pyrrhotite, greigite and pyrite[38]. Exogenous magnetic minerals include terrigenous detritus, soil, aeolian dust, volcanic ash, cosmic dust, among others[4]. Our investigation of magnetic properties and TEM observations of Site U1532 sediments indicates the presence of (titano)magnetite, biogenic magnetite, authigenic maghemite and hematite. To evaluate the contribution of biogenic magnetite to magnetic data, we compare IRM contributions of detrital and biogenic magnetite as determined from IRM decomposition with χ to identify the contribution of these two different types of magnetite to the magnetic signal. We find that except for the oxidized samples in section A, the IRM contributions of detrital magnetite are positively correlated with χ (Supplementary Fig. 8a), without apparent correlation between the magnetofossils and χ (Supplementary Fig. 8b). Also, biplot of χ_{ARM}/χ_{lf} vs. χ_{ARM}/χ_{fd}% indicates that the contribution of biogenic magnetite to the magnetic signals is small (Supplementary Fig. 9). These results are consistent with the very few magnetofossils observed by TEM (Supplementary Fig. 7). Therefore, we have ignored the contribution of biogenic magnetite when discussing the sources of magnetic minerals.

Supplementary Text 7. Does the downcore dissolution of magnetic minerals affect the characterization of magnetic concentration and grain size based on magnetic parameters?

The sulfate consumption rate is extremely low at Site U1532, which might reflect low biogeochemical activity[16] (Supplementary Fig. 10). The low TOC content of the sediment (< 0.4 wt%) probably is responsible for the slow sulfate consumption at this site[16]. Sulfate reduction is so extremely low below 20 m, Mn reduction overlaps with sulfate reduction from 20 to 578 m, resulting in high Mn concentrations throughout Site U1532[16]. Fe concentrations are generally low (< 10 μM) throughout the cored section at Site U1532[16]. This indicates that the iron oxide reduction rate at Site U1532 is extremely low.

Kaolinite commonly has survived since the Devonian Period[39]. In buried sediments, the water pressure and [H_4SiO_4] requisite for stable kaolinite generally are maintained[39]. Replacement of kaolinite by dickite has been observed to occur with increasing depth of burial in sandstones from three different basins on the Norwegian continental shelf[40]. This transformation occurs at ca. 3.1-3.4 km (~120-130°C)

and demonstrates regional reproducibility, making it a reliable diagenetic paleogeothermometer[40]. Therefore, kaolinite is stable in the cores from Site U1532 at depths < 800 m. Throughout Core U1532, intervals of high magnetic susceptibility consistently correspond to those with elevated kaolinite content[16] (Supplementary Fig. 10), suggesting a common source for both the magnetic minerals and kaolinite. This also indicates that downcore dissolution of magnetic minerals is negligible. Besides, magnetic parameters exhibit a distinct glacial-interglacial pattern at the depth of 137.97-219.55 m (3550-4000 ka; Supplementary Fig. 11). This also demonstrates the reliability of environmental magnetic proxies at Site U1532. Additionally, the strong correlations between the single-domain magnetite concentration indicator ARM and the provenance proxies detrital ε_{Nd} and Zr/Y indirectly indicate that fine-grained magnetite is well preserved (Fig. 4).

In summary, we cannot rule out the presence of downcore iron oxide dissolution, but it is minimal and does not affect the characterization of magnetic concentration and grain size based on magnetic parameters.

Supplementary Text 8. Age control for correlation between magnetic and detrital ε_{Nd} data.

The detrital ε_{Nd} data used in this study are from Rahaman et al.[2] and Horikawa et al.[17]. The samples with ε_{Nd} data do not correspond exactly in depth to those with magnetic measurements. To assess the correlation between ε_{Nd} and magnetic parameters, we selected 117 data pairs. The age offsets between each pair are consistently small: all are less than 5 kyr, with 87 pairs having offsets <2 kyr and only four pairs exceeding 4 kyr. The age offset for each data pair is provided along with the data in the Source Data file. As our study focuses on million-scale variations in magnetic properties at Site U1532, these minor age differences (mostly within 2 kyr) have a negligible effect on the interpretation.

Supplementary References

1. Gohl, K. & Wellner, S. J. Expedition 379 summary. *Proceedings of the International Ocean Discovery Program*. **379**, 1-21 (2021).
2. Rahaman, W., Gutjahr, M. & Prabhat, P. Late Pliocene growth of the West Antarctic Ice Sheet to near-modern configuration. *Nat. Commun.* **16**, 6705 (2025).
3. Lisiecki, L. E. & Raymo, M. E. A Pliocene-Pleistocene stack of 57 globally distributed benthic $\delta^{18}O$ records. *Paleoceanography*. **20**, PA1003 (2005).
4. Thompson, R. & Oldfield, F. *Environmental magnetism*. (Allen and Unwin, London, UK, 1986).
5. Bloemendal, J. Rock magnetism of Late Neogene and Pleistocene deep-sea sediments: Relationship to sediments source, diagenestic processes and sediment lithology. *J. Geophys. Res. Solid Earth*. **97**, 4361-4375 (1992).
6. Liu, Q., Roberts, A. P., Torrent, J., Horng, C. & Larrasoaña, J. C. What do the HIRM and S-ratio really measure in environmental magnetism? *Geochem. Geophys. Geosyst.* **8**, Q09011 (2007).
7. Peters, C. & Dekkers, M. J. Selected room temperature magnetic parameters as a function of mineralogy, concentration and grain size. *Phys. Chem. Earth*. **28**, 659-667 (2003).
8. Stoner, J. S. & St-Onge, G. Chapter Three Magnetic Stratigraphy in Paleoceanography: Reversals, Excursions, Paleointensity, and Secular Variation. *Proxies in Late Cenozoic Paleoceanography*: Elsevier; 2007. pp. 99-138.
9. Dearing, J. A. et al. Frequency-dependent susceptibility measurements of environmental materials. *Geophys. J. Int.* **124**, 228-240 (1996).
10. King, J., Banerjee, S. K., Marvin, J. & Özdemir, Ö. A comparison of different magnetic methods

for determining the relative grain size of magnetite in natural material: some results from lake sediments. *Earth Planet. Sci. Lett.* **59**, 404-419 (1982).

11. Frederichs, T., Bleil, U., Däumler, K., von Dobeneck, T. & Schmidt, A. M. The magnetic view on the marine paleoenvironment: Parameters, techniques and potentials of rock magnetic studies as a key to paleoclimatic and paleoceanographic changes. *Use of Proxies in Paleoceanography*, 575-599 (1999).
12. Maxbauer, D. P., Feinberg, J. M. & Fox, D. L. MAX UnMix: A web application for unmixing magnetic coercivity distributions. *Comput. Geosci.* **95**, 140-145 (2016).
13. Egli, R., Chen, A. P., Winklhofer, M., Kodama, K. P. & Horng, C. Detection of noninteracting single domain particles using first-order reversal curve diagrams. *Geochem. Geophys. Geosyst.* **11**, GC002916 (2010).
14. Lowrie, W. Identification of ferromagnetic minerals in a rock by coercivity and unblocking temperature properties. *Geophys. Res. Lett.* **17**, 159-162 (1990).
15. Oldfield, F. Toward the discrimination of fine-grained ferrimagnets by magnetic measurements in lake and near-shore marine sediments. *J. Geophys. Res. Solid Earth*. **99**, 9045-9050 (1994).
16. Wellner, J. S. et al. Site U1532. *Amundsen Sea West Antarctic Ice Sheet History, Proceedings of the International Ocean Discovery Program*. **379**, 1-47 (2021).
17. Horikawa, K. et al. Repeated major inland retreat of Thwaites and Pine Island glaciers (West Antarctica) during the Pliocene. *Proc. Natl. Acad. Sci. U. S. A.* **123**, e1786626174 (2026).
18. Dorschel, B. et al. The International Bathymetric Chart of the Southern Ocean Version 2 (IBCSO v2). *Sci. Data*. **9**, 1-13 (2022).
19. Jordan, T. A., Thompson, S., Kulessa, B. & Ferraccioli, F. Geological sketch map and implications for ice flow of Thwaites Glacier, West Antarctica, from integrated aerogeophysical observations. *Sci. Adv.* **9**, eadf2639 (2023).
20. Rohling, E. J. et al. Comparison and Synthesis of Sea-Level and Deep-Sea Temperature Variations Over the Past 40 Million Years. *Rev. Geophys.* **60**, e2022RG000775 (2022).
21. Clark, P. U., Shakun, J. D., Rosenthal, Y., Köhler, P. & Bartlein, P. J. Global and regional temperature change over the past 4.5 million years. *Science*. **383**, 884-890 (2024).
22. Egli, R. Characterization of Individual Rock Magnetic Components by Analysis of Remanence Curves, 1. Unmixing Natural Sediments. *Stud. Geophys. Geod.* **48**, 391-446 (2004).
23. Kruiver, P. P., Dekkers, M. J. & Heslop, D. Quantification of magnetic coercivity components by the analysis of acquisition curves of isothermal remanent magnetisation. *Earth Planet. Sci. Lett.* **189**, 269-276 (2001).
24. Heslop, D., McIntosh, G. & Dekkers, M. J. Using time- and temperature-dependent Preisach models to investigate the limitations of modelling isothermal remanent magnetization acquisition curves with cumulative log Gaussian functions. *Geophys. J. Int.* **157**, 55-63 (2004).
25. Egli, R. Characterization of individual rock magnetic components by analysis of remanence curves, 3. Bacterial magnetite and natural processes in lakes. *Phys. Chem. Earth*. **29**, 869-884 (2004).
26. Dunlop, D. J. Hysteresis properties of magnetite and their dependence on particle size: A test of pseudo-single-domain remanence models. *J. Geophys. Res. Solid Earth*. **91**, 9569-9584 (1986).
27. Channell, J. E. T., Xuan, C., Hodell, D. A., Crowhurst, S. J. & Larter, R. D. Relative paleointensity (RPI) and age control in Quaternary sediment drifts off the Antarctic Peninsula. *Quat. Sci. Rev.* **211**, 17-33 (2019).
28. Yamazaki, T. & Solheid, P. Maghemite-to-magnetite reduction across the Fe-redox boundary in a

sediment core from the Ontong-Java Plateau: Influence on relative palaeointensity estimation and environmental magnetic application. *Geophys. J. Int.* **185**, 1243-1254 (2011).

29. Roberts, A. P., Pike, C. R. & Verosub, K. L. First-order reversal curve diagrams: A new tool for characterizing the magnetic properties of natural samples. *J. Geophys. Res. Solid Earth*. **105**, 28461-28475 (2000).
30. Pike, C. R., Roberts, A. P. & Verosub, K. L. Characterizing interactions in fine magnetic particle systems using first order reversal curves. *J. Appl. Phys.* **85**, 6660-6667 (1999).
31. Roberts, A. P., Chang, L., Heslop, D., Florindo, F. & Larrasoaña, J. C. Searching for single domain magnetite in the "pseudo-single-domain" sedimentary haystack: Implications of biogenic magnetite preservation for sediment magnetism and relative paleointensity determinations. *J. Geophys. Res. Solid Earth*. **117**, 1-26 (2012).
32. Dunlop, D. J. & Özdemir, Ö. *Rock Magnetism: Fundamentals and Frontiers*. (Cambridge University Press, 1997).
33. Deng, C. et al. Mineral magnetic variation of the Jiaodao Chinese loess/paleosol sequence and its bearing on long-term climatic variability. *J. Geophys. Res. Solid Earth*. **110**, B03103 (2005).
34. De Boer, C. B. & Dekkers, M. J. Grain-size dependence of the rock magnetic properties for a natural maghemite. *Geophys. Res. Lett.* **23**, 2815-2818 (1996).
35. Gendler, T. S. et al. The lepidocrocite-maghemite-haematite reaction chain-I. Acquisition of chemical remanent magnetization by maghemite, its magnetic properties and thermal stability. *Geophys. J. Int.* **160**, 815-832 (2005).
36. Hunt, C. P. Rock-magnetic proxies of climate change in the loess-palaeosol sequences of the western Loess Plateau of China. *Geophys. J. Int.* **123**, 232-244 (1995).
37. Readman, P. W. & O'Reilly, W. Magnetic Properties of Oxidized (Cation-Deficient) Titanomagnetites (Fe, Ti, _??_) 4_3O_4. *J. Geomagn. Geoelectr.* **24**, 69-90 (1972).
38. Sweeney, R. E. & Kaplan, I. R. Pyrite framboid formation: laboratory synthesis and marine sediments. *Econ. Geol.* **68**, 618-634 (1973).
39. Hurst, V. J. & Kunkle, A. C. Dehydroxylation, rehydroxylation, and stability of kaolinite. *Clay Clay Min.* **33**, 1-14 (1985).
40. Ehrenberg, S. N., Aagaard, P., Wilson, M. J., Fraser, A. R. & Duthie, D. M. L. Depth-dependent transformation of kaolinite to dickite in sandstones of the Norwegian continental shelf. *Clay Miner.* **28**, 325-352 (1993).
