# Supplementary material for: West Antarctic Ice Sheet advance since the early Pliocene
Source: Nat Commun. 2026 Jun 6;17:7240. doi: 10.1038/s41467-026-74100-1 (PMC13396233; doi:10.1038/s41467-026-74100-1)
Supplement: Supplementary file 2 — Transparent Peer Review file [file 41467_2026_74100_MOESM2_ESM.pdf]

nature portfolio

Peer Review File

West Antarctic Ice Sheet advance since the early Pliocene

Corresponding Author: Professor Liang Gao

This file contains all reviewer reports in order by version, followed by all author rebuttals in order by version.

Attachments originally included by the reviewers as part of their assessment can be found at the end of this file.

Version 0:

Reviewer comments:

Reviewer #1

(Remarks to the Author)
This manuscript builds upon a relationship between magnetic parameters and kaolinite in the IODP U1532 sediment core, from the Amundsen Sea, to interpret ice sheet dynamics in the Amundsen Sector over the Pliocene and Pleistocene. The interpretations are rooted in a correlation between Kaolinite and magnetic properties. Prior studies have reported a kaolinite source beneath the Ice Sheet in the Amundsen hinterland. Periods with greater magnetic concentrations are interpreted to reflect cold periods where the ice sheet was grounded on the outer shelf, enhancing the delivery of continental detritus downslope, while periods with low magnetic concentration reflect open ocean deposition and interglacial conditions. The authors present evidence to suggest that the sector has undergone sustained cooling, concentrated in specific intervals at 3.8, 3.2 , 2.4, and 1.6 Ma. Further, they suggest that a period of sustained warmth in the Pliocene (from 4.1 to 3.8 Ma) is a most likely candidate for substantial collapse of the ice sheet, and highlight a global temperature influence on glacial dynamics in the region. In my view, the approach is suitable and the breadth of data called upon is commendable. Conclusions are largely well supported by the data and interpretative framework which they provide.

I have not found any major issues to challenge the validity of the study. However, I frequently struggled to understand the key message of the authors, and think the manuscript as a whole could benefit from further grammatical corrections and restructuring. This is particularly so for the introduction and conclusion, where the former is of utmost importance to interest the reader and provide essential context.

Secondly, the Results section currently presents a mixture of results (magnetic parameter values) and interpretations (what they suggest about the magnetic mineralogy). These could be more clearly separated in my opinion, though it may be an issue of personal preference. Overall, the results section is particularly dense and difficult to read, due in part to the sheer quantity of technical terms and acronyms. Without a coherent line of thought to guide me, I had the feeling that it was an assortment of observations lacking logical progression. I concede that this may be due to my own acuity, and could possibly be no obstacle to a more intelligent member of the community. Nonetheless, please earnestly explore how to optimally structure this passage in order to clearly, concisely, and simply convey the essential information for the study.

In summary, I find this work to be a fundamentally valuable addition to the current literature, and do not find any reason to majorly challenge its methods, interpretations or conclusions within. However, the manuscript is currently held back from its full potential by a lack of clarity or logical flow in many of its arguments. The literary proficiency should not be an unsurpassable barrier for publication as it does not undermine the rigor of science within, however an improvement would drastically benefit the accessibility of the manuscript to a wider audience. I have done my best to catalogue the (mostly minor) issues in detail, line by line.

Finally, I apologise for any comments deriving from a clear misunderstanding of the text. Where noted, please use these as an opportunity to clarify the main text to prevent other readers from reaching similarly false conclusions.

Line by line revisions:
Abstract:
The general structure of the abstract could be refined to make it more clear which specific investigations the authors have completed on U1532 in this study. For example, lines 44 – 48 state significant findings on the variable provenance to the

Amundsen, however it is unclear which evidence/proxies these are drawn from this study or simply provide context.

Line 50: Grammatical suggestion: “The Anhysteretic Remanent Magnetization of sediment serves as a reliable proxy …”
Line 52: The term “climate deterioration” is unclear, especially in Antarctica where one could mistakenly attribute it to periods of ice sheet collapse. This term should be replaced to clearly identify a transition to colder climate state, or use a neutral term such as “environmental transitions”.

Introduction:
The introduction generally covers the essential content, though could do with some revision to better draw in the reader, and improve upon general clarity and logical flow of ideas. It could be beneficial to introduce Kaolinite since the interpretation of it is a fundamental aspect which underpins the study.

Line 58: While factual, this introductory sentence can be improved to draw in the reader. The link between interglacial climate state and cryospheric influence on sea level rise is not immediately clear.
Line 60: The absolute level of stability of the WAIS, and required forcing to trigger true instability, remain somewhat disputed. Thus, “unstable” could be rephrased as “more sensitive” or similar, as this is less absolute.
Line 67-68: It is unclear whether “.. and their retreat rate can be even higher than is observed today” refers to future projections or past observations. Please rephrase to clarify that this this refers to early observations.
Line 72: “the WAIS history” is redundant.
Line 75 – 79: I suggest to split this sentence into a few. The first phrase on 14C may also be improved for clarity, eg. "14C ages in sediment cores from the inner ASE shelf indicated that grounding line of the WAIS had retreated [from the outer shelf?] by the start of the Holocene”
Line 81: Suggestion: “Cores obtained by the sea floor drill rig…”
Line 82: of [the] West Antarctica
Figure 1: Great figure. Please add mention of the bathymetric contour intervals, or label them. The bathymetry scale could be cropped to ~5000 m. Are the contours on the continent representing bed morphology, ice morphology, or both? The context map labels a suite of other ODP/IODP cores around the continent – are these mentioned in the text? The core location icons could be slightly more prominent, but not essential.

Geological Setting & Analyses
Line 104: Keep consistency between ca. and ~. Grammatical suggestion: Continental rise Site U1532 is located on the continental rise at the crest of the elongated Resolution Drift, ca. 270 km north of the ASE shelf edge.
Line 105: Clarify that this refers to a sediment coring depth of 794 m below the sea floor.
Line 106: Please reference Gohl et al 2021 (ref # 24) for core-bottom age.
Line 107: … gravity driven processes [such as] slumps and turbidity currents …
This section could benefit by further addressing various sediment delivery pathways from the continent to the continental rise, as this will be essential for later conclusions. At least, please elaborate on turbidity currents and their specific origin. Can they be meltwater derived / tidally eroded sediments, or only originate via slope failure in the ASE?
Line 113-14: This is methodological. I would suggest either to remove the sentence, or move it to the end of the paragraph where it follows the age model, and leads to the following results section.
Line 115 – 118: This can be gently reworked, grouping separate ideas into smaller sentences for easier reading.

Methods
Paleomagnetism and XRF techniques are appropriate and sufficiently well documented in my opinion. As I have no experience with TEM measurement or analyses, I will leave any critique of this method to other more qualified reviewers.

Line 469: Kappabridge. I’m unsure whether “Magnetic Susceptibility System” is required here.
Line 477: Reference Liu et al. 2007 (G3) for L-ratio (ref # 34).
Line 504-505: Elements are not proper nouns, and so do not need to be capitalized.
Line 514: Precipitation? Do you mean settling - or am I missing something?

Results
Environmental Magnetism
Table 1: Please ensure every sentence containing an established interpretation is cited, even those most basic claims (such as the first sentences of SIRM and L-Ratio)
L-ratio: Variable L-ratios rather correspond to changing coercivity of Hematite, which is itself associated with stoichiometry, density and/or grainsize (but not solely grainsize; Liu et al 2007, G3). It can thus indicate changes in Hematite provenance. Please clarify these additional possible controls on L-ratio in this interpretation.
Volumetric MS and Mass MS: For non-experts in the field, please clarify that grain size refers to magnetic mineral grain size, not whole sediment grainsize. (I later note that this is somewhat addressed in Line 126, but could possibly be restated - if you agree).
SIRM/x: I would suggest to rephrase “useful for assessing mineralogy” to something like “Indicator for magnetic mineralogy”. There is greater complexity in SIRM/x beyond the influences of (titano)magnetite and maghemite, with an additional effect of grainsize for (titano)magnetite (c.f. Figure 3 from Peters and Dekkers, 2003). Additionally, in this plot, Maghemite overlaps with the range of (titano)magnetite at ~10 mAm-1. Please carefully recheck this section.

Line 133 (Figure 2): Greenish layers (reflecting interglacial ‘periods’)
“Diatom Relative Abundance” or “Relative Diatom Abundance”

SIRM/x (kA m -1) – also see prior comment that Maghemite is also ~10 kA m-1
From 100 – 290 m, the “greenish layers” are relatively clear in a* color reflectance, but less-so in the upper 100 m. I assumed (based on line 115-117) that these “greenish layers” were obtained from the Gohl expedition report, but while mentioned there I do not find a detailed catalogue of their occurrences like you present here. Please clarify the criteria for their classification and include them in the figure caption.
Red annotations “a” and “b” are not included in the figure caption.
Line 153 – 155: L-ratio is stable throughout Section A and B, thus HIRM is indicative changing concentrations in this interval. But sections C-F have more variable L-ratios as illustrated in Figure S2. Please specify the core range to which this statement applies.
Line 164: SIRM/x values appear consistent with both (titano)magnetites and maghemite in Peters and Dekker (2003), and additionally overlaps with the ranges of many other elements. Could you clarify why you only discuss it here with respect to (titano)magnetite?
Line 176 – 197: Here, I understand that you argue that Maghemite is present above the Fe-redox boundary, in addition to an elevated concentration of high coercivity minerals – based on HIRM and S-ratios. However, the basis for following interpretations comes back to the idea of distinct SIRM/x values between Magnetite and Maghemite, which, to my (admittedly limited) knowledge may be less distinct than here suggested. The Peters and Dekker paper present SIRM/x values near identical to magnetite, with substantially elevated values attributed to Hematite, Goethite, Pyrrhotite and Greigite. Thus, it is unclear to me how SIRM/x values of ~25 suggest presence of Maghemite here.
Line 177: at this depth
Line 181: First appearance of IRM as an acronym, currently defined in Line 201.
Line 191-196: I do not understand why a short period of elevated magnetic concentration at 260 – 240 mbsf precludes (or suggests negligible influence of) magnetite dissolution at the Fe-redox boundary, especially where such a marked decrease in magnetic concentration coincides with color change. Are these truly incompatible?

Rock Magnetism
Line 239: Citation of curie temperature.
Line 252: “should be caused by maghemite” could be rephrased to something like “are consistent with the inferred presence of maghemite”.
Line 255: Separate FORC discussion into a new paragraph.
Line 258: Move citation to relevant part of the sentence.
Line 259: Clarify what the central ridges of Sections A and B are being compared to.
Line 260: Please cite where interpretations of FORC are drawn from a comparison to existing literature.

TEM
As I have no significant expertise with TEM observations and interpretation, I will leave a detailed critique of this section to other reviewers. In general, I found that the section could be restructured to more clearly link observations and interpretations.
Figure 6: I find the panel layout and numbering rather confusing, and did not find the caption sufficiently comprehensive to explain the figure (as a non-expert).

XRF
Line 294: To my knowledge, rubidium is preferentially concentrated in the silt fraction of marine sediments (https://doi.org/10.1016/S0037-0738(97)00049-3), and therefore may not be the most suitable as a normalizer for productivity. Does Ba/Al, which is a more widely accepted paleo-productivity proxy, agree with Ba/Rb?

Discussion
Line 317: Remove double space following “hinterland”
Line 330 (Figure 7): I understand that you do not include section A and B samples in plots a and b due to oxidation in this interval. However, despite their relatively poor correlation to MS, it could be useful to include this data in the figure, even if they are excluded from calculation of the trendline.
Line 336 – 342: General comment – throughout this section you describe cold and warm intervals, however these are certainly periods of large climatic variance and presumably, obliquity-paced glacial cycles. Thus, when presenting IRD and diatom abundances, it may be worth clarifying whether these are associated to interglacial stages within the wider warm/cold period, or prevail throughout. It’s just a thought to consider.
Line 336 – 342: Additionally, it could be good to mention the fate of eroded material from the continent during these proposed warm intervals. I assume kaolinite is still eroded and transported to the grounding line, but for some reason is not deposited on the continental rise. So you hypothesize that it accumulates on the inner continental shelf, or is there another reason for its absence / reduction offshore?
Line 345 – 347: This could be rephrased to put focus on evidence (decreasing diatoms). Suggestion: “Based on the low relative diatom abundance, we propose that these two sections represent cold intervals where sea ice expansion limited light transmission to the upper ocean and resulted in reduced productivity.”
Line 366-369: I understand line 366 to suggest that cold periods have a low sedimentation rate, and warm periods have a high sedimentation rate. Lines 367 – 369 (and the later Lines 378 – 384, or thereabouts) seem to suggest you refer to decrease in the long-term sedimentation rate across/following these cold periods. This may be an issue with my reading, but it could be worth clarifying in my opinion.
Line 387: Is “on orbital timescale” important here?
Line 392 (Figure 8): Great Figure. A similar annotation could be added in panel (b), outlining the processes driving reduced magnetic minerals.
Line 396: The IRD acronym could be defined here using parentheses, e.g. … icebergs releasing ice-rafted debris (IRD).

Line 401-403: Add reference to U1532 to make clear this relates to the study's own conclusions.
Line 465: I agree with this sentiment, however this is the first mention of any implication for future scenarios. If you want to include this aspect, I would suggest to more directly address it in the introduction and discussion – albeit briefly. Additionally, in the introduction, you suggest that the glacial instability is currently driven by CDW influx. As your findings seem to suggest a global temperature control on the stability of the Amundsen sector (rather than a rapid and unpredictable localized forcing), does this research suggest that we may expect a more predictable response from the sector and lower likelihood of catastrophic collapse? Or does it suggest that CDW inflow is modulated by global climate? Just ideas to consider.
Author Contributions
This format is very hard to read due to the long list of initials. I would suggest to remove periods between each initial. e.g MZ and LG conceived the project... JSW, KG, CX, and LW revised… SMB, BAH, ARWH, TMK, JMF, MY, MLP, JP, ZY, YZ, DKK, and AK contributed feedback.

Supplemental Information
The order of items in the supplement is unclear. Please group relevant cyclostratigraphy discussion with its figure, and present figures mentioned in the text first.

Figure S1: This figure is not referenced in the main text.
Glacial-interglacial time-scale… :
I would suggest to either extensively rewrite this section, or question its inclusion at all. I do not see the immediate relevance to the fundamental conclusions of the manuscript, nor
You suggest that Br/Rb serves as an indicator of marine organic matter, citing Ziegler et al 2008, however to the best of my knowledge, their publication does not mention rubidium at all. Please correct this, and clearly state if Br/Rb is a newly proposed proxy or an established method. After a brief literature search, I have only found one example of this ratio being applied as a productivity proxy (Wu et al 2024, Paleo3, https://doi.org/10.1016/j.palaeo.2024.112287), located in the north Arabian Sea during the Holocene. If a new approach (in the Southern Ocean at least), it would be useful to show how this compares with other more established proxies for productivity (XRF-derived, or otherwise).
The second paragraph here reads as a list of facts, and should be restructured to lead the reader logically through the arguments.
Such comments like "the slightly decreased illite content suggests the PIG retreat[ed] during interglacial periods" do not have clear relevance to this study.
The section needs a detailed grammatical check, with a number of sentences throughout that do not make sense

Figure S8: Typo – Cyclostratig[g]raphy (and in table of contents)
Cyclostratigraphy Method: Correct the figure citations throughout to Supl. Fig. 8.

Reviewer #2

(Remarks to the Author)
Review of "Magnetic mineral properties of continental rise sediments in the Amundsen Sea reveal West Antarctic Ice Sheet dynamics since the early Pliocene" by Zhang et al.

In this manuscript, Zhang et al. use a marine sediment core, IODP Site U1532, recovered from the Amundsen Sea to reconstruct West Antarctic Ice Sheet variability spanning the last ~4.1 Ma. They use changes in magnetic minerals, and particularly the Anhysteretic remanent magnetization (ARM) as a proxy for WAIS extent in the Amundsen Sea region. Based on the low magnetic mineral concentrations, they suggest that WAIS in Amundsen Sea sector might have been completely collapsed between 4.1 – 3.8 Ma. They define four distinct cooling phases from 4.1 to MPT, and suggest that Antarctic cooling occurred in phases. They further compare their ARM/WAIS record with regional records around Antarctica, as well as with global SST and benthic d18O stacks and further suggest that WAIS variability was closely coupled with global climate.

Overall, the MS is nicely written, covers a very interesting topic and will be significant to the field -- improving our understanding of the past (warmer than present states) and future variability of WAIS. The study also presents a continuous geological record from Amundsen Sea, spanning the last 4.1 Ma, and is extremely valuable as such continuous records are scarce around Antarctica. However, I found some parts of the MS difficult to follow. I also expected the WAIS dynamics to be the main focus, yet I feel this was a bit lost within the detailed technical/method-heavy sections, and I feel some of the arguments in the discussion and SM file would also benefit from more quantitative support/justification. Data is presented poorly in some of the figures (e.g., Figs 2, 9), which makes it difficult to follow/properly assess. Even though I find the study extremely interesting and highly valuable (and think that it should eventually be published), I feel that in its current form, the MS may be more suitable for a more focused journal rather than a general scientific journal such as Nature Communications.

Main comments:

- There are some general statements regarding glacial-interglacial changes, yet some of these arguments need more quantitative support. For example, IRD and XRF element ratios are mentioned often. IRD is never shown (unless they are referring the presence of pebbles, which also needs some clarification, as this is never mentioned in methods) and some of the XRF ratios seem to be only available for two depth intervals (i.e., Br/Rb and Zr/Rb are only shown for the intervals 149-145 m and 201-197.2 m in the supplementary). If available, I think including and discussing these data further could improve

their arguments.

- The division of the sections (A-F) based on magnetic properties also need a bit more clarification/justification. By looking at Figure 2 and Table S1, it is not clear to me how these sections (A – F) were divided. Are there any significant differences between the properties? It would be good to include some info on how they made these divisions, which parameters did they focus on etc. For example, from Fig 2 or Table S1, I don't see any clear distinction between D, E and F ? I also see from Fig 2 that these sections are marked with different colors – does the color-coding here refer to anything specific, e.g., cold/warm?

- The main conclusion of the MS seems to be that the WAIS in the Amundsen sector follows global temperature changes.However, mechanisms for WAIS dynamics are never discussed.
For instance, as they mention in the intro, stronger CDW inflow has been suggested for WAIS retreat. This may be outside the scope of this MS, but wouldn't it be possible to explore these further using e.g., their existing XRF data, or models?

Below are more specific comments & minor suggestions listed per line numbers, that I hope would be helpful in improving the MS :

L41: The beginning of the first sentence "Our incomplete understanding" – reads like a double negative here. I leave it to authors but would suggest something like e.g., "our current understanding……is incomplete and limits our ability…" ?

L87-92: This could have been phrased a bit more clearly, e.g., they also do compare and show data from U1533 as well.

L94: "other proxies" is a bit vague, can you expand/list here what these are?

L98/Figure 1: There are many more sites shown in the large-scale inset map showing Antarctica (yellow circles). These should be explained/cited/referred to in the figure caption.

L103: This section reads like it could have been in the methods section ?

L105: lat-lon info for the core site should also be provided

L106: please expand: what is the max age of 5.7 Ma is based on..?

L124/Table 1: this table is very helpful, but breaks the flow a bit. Wonder if it should be in the supplementary or if it would even be more helpful to include few sentences in the main text along when these parameters are presented in results ?

L134/Figure 2: There is a lot of information presented in this figure, yet I feel the data is a bit lost because of the way it was plotted (too dense/small/narrow data plots). I think this figure would highly benefit from a better visualization. Data are plotted very small/narrow, which makes it difficult to see & compare all the variabilities & trends, there are also too many lines and small text within. Resolution is too low when zoomed-in. Figure text/caption is missing a lot of crucial details. More specific comments below:
- I only see age control points here, it was mentioned that age model was well established (L118), but as far as I can tell all data are plotted vs depth here ? Is the age model exactly the same as the published Gohl et al 2021? In that case I'd suggest plotting the data vs age. This would open up some space & help to make the actual data larger/visible. If crucial, these can be (and is already) shown in supplementary.
- The subtext below/above each parameter is helpful, but I'd suggest removing "less" and "more", as this does not provide much info. If necessary, (+) / (-) signs could be added instead of including more text within the figure.
- What do the red lower case "a" and "b" in (next to sed rate plot) represent?
- The orange rectangle marking the significant warming could have also been marked differently (There are a number of red dashed lines, so this is not very visible). And is there a reason why this rectangle is not extended into the ln(Ba/Rb) plot?
- In general: I'd also recommend choosing colorblind safe color palettes (combination of red and green lines could be an issue)
- Would also be good to clarify/add here that some of these results are compared to the shipboard data/measurements ?

L176-177: both "downcore" and "down-core" are used

L180-182: Perhaps need to reword here a bit, otherwise it reads as it should be in discussion?

L250: Please write fully instead of "etc"

L268-272/Figure 6: Would be good to refer to sub-figures a-j in figure text, with a short info.

L292-297: Not sure any results are presented here? I think this part might be better suited for the methods section?

L340: I wonder if the shipboard sediment green-blue ratio is available, and if it would provide useful here to check in comparison to diatom/productivity (e.g., see Li et al., 2022, G-cubed: https://doi.org/10.1029/2022GC010350)

L378: I thought the main focus was 0-300m, but suddenly all cores from Site 1532 (0-800 m, Fig S9) as well as data from Site 1533 (Fig S10) are introduced here. This could have been mentioned/clarified in the intro.

L381: I don't see IRD counts in Fig S9? Are pebble counts considered to be IRD ? If so please provide more details on this method. As far as I can tell this is only presence of pebbles, not counts ?

L385: Aren't the sites U1532 and U1533 too close to each other to say that these signals are not site-specific ?

L443/Figure 9: There is again too much information in the figure, and a lot of details are missing in the Figure text. Having the age in y-axis is also not very helpful, regardless, all data should be plotted in same direction (i.e., warm-cold directions seem to be reversed with the Site 1096 Opal data) . More specific comments below:
- What are the red dash lines on the ARM record ?
- Some of the data they compare to have references on the figure (e.g., IRD, d18O records), while some don't (e.g., global SST, benthid stacks). This needs to be consistent, and follow the same in-text reference style
- The figure resolution is too low, some text are almost not readable
- I assume the inset map in Figure 1 includes these other core sites & studies mentioned here, but they need to refer to the map here and provide a detailed info on these sites & comparisons.
- I also don't see any uncertainties mentioned or plotted here.
- I assume the blue dashed highlights/marks are the cooling phases they mention in text, but this should be explained here. There are also more intervals marked than what was mentioned as the 4 cooling phases ("four distinct transitions to a colder climate at 3.8 Ma, 3.2 Ma, 2.4 Ma, and 1.6 Ma") – so what do the 0.9 Ma and 4.1 Ma highlight?
- I think particularly the Global SST and the benthic stack records are plotted too narrow to fully see the data variability in comparison with the ARM record. It would have been helpful to see ARM and the global stacks plotted together.. with regards to L434-437: I am not sure I see a "great consistency" or a "close alignment" based on how the data is presented here..
- "Figure 2 Sections" is often used in the MS, but I'd suggest a different/more descriptive label for these sections.

L450-451: "magnetic minerals" used twice within the sentence – perhaps reword?

L460-462: both "the Middle Pleistocene Transition" and "MPT" are used

L499-505: Was the XRF scanning done throughout the 0-300 m interval? Please state.
ln (Ba/Rb) is shown in Figure 2. However, only the 145-149m and 197-201m intervals are shown for Br/Rb and Zr/Rb. There are some general statements in the supplementary info regarding how these element ratios change with glacial/interglacial transitions, yet it is difficult to assess if these are common features (or not), without seeing the actual full data.

L525: Hope that the data will be made available upon publication, either along with the article or through a data repository. Unless there are other valid restrictions/reasons for not making data available, this does not follow the FAIR principles.

-I don't think Figure S1 has been cited within the main MS

-There is no Fig. 4 (Fig. 5 comes after Fig. 3), yet Fig 4 is cited in Supplementary text (i.e., under glacial-interglacial time scale magnetic analysis section)

Supplementary Material:

Figure S1: What are the numbers next to red rectangles refer to (1,2,3,4?).

IRD (e.g., "gradual onset of IRD during glacial-interglacial transitions" is mentioned few times here, but it is not mentioned what this is based on. Is there a IRD % data available? Please clarify.

Figure S2: Can you add a bit more details to the figure text here to clarify. E.g., there are 2 shaded boxes. And which HIRM values / intervals do not correlate with L values? Do you refer to all points within sections A, B, C, F ?

Figure S4, S5. A short info describing the figures & a more detailed figure text are also needed here. Referring to main text and main figures in the MS makes it difficult for the reader to go back and forth..

Figure S8: I though the focus here was the 0-300 m of the core, why is data between 405.72-321.68 m presented here ..?

Figure S9 (and S10): I find using both highlights and arrows at the same time too much. I think the highlights are sufficient to show an increase/decrease and suggest removing the arrows not to make the figure too crowded. I also assume most of these are shipboard data, please also add references after each parameter.

Reviewer #3

(Remarks to the Author)
The manuscript by Zhang et al "Magnetic mineral properties of continental rise sediments in the Amundsen Sea reveal West Antarctic Ice Sheet dynamics since the early Pliocene", presents an interesting and relevant study focused on reconstructing the West Antarctic Ice Sheet (WAIS) dynamics since the middle Pliocene (~4.3 Ma) based on a sedimentary archive from the Amundsen Sea continental rise (Site U1532).

The authors use rock magnetism, transmission electron microscopy and XRF core scanner Ba/Rb ratio, alongside comparisons with kaolinite content and relative diatom abundance to determine the source of magnetic minerals and reconstruct ice sheet dynamics in the region (glacial-interglacial variability). One of the key findings is the possible collapse of the WAIS between 4.1-3.8. Additionally, the authors identify four intervals of colder climatic conditions between 4.1 Ma and the Mid-Pleistocene Transition, occurring at ~3.8, 3.2, 2.4, and 1.6 Ma.

I appreciate the effort to integrate various proxies to address an important paleoclimatic question. However, I must acknowledge that I am not familiar with the specific methodologies used in this study, so I am not in a position to critically evaluate that component of the work. That said, I would like to suggest a few points for consideration, which I believe could help strengthen the manuscript and enhance its overall clarity.

In particularly I would like to highlight a recently published study by Passchier et al. (2025) "West Antarctic ice retreat and paleoceanography in the Amundsen Sea in the warm early Pliocene", which analyses the same sedimentary archive, focusing on the early Pliocene interval (5.2–3.6 Ma). Their findings appear to contrast with some of the conclusions presented here. Specifically, provenance data from 4.1 to 3.6 Ma in that study suggest relative WAIS stability during both glacial and interglacial periods, along with indications of a generally more expanded ice sheet. Given that their study presents a refined age model, detailed sedimentary facies interpretations, and a multi-proxy provenance dataset, I believe that a more explicit comparison with their results would considerably strengthen the discussion. It may also lead the authors to reconsider or further clarify some of their interpretations and objectives of their study.

I suggest that the authors consider moving some of the data presented in Figure 2 to a supplementary figure, as the figure currently appears too busy and maybe difficult to follow by non-specialist readers. In addition, I was wondering why the intervals of high paleoproductivity, inferred from Ba/Rb ratio do not correspond to higher diatom abundance?

Version 1:

Reviewer comments:

Reviewer #1

(Remarks to the Author)
The authors have undertaken a rigorous review of the manuscript considering all earlier comments. Rebuttal is robust and supported by additional information and analyses that are sufficient to address my initial concerns. The rearrangement and refocusing of the text is most helpful in clarifying the narrative, particularly regarding the movement of highly technical and/or secondary discussions to the Supplementary Material. In line with my first review, I offer no challenges to the fundamental science within or the authors' conclusions, and find it to be a worthwhile addition to the literature. I believe the revised manuscript is well suited for publication pending minor grammatical corrections, with a non-exhaustive list of corrections presented below.

Minor comments:
Line 76 and Supplementary Fig 12: "relative diatom abundance".
Line 91: Consider moving this statement on interpretations to the start of the following paragraph near line 134, as the rest of this section only deals with result description.
Line 93: Can you please indicate the depth ranges of each section?
Line 94: Perhaps move the sentence on Supplementary Table 2 to the end of this paragraph, around line 104. As written, it places undue weight on the data table and distracts from the key descriptions that follow.
Line 123 (Figure 3): Data points overlay the text annotations.
Lines 133 – 161: This section is incredibly dense and packed with information, but personally still very difficult to read. I would encourage the authors to take every effort to simplify this section, but concede that there is a limit to what can be done while retaining the essential information.
Line 141: … indicating a significantly reduced magnetic mineral concentration in the sediments.
Line 142 – 149: The back and forth comparison of sections B and C is quite confusing for me, as it is difficult to recall both which has higher/lower values for each parameter and which we are currently talking about.
Line 214: indicates
Line 245: ARM increase resulting from
Line 247: Site U1532
Line 256: ice sheet' ?
Line 256-257: Rephrase: (suggestion) On the other hand, over the million-year timescale, the increasing glacial over-deepening of the shelf could make it difficult for the grounding line of the ice sheet to advance to the shelf edge during subsequent glacial intervals.
Line 296: Acronym for ΔSST is currently introduced in Line 303.
Supplementary Figure 11: interpretations misspelled (magnetic sconcentration)
Supplementary Figure 13: a) I don't see any black line for the 1 million year smoothed record. b) 413-kyr filtered ACC strength.

Reviewer #2

(Remarks to the Author)
Review of the revised MS by Zhang et al. entitled “Episodic West Antarctic Ice Sheet advance in the Amundsen Sea since the early Pliocene”

I would like to thank the authors for their comprehensive revision and for thoroughly addressing all my comments & concerns. I think the MS has significantly improved.
I only have few minor comments & suggestions, listed below per line numbers.

L57: Should “ASE” be shown/mentioned in the Figure 1 map/caption. Also, regarding the Fig1. figure caption: Since both U1532 and U1533 are shown in the map, perhaps the short title “U1532 Site map” could be changed/removed.

L95-105: I would suggest describing the properties and differences in these sections in full sentences, rather than listing properties & values (e.g., In Section C, xxxx are observed. instead of “Section C: …”)

L97: S-300 “values” approach 1. (also L134)

L282: planktonic foraminifera listed here should be in italic font

L301-306: I found this part to be a bit confusing. In L301-302: 4.1-3.8 Ma interval is referred to as min WAIS extent corresponding to peak warm SSTs, while in L304-305, the same interval is referred to as a “cooling trend”. Please clarify.

L308: Looking at Fig 6 (and also Supl Fig 13), I don’t see a significant change at 3.8Ma based on the ARM.. To me it seems to be relatively stable until 3.2 Ma.. Are these “key transitions” based on U1532, or other data? I think it would help to justify the timings of key transitions a bit more here?

L308: Could refer to an additional figure or change “Figs. 6” to “Fig. 6” (..also note that, in general there are too many references to Fig 6 on this page)

L357: Sentence starting with “Although” reads like missing something

Suppl. Fig. 10: What do the blue bands represent? Please provide more details in the figure text. It would also be good to include a short sentence to highlight & remind that the full record (0-800m) is presented in this figure, however the focus of this study is the upper ~0-300m ?

Suppl. Fig. 11: Would suggest using “Age” instead of “Time”. And perhaps also “Ma” instead of “ka” to be consistent throughout the paper..

Suppl. Fig. 13:
“Black line shows the 1-million-year smoothed ACC strength record” – I unfortunately could not see a black line here.
“Both gray and orange boxes mark phases of ACC decline, with gray indicating WAIS expansion and orange representing WAIS retreat. “
In general, I think it would help to have highlights/bands instead of boxes. Regardless, I found the argument here a bit confusing (also see comment above regarding key transitions), most of these intervals seem relatively stable (e.g., 3.8-3.6, 3.4-3.2, 2.1-1.7; etc) -- are the changes within these intervals significant?

Reviewer #4

(Remarks to the Author)
The authors’ study is built upon the assumption that subglacial erosion by glaciers draining into the Amundsen Sea Embayment leads to export of magnetic minerals from the ice sheet, and that these are transported across the continental shelf, before being deposited in the sampled drifts. Sediment cores sampling the drifts then enable a reconstruction of ice sheet changes. Previous studies discussed in this paper support that WAIS (even ASE continental shelf) is indeed the source of terrigenous material in the these drifts – although this is perhaps less certain than the authors suggest. In my opinion the main result of this paper (ice sheet growth from 5Ma until stabilization at about 0.9Ma) very much warrants publication and is supported by clear discussion and figures. (Although “stabilization” should more clearly acknowledge that transient collapses of WAIS might have occurred since 900 ka). Some of the more detailed discussion/interpretation is also interesting, but as I have referred to below, it is not justified by the available data and this paper would be a lot stronger without it. Particularly the references to events and thresholds. See specific comments as follows.

Finally I think the title should reflect some uncertainties here, e.g. "West Antarctic Ice Sheet advance since the early Pliocene", since the episodic nature of the advance is questionable, and it's not so clear where the sediments were sourced from.

Main comments:

One question to me is the nature of the cross-shelf transport and subsequent deposition, and dilution of glacigenic material (indicative of ice dynamics) with other material. Although this is discussed (L187) I think it would be good if a version of Fig 2 was provided where quantity X is plotted in units of flux (X/m2/yr), rather than concentration (X/kg). That would give you the

actual supply rate of glacigenic debris. It should be easily calculated from sedimentation rate and density?

Another question is the source of the sediments. The authors argue that the clay minerology supports a source region on the Amundsen Sea Embayment (ASE) continental shelf (L208), and that “Terrigenous sedimentation on the Antarctic continental rise is controlled by glacial processes that generate glaciogenic detritus on land and the continental shelf, which is then transported downslope via gravity flows—such as slumps and turbidity currents—through deep-sea channels to the continental slope and rise”. Although there are indeed troughs cut into the shelf north of Pine Island/Thwaites, the floors of these troughs still slope upwards towards the shelf break (BedMachine Antarctica). How are these sediments transported uphill except by sporadic ice advances under peak glacial conditions? Dependence on local cross-shelf ocean transport (e.g, by outwards flowing buoyant meltwater plumes) opens up many further questions regarding how even small changes in ocean circulation will bias transfer of sediments from the ASE ice stream groundling lines to the core site. I further question how this occurs specific to the core location given that there is a bathymetric rise at the edge of the shelf, between the core site and ASE (just to the right of the word ‘Drift’ in Fig 1). If the drift is mainly capturing suspended particles in eastward flowing bottom currents (L192), which I assume here is the ACC, what is the origin of these particles? Probably not the ASE, except during peak glacial conditions when the ASE grounding line is right at the shelf edge. So, yes I’m sure the record reflects ice dynamic changes somewhere in WAIS, but I would suspect over a wider region than ASE itself.

L296 / Fig 6. SST is not so directly relevant for AIS dynamics, since it’s the deep ocean temperature that drives ice loss (specifically CDW in ASE as shown by many studies). Especially in Fig 6 the Clark et al. SST compilation is nearly all from the tropics and Northern Hemisphere. Of course, over glacial/interglacial timescales then CO_2, SST and deep water temperature are well correlated. If deep ocean temperature is more insightful – maybe plot the Rohling et al (2022, Rev Geophys) reconstruction in Fig 6? Some modelling studies (Garbe et al. 2020 Nature, Golledge et al. 2021 GRL, Chandler et al. 2025 Comms Earth & Env) have suggested a deep ocean temperature threshold close to present-day is sufficient to trigger WAIS collapse, does that tie in with your reconstruction?

L307-320. Picking these three transitions at 3.8, 3.2, 2.4 is perhaps optimistic – I can’t really see these standing out in the Fig 6 ARM record, and given my discussion above I’m very much not convinced this level of interpretation is justified.

L326: 400ppm CO_2 threshold for marine ice sheets – this is an unhelpful generalization which doesn’t need propagating through the literature. The Eurasian Ice Sheet was in parts a marine ice sheet (just like Antarctica is in parts) but its disintegration started long before we reached 400 ppm CO_2. Suggest this related discussion should be removed or at least very clearly given a regional context if it’s just Antarctica.

L333, Link to ACC strength, CDW upwelling and WAIS response. Probably the ACC strength isn’t the full story here. Other factors come into play when considering heat transport to WAIS, including (i) meridional changes in core of the SH westerlies that controls location of Ekman driven CDW upwelling, (ii) CDW temperature, in turn related to NADW formation in N Atlantic; (iii) strength of slope current. So yes, interesting to plot ACC strength alongside the other records in Fig 6, but later statements (“new finding of a phase shift at 1.5Ma on 400ka eccentricity bands) is too speculative to me. The proposed relationship isn’t clear to me at all, in Figs 6 or S13. At the very least can you plot the ARM and ACC records against each other in a scatter plot and show the envelopes of the scatter are significantly different before/after 1.5Ma?

L350-366 (Summary), also the Abstract. Some conclusions here (episodic expansion and link to temperature thresholds; synchronicity with major global events) should be presented with more acknowledgement of uncertainty. Or really removed – the headline message that the U1532 record shows a gradual trend of increasing WAIS glaciation through the last 5 Ma, until 900 ka, is already a great result, and I think the deeper but more speculative interpretation isn’t warranted or necessary.

Fig 5: A “Stable large ice sheet” extending to the shelf break isn’t a good depiction of the Pleistocene Antarctic Ice Sheet, which has repeatedly retreated from the shelf edge during interglacials. Perhaps “Pleistocene glacials” would be a more accurate title?

Minor points & typos

L59 “changing at the fastest rate” needs some clarification, i.e., in terms of what metric?
L61 “Holocene records link this retreat to inflow of CDW”. Likely true that CDW was the underlying case of 1940s retreat, but the cited Holocene records don’t actually resolve this recent period. A stronger case could be made by linking recent retreat to observed CDW warming?
L155 Sentence shouldn’t start with “And”.
L182 Affection -> affect.

Version 2:

Reviewer comments:

Reviewer #4

(Remarks to the Author)
Please see the attached pdf.

Version 3:

Reviewer comments:

Reviewer #4

(Remarks to the Author)
I'd like to thank the authors for their detailed and thoughtful replies to my comments on the previous draft. I am happy that these have been resolved and don't have any further concerns.
Thanks again for your efforts!

Open Access This Peer Review File is licensed under a Creative Commons Attribution 4.0 International License, which permits use, sharing, adaptation, distribution and reproduction in any medium or format, as long as you give appropriate credit to the original author(s) and the source, provide a link to the Creative Commons license, and indicate if changes were made.
In cases where reviewers are anonymous, credit should be given to 'Anonymous Referee' and the source.
The images or other third party material in this Peer Review File are included in the article's Creative Commons license, unless indicated otherwise in a credit line to the material. If material is not included in the article's Creative Commons license and your intended use is not permitted by statutory regulation or exceeds the permitted use, you will need to obtain permission directly from the copyright holder.
To view a copy of this license, visit https://creativecommons.org/licenses/by/4.0/

Response to Reviewer's comments

Manuscript No.: NCOMMS-25-21134A

In response to the feedback on our manuscript, we have incorporated substantial revisions to enhance clarity and narrative flow. Recognizing that a document with extensive track changes can be difficult to read and may obscure the main arguments, we are submitting two versions: one with and one without tracked changes. To improve the readability and logical coherence of the paper, we have removed some descriptions that were not essential to the main content during the revision process. As a result, certain points mentioned in the detailed comments may no longer be present in the revised version. We appreciate your understanding. In our point-by-point response to the major comments, we have numbered the changes to make it easier to locate them when necessary. Before we present our responses in detail below, we'd like to provide you with a list of the most important and significant revision changes:

1. We have replotted Figure 2 by converting the depth scale to the time scale. Magnetic mineralogical, concentration-dependent, and grain-size-dependent parameters are now displayed in distinct colors for better readability. The information density of the figure has also been reduced to improve clarity.
2. The Results and Discussion sections have been consolidated. To enhance accessibility for non-specialist readers, detailed rock magnetic, supplementary experimental, and TEM results have been moved to the Supplementary Materials, with only essential interpretations retained in the main text.
3. We have added discussion on the relationship between ice-sheet change, CO_2 concentration and global temperature, and included comparisons with previously published work from Site U1532.

The following responses are provided in detail:

Reviewer #1

GENERAL COMMENT:

This manuscript builds upon a relationship between magnetic parameters and kaolinite in the IODP U1532 sediment core, from the Amundsen Sea, to interpret ice sheet dynamics in the Amundsen Sector

over the Pliocene and Pleistocene. The interpretations are rooted in a correlation between Kaolinite and magnetic properties. Prior studies have reported a kaolinite source beneath the Ice Sheet in the Amundsen hinterland. Periods with greater magnetic concentrations are interpreted to reflect cold periods where the ice sheet was grounded on the outer shelf, enhancing the delivery of continental detritus downslope, while periods with low magnetic concentration reflect open ocean deposition and interglacial conditions. The authors present evidence to suggest that the sector has undergone sustained cooling, concentrated in specific intervals at 3.8, 3.2, 2.4, and 1.6 Ma. Further, they suggest that a period of sustained warmth in the Pliocene (from 4.1 to 3.8 Ma) is a most likely candidate for substantial collapse of the ice sheet, and highlight a global temperature influence on glacial dynamics in the region. In my view, the approach is suitable and the breadth of data called upon is commendable. Conclusions are largely well supported by the data and interpretative framework which they provide.

I have not found any major issues to challenge the validity of the study. However, I frequently struggled to understand the key message of the authors, and think the manuscript as a whole could benefit from further grammatical corrections and restructuring. This is particularly so for the introduction and conclusion, where the former is of utmost importance to interest the reader and provide essential context. Secondly, the Results section currently presents a mixture of results (magnetic parameter values) and interpretations (what they suggest about the magnetic mineralogy). These could be more clearly separated in my opinion, though it may be an issue of personal preference. Overall, the results section is particularly dense and difficult to read, due in part to the sheer quantity of technical terms and acronyms. Without a coherent line of thought to guide me, I had the feeling that it was an assortment of observations lacking logical progression. I concede that this may be due to my own acuity, and could possibly be no obstacle to a more intelligent member of the community. Nonetheless, please earnestly explore how to optimally structure this passage in order to clearly, concisely, and simply convey the essential information for the study.

In summary, I find this work to be a fundamentally valuable addition to the current literature, and do not find any reason to majorly challenge its methods, interpretations or conclusions within. However, the manuscript is currently held back from its full potential by a lack of clarity or logical flow in many of its arguments. The literary proficiency should not be an unsurpassable barrier for publication as it does not undermine the rigor of science within, however an improvement would drastically benefit the accessibility of the manuscript to a wider audience. I have done my best to catalogue the (mostly minor)

issues in detail, line by line.

Finally, I apologise for any comments deriving from a clear misunderstanding of the text. Where noted, please use these as an opportunity to clarify the main text to prevent other readers from reaching similarly false conclusions.

GENERAL REPLY:

We sincerely thank you for your thorough review and positive assessment of our work's value. We are particularly grateful for the detailed suggestions regarding improving the clarity and logical flow of our manuscript. We agree completely with your observations and have undertaken a comprehensive revision to address these issues. We have completely restructured the Introduction to provide a clearer background. We have significantly streamlined the Results section by reducing its length, improving logical flow, and simplifying sentence structures to enhance readability. We have moved substantial datasets and detailed findings that are not essential to the main narrative into the Supplementary Materials. This allows the core results to be presented more clearly and concisely for the general reader, while still providing full documentation for specialists. A descriptive overview of the data has been added at the beginning of the Results and Discussion section to clearly separate mineralogical interpretations from data presentation (Line 96-104). The following is a point-by-point response to your specific comments.

COMMENT 1:

Abstract: The general structure of the abstract could be refined to make it more clear which specific investigations the authors have completed on U1532 in this study. For example, lines 44-48 state significant findings on the variable provenance to the Amundsen, however it is unclear which evidence/proxies these are drawn from this study or simply provide context.

REPLY: We have revised the Abstract (Line 40-52).

COMMENT 2:

Line 50: Grammatical suggestion: "The Anhysteretic Remanent Magnetization of sediment serves as a reliable proxy …"

REPLY: We have changed that (Line 43-44).

COMMENT 3:

Line 52: The term “climate deterioration” is unclear, especially in Antarctica where one could mistakenly attribute it to periods of ice sheet collapse. This term should be replaced to clearly identify a transition to colder climate state, or use a neutral term such as “environmental transitions”.

REPLY: We have replaced “climate deterioration” with “transitions”.

COMMENT 4:

Introduction: The introduction generally covers the essential content, though could do with some revision to better draw in the reader, and improve upon general clarity and logical flow of ideas. It could be beneficial to introduce Kaolinite since the interpretation of it is a fundamental aspect which underpins the study.

REPLY: The introduction has been restructured to first highlight the critical importance of the Amundsen Sea sector for WAIS stability (Line 55-61), then outline the current limitations in understanding its long-term behavior (Line 61-70), and finally present our study as a source of novel geological evidence to address these gaps (Line 71-81). Kaolinite is addressed in the Discussion section (Line 208-212) because its introduction is required for the interpretation of magnetic mineral sources, thus avoiding redundancy in the Introduction.

COMMENT 5:

Line 58: While factual, this introductory sentence can be improved to draw in the reader. The link between interglacial climate state and cryospheric influence on sea level rise is not immediately clear.

REPLY: We have deleted that sentence.

COMMENT 6:

Line 60: The absolute level of stability of the WAIS, and required forcing to trigger true instability, remain somewhat disputed. Thus, “unstable” could be rephrased as “more sensitive” or similar, as this is less absolute.

REPLY: We have changed “unstable” to “sensitive” (Line 55).

COMMENT 7:

Line 67-68: It is unclear whether “.. and their retreat rate can be even higher than is observed today”

refers to future projections or past observations. Please rephrase to clarify that this this refers to early observations.

REPLY: This sentence has been deleted during the restructuring of the introduction.

COMMENT 8:

Line 72: “the WAIS history” is redundant.

REPLY: We have deleted it.

COMMENT 9:

Line 75-79: I suggest to split this sentence into a few. The first phrase on 14C may also be improved for clarity, eg. “^{14}C ages in sediment cores from the inner ASE shelf indicated that grounding line of the WAIS had retreated [from the outer shelf?] by the start of the Holocene”

REPLY: These sentences were omitted during the restructuring of the introduction because the focus of this paper is not on glacial-interglacial timescales, whereas these studies pertain to such timescales and would not be particularly relevant here.

COMMENT 10:

Line 81: Suggestion: “Cores obtained by the sea floor drill rig…”

REPLY: We have changed that.

COMMENT 11:

Line 82: of [the] West Antarctica

REPLY: We have deleted it.

COMMENT 12:

Figure 1: Great figure. Please add mention of the bathymetric contour intervals, or label them. The bathymetry scale could be cropped to ~5000 m. Are the contours on the continent representing bed morphology, ice morphology, or both? The context map labels a suite of other ODP/IODP cores around the continent—are these mentioned in the text? The core location icons could be slightly more prominent, but not essential.

REPLY: We have labeled bathymetric contour values. The contours on the continent represent both bed morphology and ice morphology, we have labeled and distinguished them. We have changed the color and size of the core location icons to make them more prominent. Other ODP/IODP cores around the continent on the context map was used in discussion. However, in accordance with the feedback received, we have refocused the discussion on the mechanisms of ice-sheet change and removed the section on circum-Antarctic comparisons. As a result, these sites have also been omitted from the context map.

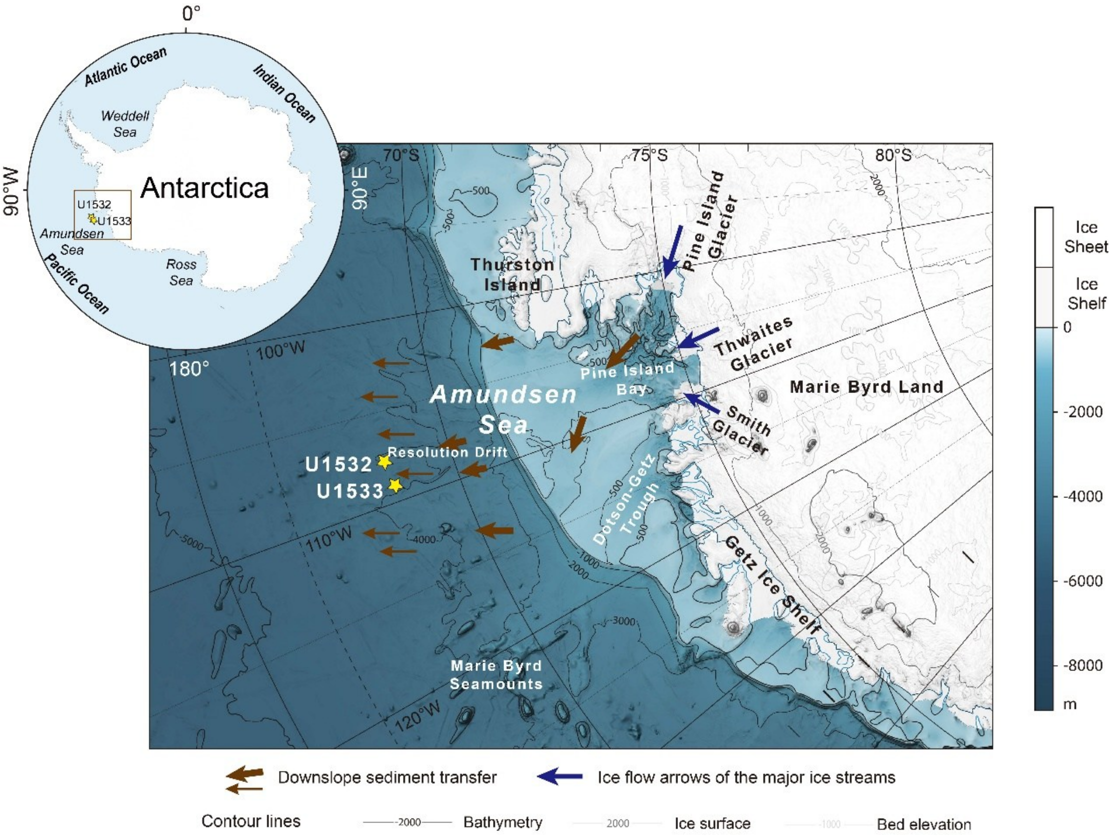

Fig. 1 | U1532 site map. *Resolution Drift in the Amundsen Sea sector and the drill sites (U1532 and U1533, yellow stars) of the International Ocean Discovery Program (IODP) Expedition 379. Bathymetry is from International Bathymetric Chart of the Southern Ocean (IBCSO) v2[19]. The downslope sediment transfer arrows, following ref. [20], is determined from the morphology on the continental slope off Pine Island Bay.*

COMMENT 13:

Geological Setting & Analyses

Line 104: Keep consistency between ca. and ~. Grammatical suggestion: Continental rise Site U1532 is located on the continental rise at the crest of the elongated Resolution Drift, ca. 270 km north of the ASE

shelf edge.

REPLY: We have standardized the use of “~” to denote “approximately.” We have corrected the grammar. And this paragraph has been moved into the Method section as the “Drill site description” (Line 369), in accordance with COMMENT 5 of Reviewer 2.

COMMENT 14:

Line 105: Clarify that this refers to a sediment coring depth of 794 m below the sea floor.

REPLY: We have clarified that (Line 382-383).

COMMENT 15:

Line 106: Please reference Gohl et al 2021 (ref # 24) for core-bottom age.

REPLY: We have added the reference (Line 383).

COMMENT 16:

Line 107: … gravity driven processes [such as] slumps and turbidity currents … This section could benefit by further addressing various sediment delivery pathways from the continent to the continental rise, as this will be essential for later conclusions. At least, please elaborate on turbidity currents and their specific origin. Can they be meltwater derived / tidally eroded sediments, or only originate via slope failure in the ASE?

REPLY: We have added a section in the Discussion on the source of sediments on the Amundsen Sea continental rise (Line 188-198).

COMMENT 17:

Line 113-14: This is methodological. I would suggest either to remove the sentence, or move it to the end of the paragraph where it follows the age model, and leads to the following results section.

REPLY: We have moved that sentence to the beginning of “Rock magnetism” in Methods section (Line 394-395).

COMMENT 18:

Line 115-118: This can be gently reworked, grouping separate ideas into smaller sentences for easier

reading.

REPLY: We have separated that sentence into a few. And this has been moved into the Method section as the “Drill site description” (Line 385-389).

COMMENT 19:

Methods

Paleomagnetism and XRF techniques are appropriate and sufficiently well documented in my opinion. As I have no experience with TEM measurement or analyses, I will leave any critique of this method to other more qualified reviewers.

Line 469: Kappabridge. I’m unsure whether “Magnetic Susceptibility System” is required here.

REPLY: We have deleted “Magnetic Susceptibility System” after Kappabridge.

COMMENT 20:

Line 477: Reference Liu et al. 2007 (G3) for L-ratio (ref # 34).

REPLY: We have added the reference (Line 404).

COMMENT 21:

Line 504-505: Elements are not proper nouns, and so do not need to be capitalized.

REPLY: Corrected (Line 438-439).

COMMENT 22:

Line 514: Precipitation? Do you mean settling - or am I missing something?

REPLY: We have corrected it to “settling” (Line 448).

COMMENT 23:

Results: Environmental Magnetism

Table 1: Please ensure every sentence containing an established interpretation is cited, even those most basic claims (such as the first sentences of SIRM and L-Ratio).

REPLY: We have added the references. According to Reviewer 2's comments, this table has been removed to the Supplementary Material as “Supplementary Table 1: Magnetic parameters and their

interpretation”.

COMMENT 24:

L-ratio: Variable L-ratios rather correspond to changing coercivity of Hematite, which is itself associated with stoichiometry, density and/or grainsize (but not solely grainsize; Liu et al 2007, G3). It can thus indicate changes in Hematite provenance. Please clarify these additional possible controls on L-ratio in this interpretation.

REPLY: We have refined the interpretation of L-ratio in Supplementary Table 1.

COMMENT 25:

Volumetric MS and Mass MS: For non-experts in the field, please clarify that grain size refers to magnetic mineral grain size, not whole sediment grainsize. (I later note that this is somewhat addressed in Line 126, but could possibly be restated - if you agree).

REPLY: We have clarified that grain size refers to magnetic mineral grain size in Supplementary Table 1.

COMMENT 26:

SIRM/χ: I would suggest to rephrase “useful for assessing mineralogy” to something like “Indicator for magnetic mineralogy”. There is greater complexity in SIRM/χ beyond the influences of (titano)magnetite and maghemite, with an additional effect of grainsize for (titano)magnetite (c.f. Figure 3 from Peters and Dekkers, 2003). Additionally, in this plot, Maghemite overlaps with the range of (titano)magnetite at ~10 mAm-1. Please carefully recheck this section.

REPLY: We have changed the interpretation of SIRM/χ in Supplementary Table 1 and we have noticed that the SIRM/χ value of maghemite is very similar to that of magnetite. Thus, we selected six samples for Lowrie's three-axis thermal demagnetization experiment. Detailed explanations of the experimental results and images are provided in the Supplementary Material. The results indicate that hematite caused the increase in SIRM/χ (Supplementary Text 4 and Supplementary Fig. 5).

COMMENT 27:

Line 133 (Figure 2):

Greenish layers (reflecting interglacial ‘periods’).

“Diatom Relative Abundance” or “Relative Diatom Abundance”.

SIRM/χ (kA m -1)—also see prior comment that Maghemite is also ~10 kA m-1.

From 100-290 m, the “greenish layers” are relatively clear in a* color reflectance, but less-so in the upper 100 m. I assumed (based on line 115-117) that these “greenish layers” were obtained from the Gohl expedition report, but while mentioned there I do not find a detailed catalogue of their occurrences like you present here. Please clarify the criteria for their classification and include them in the figure caption.

Red annotations “a” and “b” are not included in the figure caption.

REPLY: Greenish layers and diatom abundance have been deleted from Figure 2. Relative Diatom Abundance was moved to Supplementary Figure 12. The interpretation of SIRM/χ has been changed in Supplementary Table 1. Red annotations “a” and “b” were deleted along with the glacial-interglacial changes in supplementary material.

The greenish layers were originally marked based on a combination of low values for color reflectance a* and high values for ARM/SIRM (indicating finer-grained magnetic minerals), as we observed consistency between these two parameters. Above 100 meters, the consistency between the greenish layers we identified and the low values of a* weakens, which may be due to two reasons: first, the decrease in sedimentation rate reduces data resolution; second, the color change caused by oxidation in the uppermost 40 meters core makes the greenish layers difficult to distinguish. Since the data in this paper is insufficient to support a glacial-interglacial scale interpretation, and the focus is not on the glacial-interglacial scale, we have removed the relevant content. Future work will conduct detailed glacial-interglacial scale studies.

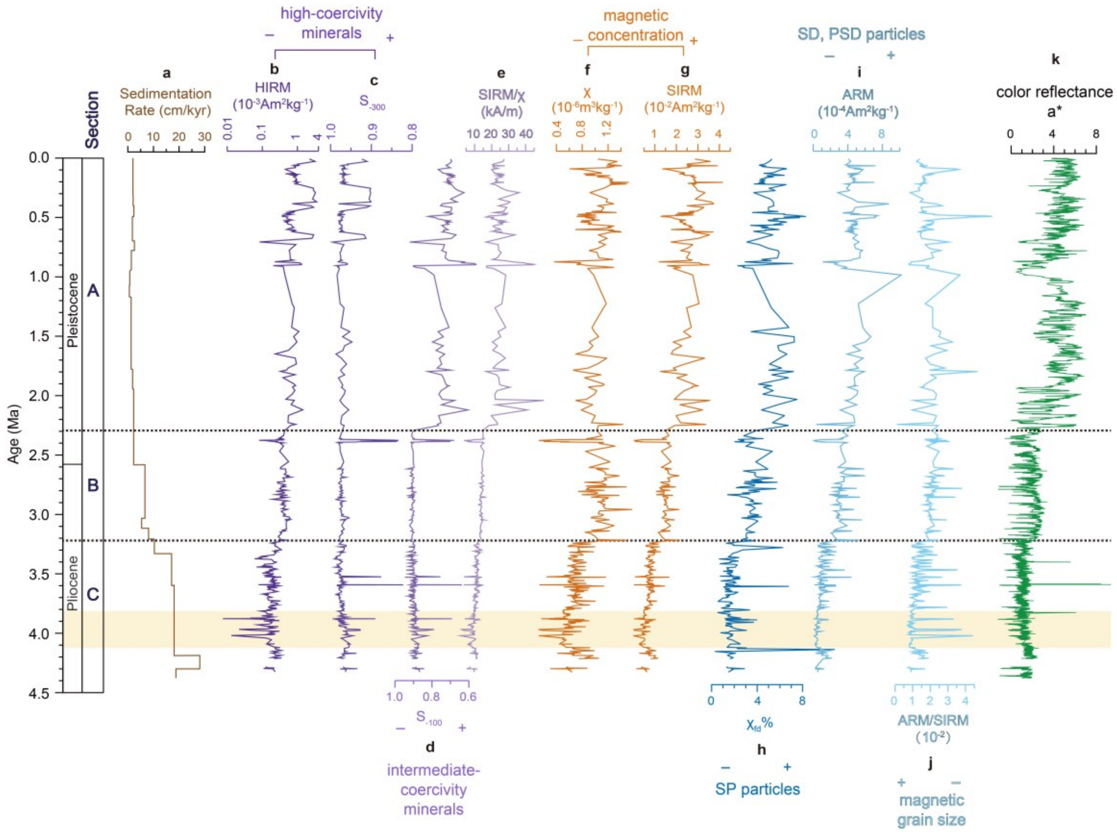

Fig. 2 | Variation of environmental magnetic parameters since ~4.33 Ma (the upper 300 m of the core) at Site U1532. *(a) Sedimentation rates. Magnetic mineralogy indicators (b) HIRM, (c) S_{-300}, (d) S_{-100} and (e) SIRM/χ are plotted in purple lines; magnetic concentration indicators (f) χ and (g) SIRM are plotted in orange lines; magnetic grain size indicators (h) χ_{fd}%, (i) ARM, and (j) ARM/SIRM are plotted in blue lines. (k) Shipboard color reflectance a*[16] are plotted for comparison. Sections A-C are separated by dashed lines: A (39.01-0 m, 2.29-0 Ma), B (88.02-39.01 m, 3.21-2.29 Ma) and C (291.43-88.02 m, 4.33-3.21 Ma). Higher HIRM and lower S_{-300} indicate more high-coercivity minerals; lower S_{-100} indicate more intermediate-coercivity minerals; variable SIRM/χ indicates changes in mineral composition; higher χ and SIRM for higher magnetic concentrations; higher χ_{fd}% and ARM for more SP and SD/PSD particles; higher ARM/SIRM for smaller magnetic grain size. The light-yellow shaded area (~4.12-3.82 Ma) marks a period with extremely low concentrations of magnetic minerals, indicating a period of extreme warmth. SP: superparamagnetic; SD: single domain; PSD: pseudo single domain. See Supplementary Table 3 for age-depth model.*

COMMENT 28:

Line 153-155: L-ratio is stable throughout Section A and B, thus HIRM is indicative changing concentrations in this interval. But sections C-F have more variable L-ratios as illustrated in Figure S2. Please specify the core range to which this statement applies.

REPLY: In the previous version of the manuscript, several points with extremely high HIRM values in uppermost core were excluded from the L-ratio assessment. Based on the current core sectioning, since section A has undergone a change in mineral composition, we conducted L-ratio assessment on section

A and sections B and C separately and found that the L-ratio is stable, respectively. Therefore, in this study, HIRM is influenced by the concentration of high-coercivity minerals (Line 134-135). The L-ratio validation has been moved to the supplementary materials (See Supplementary Text 1 and Supplementary Fig. 1).

COMMENT 29:

Line 176-197: Here, I understand that you argue that Maghemite is present above the Fe-redox boundary, in addition to an elevated concentration of high coercivity minerals—based on HIRM and S-ratios. However, the basis for following interpretations comes back to the idea of distinct SIRM/x values between Magnetite and Maghemite, which, to my (admittedly limited) knowledge may be less distinct than here suggested. The Peters and Dekker paper present SIRM/x values near identical to magnetite, with substantially elevated values attributed to Hematite, Goethite, Pyrrhotite and Greigite. Thus, it is unclear to me how SIRM/χ values of ~25 suggest presence of Maghemite here.

REPLY: We have deleted that statement. And Lowrie's three-axis thermal demagnetization experiment results indicate that hematite caused the increase in SIRM/χ (Reply of above COMMENT 26).

COMMENT 30:

Line 177: at this depth

REPLY: We have deleted that.

COMMENT 31:

Line 181: First appearance of IRM as an acronym, currently defined in Line 201.

REPLY: We have corrected that.

COMMENT 32:

Line 191-196: I do not understand why a short period of elevated magnetic concentration at 260-240 mbsf precludes (or suggests negligible influence of) magnetite dissolution at the Fe-redox boundary, especially where such a marked decrease in magnetic concentration coincides with color change. Are these truly incompatible?

REPLY: We have included a paragraph of discussion and several figures in the supplementary materials to demonstrate that dissolution has a negligible effect on magnetic parameters (Line 181-185; Supplementary Text 7, Supplementary Figs. 10 and 11, and Fig. 4).

COMMENT 33:

Rock Magnetism

Line 239: Citation of curie temperature.

REPLY: We have added the reference (Line 171).

COMMENT 34:

Line 252: "should be caused by maghemite" could be rephrased to something like "are consistent with the inferred presence of maghemite".

REPLY: We have rephrased this sentence.

COMMENT 35:

Line 255: Separate FORC discussion into a new paragraph.

REPLY: We have separated FORC discussion into a new paragraph, and it has been moved to Supplementary Material in Supplementary Text S2.

COMMENT 36:

Line 258: Move citation to relevant part of the sentence.

REPLY: Corrected.

COMMENT 37:

Line 259: Clarify what the central ridges of Sections A and B are being compared to.

REPLY: That sentence has been deleted due to changes in the sectioning.

COMMENT 38:

Line 260: Please cite where interpretations of FORC are drawn from a comparison to existing literature.

REPLY: We have added the references, and it has been moved to Supplementary Material in

Supplementary Text S2.

COMMENT 39:

TEM: As I have no significant expertise with TEM observations and interpretation, I will leave a detailed critique of this section to other reviewers. In general, I found that the section could be restructured to more clearly link observations and interpretations.

Figure 6: I find the panel layout and numbering rather confusing, and did not find the caption sufficiently comprehensive to explain the figure (as a non-expert).

REPLY: We have revised the TEM image numbers and captions. And this paragraph has been reorganized for clarity. To focus the main text more extensively on WAIS dynamics, we have placed TEM-related content in the Supplementary Materials (Supplementary Text 5 and Supplementary Fig. 7).

COMMENT 40:

XRF: Line 294: To my knowledge, rubidium is preferentially concentrated in the silt fraction of marine sediments (https://doi.org/10.1016/S0037-0738(97)00049-3), and therefore may not be the most suitable as a normalizer for productivity. Does Ba/Al, which is a more widely accepted paleo-productivity proxy, agree with Ba/Rb?

REPLY: The Ba/Rb and Ba/Al peaks (interglacial periods) correspond well (following figure). But we have deleted the Ba/Rb ratios along with the glacial-interglacial-related content.

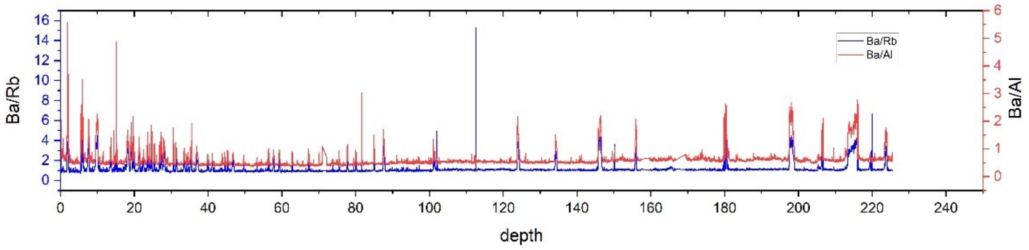

COMMENT 41:

Discussion: Line 317: Remove double space following “hinterland”.

REPLY: Removed.

COMMENT 42:

Line 330 (Figure 7): I understand that you do not include section A and B samples in plots a and b due to oxidation in this interval. However, despite their relatively poor correlation to MS, it could be useful to include this data in the figure, even if they are excluded from calculation of the trendline.

REPLY: We have included section A and B samples in this figure. This Figure has been removed to Supplementary Material as Supplementary Figure 8.

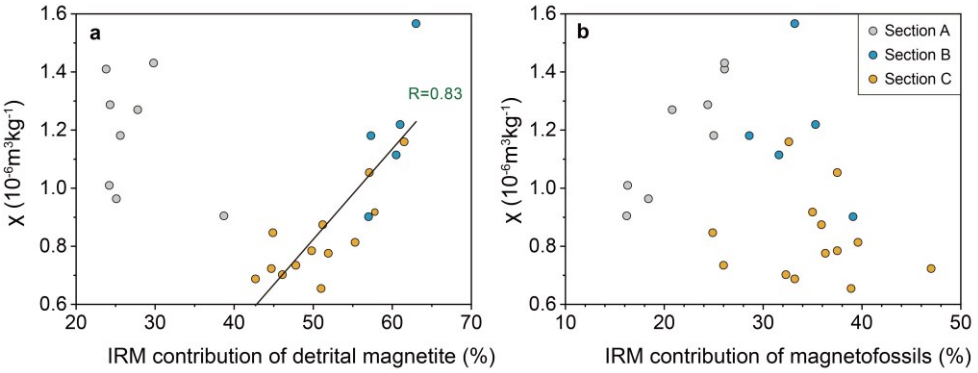

Supplementary Figure 8. Correlation of detrital magnetic minerals input and magnetofossils with total magnetic mineral concentration. *(a) biplot of IRM contribution of detrital magnetite vs. χ; (b) IRM contribution of magnetofossils vs. χ. R: Pearson' s correlation coefficient. The points of samples in section A did not participate in the correlation calculation in this figure, because section A was affected by oxidation. Samples in section A had an additional main component H in the IRM decomposition.*

COMMENT 43:

Line 336-342: General comment—throughout this section you describe cold and warm intervals, however these are certainly periods of large climatic variance and presumably, obliquity-paced glacial cycles. Thus, when presenting IRD and diatom abundances, it may be worth clarifying whether these are associated to interglacial stages within the wider warm/cold period, or prevail throughout. It's just a thought to consider.

REPLY: In the figure below, the green bars denote intervals of low a* values, which correspond to interglacial periods. A strong correspondence between these intervals and high diatom abundance can be observed, confirming that interglacials are consistently associated with elevated diatom productivity. However, even in intervals of Section C not marked by green bars, diatom abundance remains higher than that observed throughout Section B. We therefore conclude that the overall increase in diatom abundance in Section C reflects a prolonged warm phase operating on a longer timescale.

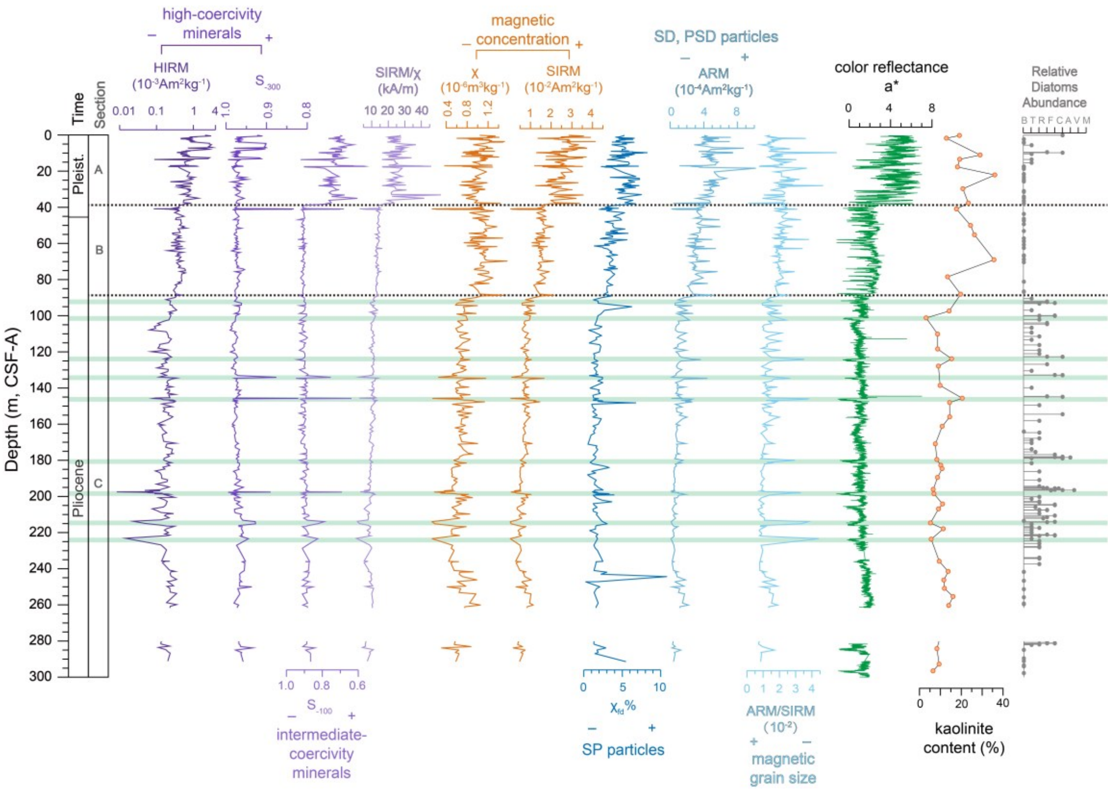

Furthermore, the coherent long-term trend between the newly presented Si/Al ratio and relative diatom abundance in section C indicates a prolonged warm period.

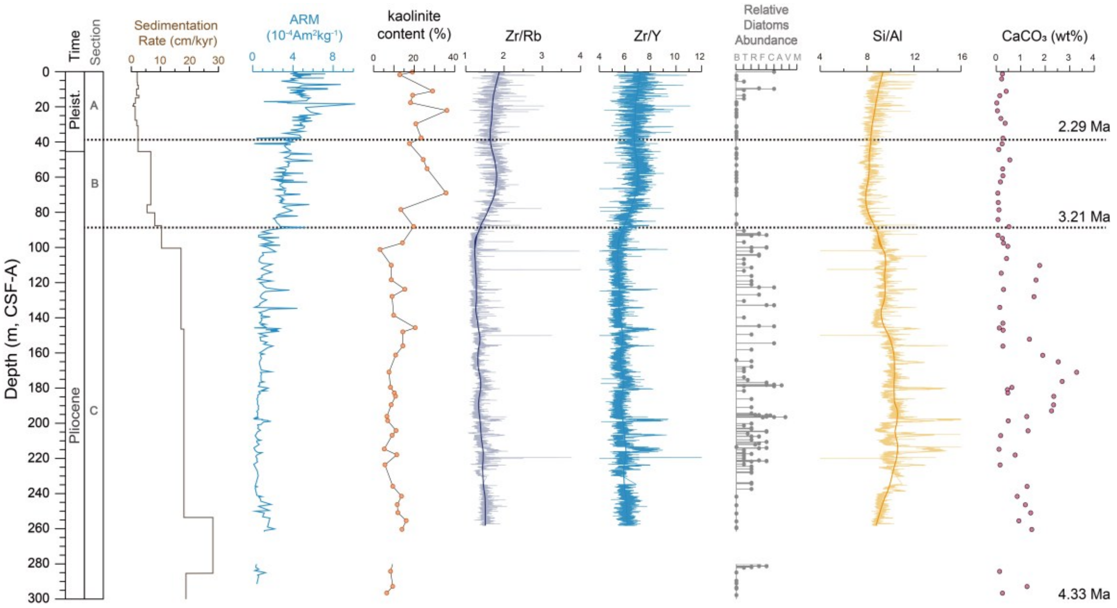

Supplementary Figure 12. Sedimentation rate, ARM, kaolinite content[16], Zr/Rb, Zr/Y, relative diatoms abundance[16], Si/Al, and $CaCO_3$ content[16] of the 0-300 m core at Site U1532.

COMMENT 44:

Line 336-342: Additionally, it could be good to mention the fate of eroded material from the continent during these proposed warm intervals. I assume kaolinite is still eroded and transported to the grounding line, but for some reason is not deposited on the continental rise. So, you hypothesize that it accumulates on the inner continental shelf, or is there another reason for its absence / reduction offshore?

REPLY: We have added a paragraph to the discussion regarding the sources of terrigenous sediments (including kaolinite; Line 188-198). Less kaolinite-bearing rock was eroded during warm periods in the first place, indicating that it was not deposited elsewhere. This has been discussed in Hillenbrand et al., 2009 (Quaternary Science Reviews, doi:10.1016/j.quascirev.2008.12.010) and Passchier et al., 2024 (Nature communications, https://doi.org/10.1038/s41467-025-60772-8). The kaolinite content derived from XRD analysis reflects its relative proportion in the clay mineral assemblage. As such, its variation may be affected by changes in the concentrations of other clay minerals.

COMMENT 45:

Line 345-347: This could be rephrased to put focus on evidence (decreasing diatoms).

Suggestion: "Based on the low relative diatom abundance, we propose that these two sections represent cold intervals where sea ice expansion limited light transmission to the upper ocean and resulted in reduced productivity."

REPLY: We have rephrased this sentence (Line 239-245).

COMMENT 46:

Line 366-369: I understand line 366 to suggest that cold periods have a low sedimentation rate, and warm periods have a high sedimentation rate. Lines 367 – 369 (and the later Lines 378-384, or thereabouts) seem to suggest you refer to decrease in the long-term sedimentation rate across/following these cold periods. This may be an issue with my reading, but it could be worth clarifying in my opinion.

REPLY: In the revised manuscript, we focus on the 0-300 m core section (since ~4.33 Ma) and find that sedimentation rates decreased progressively with WAIS advance. Accordingly, we have modified the interpretation of the sedimentation rate decline (Line 252-263).

COMMENT 47:

Line 387: Is "on orbital timescale" important here?

REPLY: We have deleted “orbital timescale” related text.

COMMENT 48:

Line 392 (Figure 8): Great Figure. A similar annotation could be added in panel (b), outlining the processes driving reduced magnetic minerals.

REPLY: We have revised the Depositional pattern diagrams according to revised text (Line 224-251).

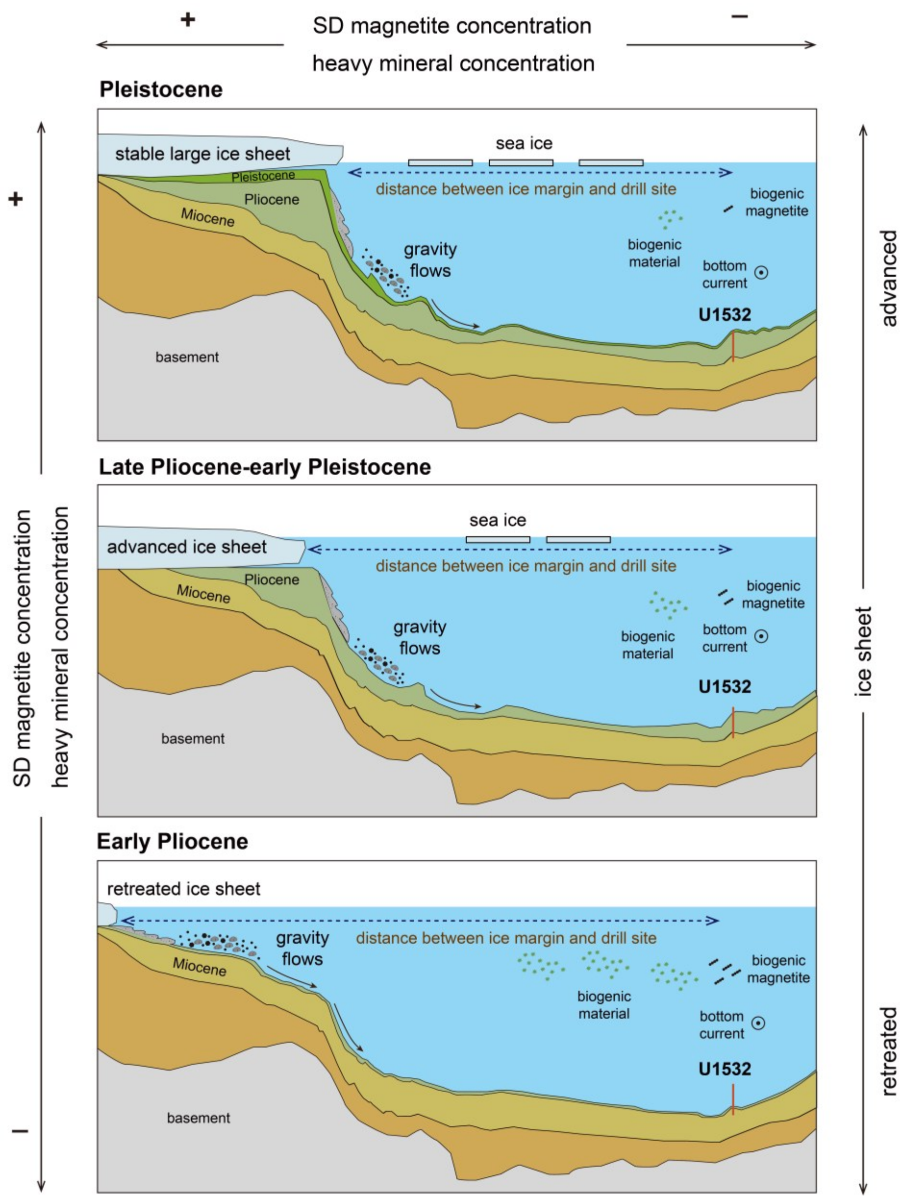

Fig. 5 | Depositional pattern diagrams at Site U1532. *Early Pliocene: a retreated ice sheet limited subglacial erosion, resulting in a low supply of terrigenous detritus from the Amundsen Sea Embayment (ASE) shelf and its*

hinterland. Combined with long transport distances to the continental rise, this led to low concentrations of magnetic minerals at the drill site. Open-ocean conditions prevailed, favoring diatom productivity. Late Pliocene-early Pleistocene: glacial advance promoted progradation of the outer shelf and shelf growth in the ASE[33]*. Enhanced bedrock erosion and shortened transport distances significantly increased the supply of heavy mineral and magnetite to the rise. Pleistocene stable large ice sheet: the ice sheet remained relatively stable at a largely expanded configuration, comparable to its early Pliocene extent. Outline of basement and sediment layers referenced to crossing seismic lines*[33] *in area of Site U1532.*

COMMENT 49:

Line 396: The IRD acronym could be defined here using parentheses, e.g. … icebergs releasing ice-rafted debris (IRD).

REPLY: We have removed all references to IRD, as Reviewer 2 correctly noted that what we initially identified as IRD corresponds to the shipboard category of "pebbles". Moreover, the inclusion or exclusion of IRD-related interpretations has no bearing on the conclusions derived from rock magnetic proxies.

COMMENT 50:

Line 401-403: Add reference to U1532 to make clear this relates to the study's own conclusions.

REPLY: We have clearly stated that the ARM record from Site U1532 represents ice-sheet advances/retreats (Line 250-251).

COMMENT 51:

Line 465: I agree with this sentiment; however, this is the first mention of any implication for future scenarios. If you want to include this aspect, I would suggest to more directly address it in the introduction and discussion—albeit briefly. Additionally, in the introduction, you suggest that the glacial instability is currently driven by CDW influx. As your findings seem to suggest a global temperature control on the stability of the Amundsen sector (rather than a rapid and unpredictable localized forcing), does this research suggest that we may expect a more predictable response from the sector and lower likelihood of catastrophic collapse? Or does it suggest that CDW inflow is modulated by global climate? Just ideas to consider.

REPLY: The Introduction now includes description on the uncertainties associated with future projections of the WAIS (Line 67-70). Furthermore, the introduction states that since the Holocene,

Circumpolar Deep Water (CDW) has been identified as the primary driver of West Antarctic Ice Sheet (WAIS) melt, while carbon dioxide (CO_2) plays a critical role in its long-term destabilization (Line 61-63). And the Discussion have been extensively revised to emphasize mechanistic interpretation. Our evidence supports a long-term coupling between WAIS expansion and declines in CO_2/temperature (Line 307-310; Line 321-332), whereas the impacts of CDW and the ACC are likely non-linear (Line 333-349).

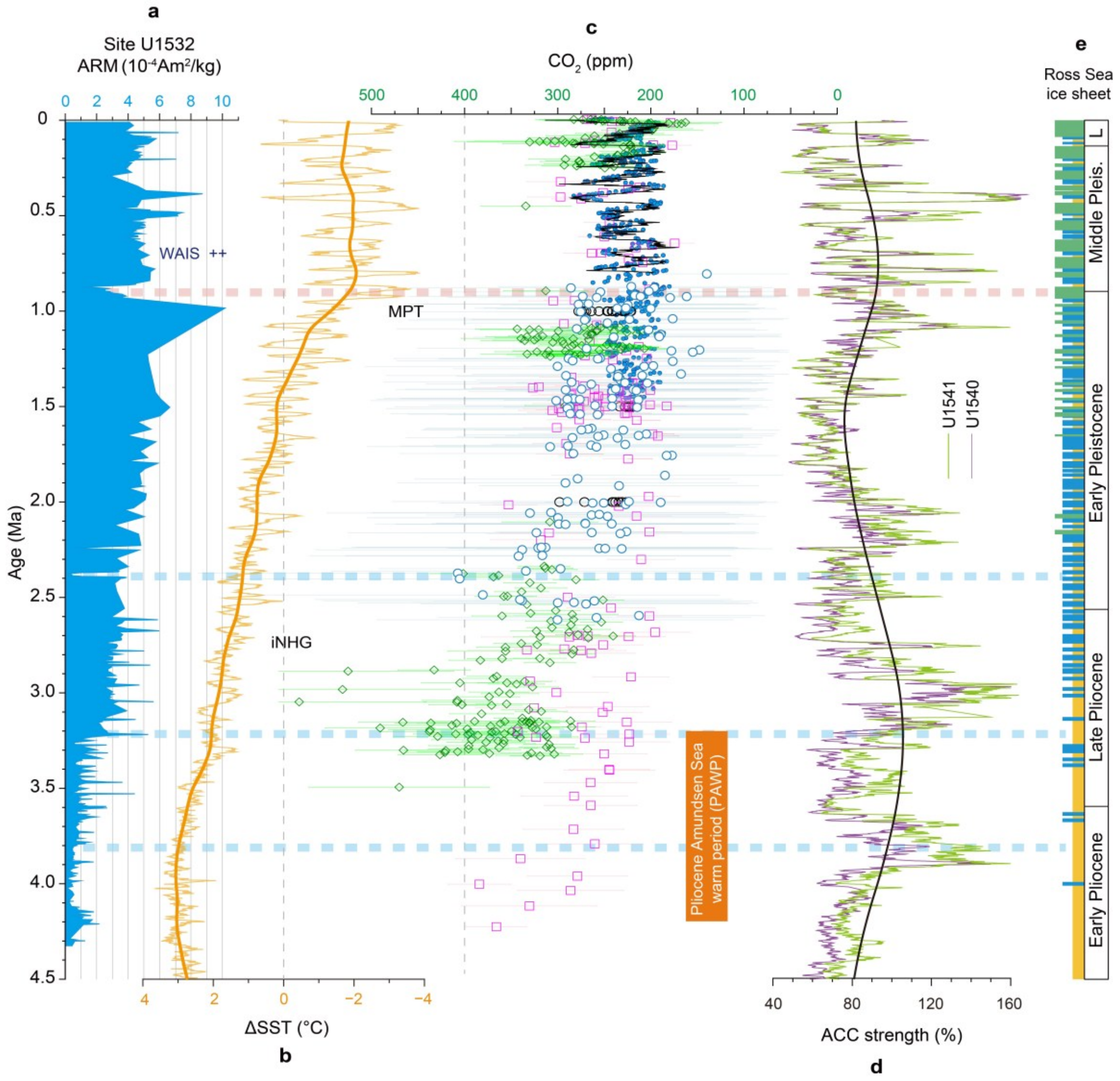

Fig. 6 | WAIS evolution since ~4.33 Ma recorded by ARM at Site U1532. *(a) ARM values of Site U1532, used as indicator for ice sheet advance/retreat. (b) Global sea surface temperature change (ΔSST)*[48] *and its LOWESS smoothed long-term trend; grey dashed line: preindustrial level*[48]*. (c) Proxy and ice-core CO_2 data. Magenta and green symbols are harmonized $\delta^{11}B$ values on planktonic foraminifera T. sacculifer and G. ruber, respectively*[49]*. Blue hollow circles are based on measurements on paleosols from the Chinese Loess Plateau*[50]*. Blue dots are from $\delta^{13}C$ of leaf wax*[51]*. Black lines are ice-core CO_2 record over the past 800 ka*[52] *and black hollow circles are early Pleistocene blue-ice CO_2 snapshots*[53]*. (d) Relative Antarctic Circumpolar Current (ACC) strength variations at Site U1540 and Site U1541*[54]*. Black line shows the 1-million-year smoothed ACC strength record*[54]*. (e) Modeled sediment*

facies in the Ross Sea, close to AND-1B (yellow = open ocean; blue = floating ice; green = grounded ice)[2]*. Yellow and blue/green here correspond to the AND 1B diatomite (yellow) and diamictite (green) intervals*[55]*. Pliocene Amundsen Sea Warm Period (PAWP): based on the seismic correlation of sediment layers from the drill sites to the continental shelf, grounding zone wedges have been identified in the shelf sediments that predominantly formed during ice sheet retreat within an extended Pliocene warm period from 4.2-3.2 Ma*[33]*. MPT: Mid-Pleistocene Transition. iNHG: the intensification of the Northern Hemisphere glaciation. Blue dashed lines mark three transition points to a larger WAIS at ~3.8, 3.2 and 2.4 Ma. Pink dashed line marks a transition point to a relatively stable ice sheet at ~0.9 Ma.*

COMMENT 52:

Author Contributions

This format is very hard to read due to the long list of initials. I would suggest to remove periods between each initial. e.g MZ and LG conceived the project... JSW, KG, CX, and LW revised… SMB, BAH, ARWH, TMK, JMF, MY, MLP, JP, ZY, YZ, DKK, and AK contributed feedback.

REPLY: We have removed periods between each initial.

COMMENT 53:

Supplemental Information

The order of items in the supplement is unclear. Please group relevant cyclostratigraphy discussion with its figure, and present figures mentioned in the text first.

REPLY: We have reorganized the order of items in the Supplementary Material. We have deleted cyclostratigraphy-related content because we have focused on 0-300 m of the core.

COMMENT 54:

Figure S1: This figure is not referenced in the main text.

Glacial-interglacial time-scale… :

I would suggest to either extensively rewrite this section, or question its inclusion at all. I do not see the immediate relevance to the fundamental conclusions of the manuscript, nor You suggest that Br/Rb serves as an indicator of marine organic matter, citing Ziegler et al 2008, however to the best of my knowledge, their publication does not mention rubidium at all. Please correct this, and clearly state if Br/Rb is a newly proposed proxy or an established method. After a brief literature search, I have only found one example of this ratio being applied as a productivity proxy (Wu et al 2024, Paleo3,

https://doi.org/10.1016/j.palaeo.2024.112287), located in the north Arabian Sea during the Holocene. If a new approach (in the Southern Ocean at least), it would be useful to show how this compares with other more established proxies for productivity (XRF-derived, or otherwise).

The second paragraph here reads as a list of facts, and should be restructured to lead the reader logically through the arguments. Such comments like “the slightly decreased illite content suggests the PIG retreat[ed] during interglacial periods” do not have clear relevance to this study.

The section needs a detailed grammatical check, with a number of sentences throughout that do not make sense.

REPLY: The glacial-interglacial statement and XRF data description has been deleted due to the small relevance with main text.

COMMENT 55:

Figure S8: Typo—Cyclostratig[g]raphy (and in table of contents).

Cyclostratigraphy Method: Correct the figure citations throughout to Supl. Fig. 8.

REPLY: This part has been deleted.

==

Reviewer #2

GENERAL COMMENT:

In this manuscript, Zhang et al. use a marine sediment core, IODP Site U1532, recovered from the Amundsen Sea to reconstruct West Antarctic Ice Sheet variability spanning the last ~4.1 Ma. They use changes in magnetic minerals, and particularly the Anhysteretic remanent magnetization (ARM) as a proxy for WAIS extent in the Amundsen Sea region. Based on the low magnetic mineral concentrations, they suggest that WAIS in Amundsen Sea sector might have been completely collapsed between 4.1 – 3.8 Ma. They define four distinct cooling phases from 4.1 to MPT, and suggest that Antarctic cooling occurred in phases. They further compare their ARM/WAIS record with regional records around Antarctica, as well as with global SST and benthic d18O stacks and further suggest that WAIS variability was closely coupled with global climate.

Overall, the MS is nicely written, covers a very interesting topic and will be significant to the field --

improving our understanding of the past (warmer than present states) and future variability of WAIS. The study also presents a continuous geological record from Amundsen Sea, spanning the last 4.1 Ma, and is extremely valuable as such continuous records are scarce around Antarctica. However, I found some parts of the MS difficult to follow. I also expected the WAIS dynamics to be the main focus, yet I feel this was a bit lost within the detailed technical/method-heavy sections, and I feel some of the arguments in the discussion and SM file would also benefit from more quantitative support/justification. Data is presented poorly in some of the figures (e.g., Figs 2, 9), which makes it difficult to follow/properly assess. Even though I find the study extremely interesting and highly valuable (and think that it should eventually be published), I feel that in its current form, the MS may be more suitable for a more focused journal rather than a general scientific journal such as Nature Communications.

Main comments:

- There are some general statements regarding glacial-interglacial changes, yet some of these arguments need more quantitative support. For example, IRD and XRF element ratios are mentioned often. IRD is never shown (unless they are referring the presence of pebbles, which also needs some clarification, as this is never mentioned in methods) and some of the XRF ratios seem to be only available for two depth intervals (i.e., Br/Rb and Zr/Rb are only shown for the intervals 149-145 m and 201-197.2 m in the supplementary). If available, I think including and discussing these data further could improve their arguments.

- The division of the sections (A-F) based on magnetic properties also need a bit more clarification/justification. By looking at Figure 2 and Table S1, it is not clear to me how these sections (A – F) were divided. Are there any significant differences between the properties? It would be good to include some info on how they made these divisions, which parameters did they focus on etc. For example, from Fig 2 or Table S1, I don't see any clear distinction between D, E and F ? I also see from Fig 2 that these sections are marked with different colors – does the color-coding here refer to anything specific, e.g., cold/warm?

- The main conclusion of the MS seems to be that the WAIS in the Amundsen sector follows global temperature changes. However, mechanisms for WAIS dynamics are never discussed.

For instance, as they mention in the intro, stronger CDW inflow has been suggested for WAIS retreat. This may be outside the scope of this MS, but wouldn't it be possible to explore these further using e.g., their existing XRF data, or models?

GENERAL Reply:

Thank you very much for reviewing our manuscript and providing such positive and constructive feedback. We fully agree with your assessment that the core narrative should be more prominent, the arguments require stronger quantitative support, and the data presentation needs to be clearer. We have undertaken major revisions to the manuscript based on all your suggestions and believe the revised version is significantly improved.

The previous version became too mired in technical details, diluting the core message about WAIS dynamics. To address this, we have streamlined and reorganized the Results section. Several highly detailed methodological descriptions and datasets have been moved to the Supplementary Material. We have completely redesigned the figures to enhance their clarity and interpretability.

Since the data in this paper is insufficient to support a glacial-interglacial scale interpretation, and the focus is not on the glacial-interglacial scale, we have removed the relevant content.

We have reclassified the core into three sections, reducing from the previous six.

We have rewritten the Discussion section to elaborate further on ice sheet dynamic mechanisms and provide quantitative comparisons. We have added a new section titled "Mechanisms of WAIS Variation and Climate Coupling" to the Discussion.

Specific details of the modifications have been addressed in the point-by-point responses.

COMMENT 1:

L41: The beginning of the first sentence “Our incomplete understanding” – reads like a double negative here. I leave it to authors but would suggest something like e.g., “our current understanding……is incomplete and limits our ability…”?

REPLY: We have rephrased this sentence (Line 40-41).

COMMENT 2:

L87-92: This could have been phrased a bit more clearly, e.g., they also do compare and show data from U1533 as well.

REPLY: This paragraph has been moved into the Method section in “Drill site description” (Line 369). And we have added sentences to indicate that the lithology and chronology of Sites U1532 and U1533 are comparable. Therefore, we selected Site U1532 for further analysis due to its higher

sedimentation rate (Line 374-381).

COMMENT 3:

L94: "other proxies" is a bit vague, can you expand/list here what these are?

REPLY: We have expanded "other proxies" (Line 74-77).

COMMENT 4:

L98/Figure 1: There are many more sites shown in the large-scale inset map showing Antarctica (yellow circles). These should be explained/cited/referred to in the figure caption.

REPLY: Other ODP/IODP sites in the large-scale inset map was used in discussion. However, in accordance with the feedback received, we have refocused the discussion on the mechanisms of ice-sheet change and removed the section on circum-Antarctic comparisons. As a result, these sites have also been omitted from the context map.

COMMENT 5:

L103: This section reads like it could have been in the methods section?

REPLY: We have removed this section to the first part of Method as "Drill site description" (Line 369).

COMMENT 6:

L105: lat-lon info for the core site should also be provided

REPLY: We have added the lat-lon information (Line 382).

COMMENT 7:

L106: please expand: what is the max age of 5.7 Ma is based on..?

REPLY: It is based on Gohl et al. (2021) paper, we have added the reference (Line 383).

COMMENT 8:

L124/Table 1: this table is very helpful, but breaks the flow a bit. Wonder if it should be in the supplementary or if it would even be more helpful to include few sentences in the main text along when

these parameters are presented in results?

REPLY: This table has been removed to Supplementary Material as Supplementary Table 1 and we have included some simple sentences in the results along with descriptions of these parameters (Line 134-170).

COMMENT 9:

L134/Figure 2: There is a lot of information presented in this figure, yet I feel the data is a bit lost because of the way it was plotted (too dense/small/narrow data plots). I think this figure would highly benefit from a better visualization. Data are plotted very small/narrow, which makes it difficult to see & compare all the variabilities & trends, there are also too many lines and small text within. Resolution is too low when zoomed-in. Figure text/caption is missing a lot of crucial details. More specific comments below:

- I only see age control points here, it was mentioned that age model was well established (L118), but as far as I can tell all data are plotted vs depth here ? Is the age model exactly the same as the published Gohl et al 2021? In that case I'd suggest plotting the data vs age. This would open up some space & help to make the actual data larger/visible. If crucial, these can be (and is already) shown in supplementary.

- The subtext below/above each parameter is helpful, but I'd suggest removing "less" and "more", as this does not provide much info. If necessary, (+) / (-) signs could be added instead of including more text within the figure.

- What do the red lower case "a" and "b" in (next to sed rate plot) represent?

- The orange rectangle marking the significant warming could have also been marked differently (There are a number of red dashed lines, so this is not very visible). And is there a reason why this rectangle is not extended into the ln(Ba/Rb) plot?

- In general: I'd also recommend choosing colorblind safe color palettes (combination of red and green lines could be an issue).

- Would also be good to clarify/add here that some of these results are compared to the shipboard data/measurements?

REPLY: We have reduced the amount of information and text in this figure, changed the point-line plots to line plots and widened them. We included a high-resolution version in the revised manuscript. We changed the order of the magnetic parameters on the graph, using different colors to represent mineralogical indicators, concentration indicators, and particle size indicators, respectively, to make it

easier to read.

The previous age model was exactly the same as the published Gohl et al. (2021), now we have added the age points identified by Rahaman et al. (2025). We have also included the age control points used in this article in the Supplementary Material as Supplementary Table 3. We have plotted the data vs. age in the revised Figure 2.

We have replaced "less" and "more" in the subtext below/above each parameter with (+)/(-) signs.

The red lower case "a" and "b" were supposed to represent core sections plotted in first-version supplementary material Figure S1 (glacial-interglacial scale variations). But we have removed the glacial-interglacial statement due to the small relevance with main text.

We have replaced the orange rectangle marking the significant warming with beige shadow. We have changed the red dashed lines to black dashed lines. And the ln(Ba/Rb) plot has been deleted since its small relevance with main text.

We have avoided using red and green together in the figure.

We have clarified in the caption that we used some shipboard data for comparison.

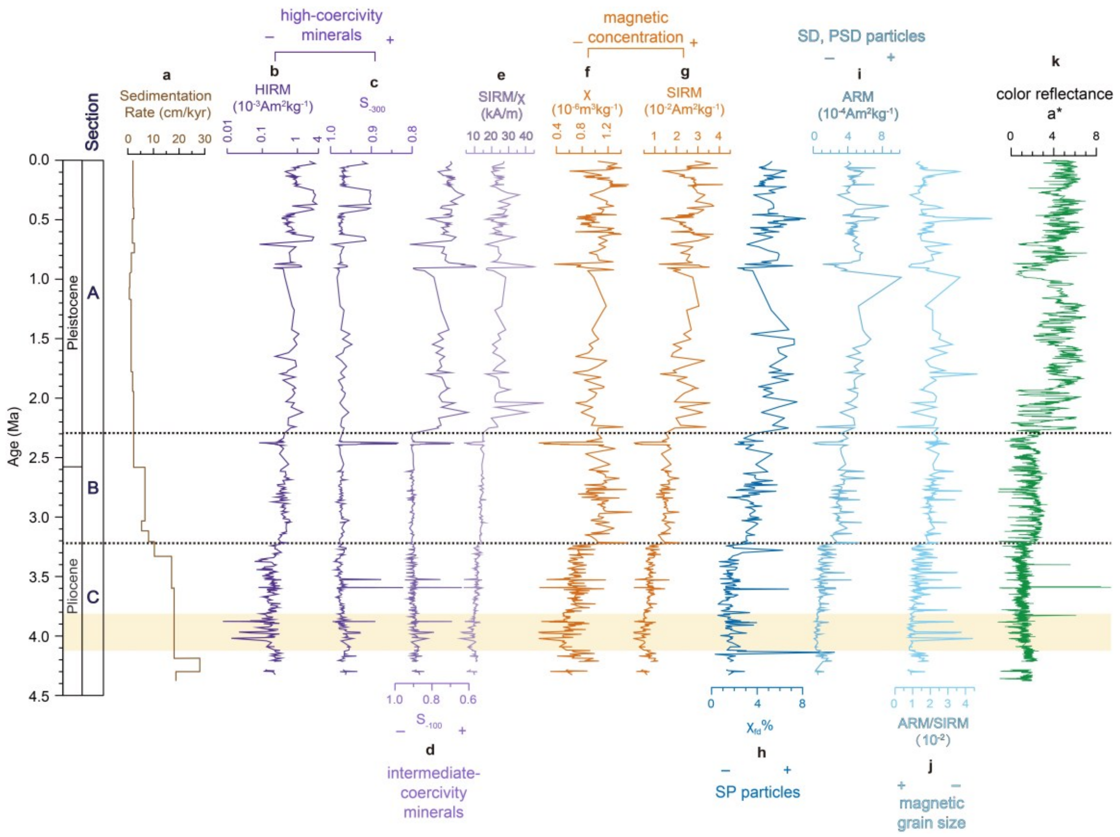

Fig. 2 | Variation of environmental magnetic parameters since ~4.33 Ma (the upper 300 m of the core) at Site U1532. *(a) Sedimentation rates. Magnetic mineralogy indicators (b) HIRM, (c) S_{-300}, (d) S_{-100} and (e) SIRM/χ are*

plotted in purple lines; magnetic concentration indicators (f) χ and (g) SIRM are plotted in orange lines; magnetic grain size indicators (h) χ_{fd}%, (i) ARM, and (j) ARM/SIRM are plotted in blue lines. (k) Shipboard color reflectance a[16] are plotted for comparison. Sections A-C are separated by dashed lines: A (39.01-0 m, 2.29-0 Ma), B (88.02-39.01 m, 3.21-2.29 Ma) and C (291.43-88.02 m, 4.33-3.21 Ma). Higher HIRM and lower S_{-300} indicate more high-coercivity minerals; lower S_{-100} indicate more intermediate-coercivity minerals; variable SIRM/χ indicates changes in mineral composition; higher χ and SIRM for higher magnetic concentrations; higher χ_{fd}% and ARM for more SP and SD/PSD particles; higher ARM/SIRM for smaller magnetic grain size. The light-yellow shaded area (~4.12-3.82 Ma) marks a period with extremely low concentrations of magnetic minerals, indicating a period of extreme warmth. SP: superparamagnetic; SD: single domain; PSD: pseudo single domain. See Supplementary Table 3 for age-depth model.*

COMMENT 10:

L176-177: both "downcore" and "down-core" are used

REPLY: We have used "downcore" uniformly.

COMMENT 11:

L180-182: Perhaps need to reword here a bit, otherwise it reads as it should be in discussion?

REPLY: We have reworded that sentence (Line 166-168).

COMMENT 12:

L250: Please write fully instead of "etc"

REPLY: We have changed that sentence and moved this content to Supplementary Material in Supplementary Text S2: "*Hysteresis loops and FORCs are effective in discriminating magnetic mineral coercivity, grain size distribution and various magnetic interactions and their strength*[25,26]."

COMMENT 13:

L268-272/Figure 6: Would be good to refer to sub-figures a-j in figure text, with a short info.

REPLY: We have renumbered the TEM images and modified the captions. To focus the main text more extensively on WAIS dynamics, we have placed TEM-related content in the Supplementary Material (Supplementary Text 5 and Supplementary Fig. 9).

COMMENT 14:

L292-297: Not sure any results are presented here? I think this part might be better suited for the methods section?

REPLY: We have deleted this paragraph since we don't use Ba/Rb in the revised manuscript.

COMMENT 15:

L340: I wonder if the shipboard sediment green-blue ratio is available, and if it would provide useful here to check in comparison to diatom/productivity (e.g., see Li et al., 2022, G-cubed: https://doi.org/10.1029/2022GC010350)

REPLY: A comparison between the green-to-blue ratio and diatom abundance reveals no coherent long-term trend. In contrast, diatom abundance, Si/Al, carbonate content, and Ba/Rb all exhibit consistent long-term variations (following figure). These results suggest that the green-to-blue ratio may not be a suitable proxy for long-term productivity in U1532 cores. Color change caused by oxidation in the top 40 meters of the core may compromise the ability of the green-to-blue ratio to represent productivity.

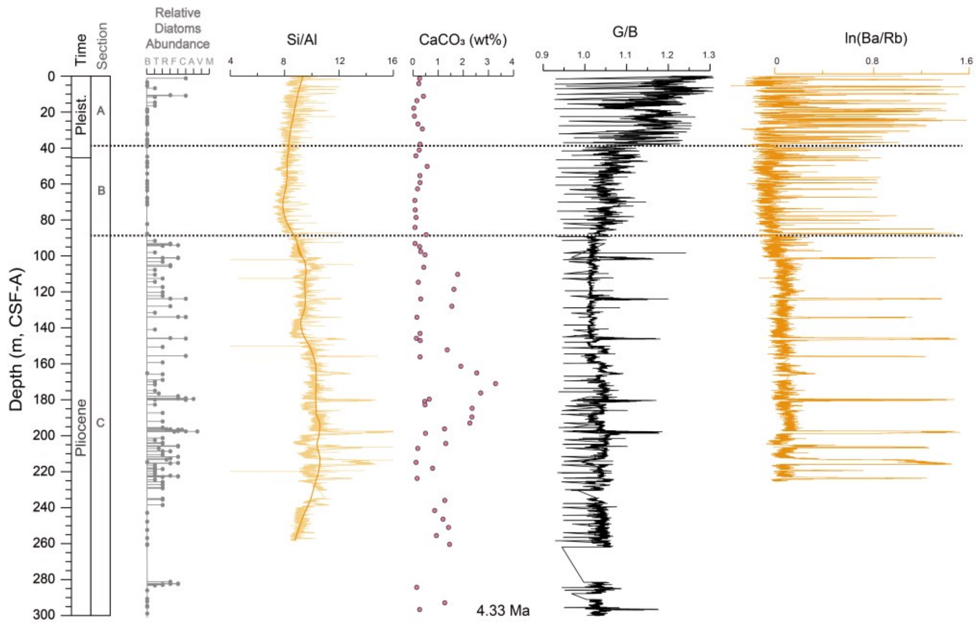

COMMENT 16:

L378: I thought the main focus was 0-300 m, but suddenly all cores from Site 1532 (0-800 m, Fig S9) as well as data from Site 1533 (Fig S10) are introduced here. This could have been mentioned/clarified in the intro.

REPLY: We have focused on the 0-300 m of the core and deleted contents related to previous Fig S9 and Fig S10.

COMMENT 17:

L381: I don't see IRD counts in Fig S9? Are pebble counts considered to be IRD? If so, please provide more details on this method. As far as I can tell this is only presence of pebbles, not counts?

REPLY: We made a mistake that the presence of pebble was considered to be IRD. We have deleted that paragraph and Fig S9 and focused on 0-300 m of the core.

COMMENT 18:

L385: Aren't the sites U1532 and U1533 too close to each other to say that these signals are not site-specific?

REPLY: We have deleted that sentence and content related to U1533 has been deleted.

COMMENT 19:

L443/Figure 9: There is again too much information in the figure, and a lot of details are missing in the Figure text. Having the age in y-axis is also not very helpful, regardless, all data should be plotted in same direction (i.e., warm-cold directions seem to be reversed with the Site 1096 Opal data). More specific comments below:

- What are the red dash lines on the ARM record?

- Some of the data they compare to have references on the figure (e.g., IRD, d18O records), while some don't (e.g., global SST, benthic stacks). This needs to be consistent, and follow the same in-text reference style

- The figure resolution is too low, some texts are almost not readable

- I assume the inset map in Figure 1 includes these other core sites & studies mentioned here, but they need to refer to the map here and provide a detailed info on these sites & comparisons.

- I also don't see any uncertainties mentioned or plotted here.

- I assume the blue dashed highlights/marks are the cooling phases they mention in text, but this should be explained here. There are also more intervals marked than what was mentioned as the 4 cooling phases ("four distinct transitions to a colder climate at 3.8 Ma, 3.2 Ma, 2.4 Ma, and 1.6 Ma") – so what do the 0.9 Ma and 4.1 Ma highlight?

- I think particularly the Global SST and the benthic stack records are plotted too narrow to fully

see the data variability in comparison with the ARM record. It would have been helpful to see ARM and the global stacks plotted together.. with regards to L434-437: I am not sure I see a “great consistency” or a “close alignment” based on how the data is presented here.

- “Figure 2 Sections” is often used in the MS, but I’d suggest a different/more descriptive label for these sections.

REPLY: We have revised the figure to improve clarity and have expanded the figure caption to include all necessary details, including explanations of symbols, lines, and data sources.

The red dashed lines have been removed. We have standardized the referencing style across all datasets shown in the figure. We have regenerated all figures at high resolution to ensure readability of text and details. We have revised the caption to explain the blue dashed highlights. Figure 2 Sections have been removed.

The core sites shown in the inset map of Figure 1 have been omitted, as previous circum-Antarctic comparisons lacked quantitative discussion. In response to the reviewers' comments, we have refined our discussion to focus on the mechanisms of West Antarctic Ice Sheet (WAIS) expansion. We have replotted the ARM and global SST change to improve the visibility of variability. The revised figure presents a comparison of global SST change, CO_2, and ARM records, focusing on the relationship between WAIS dynamics and global climate.

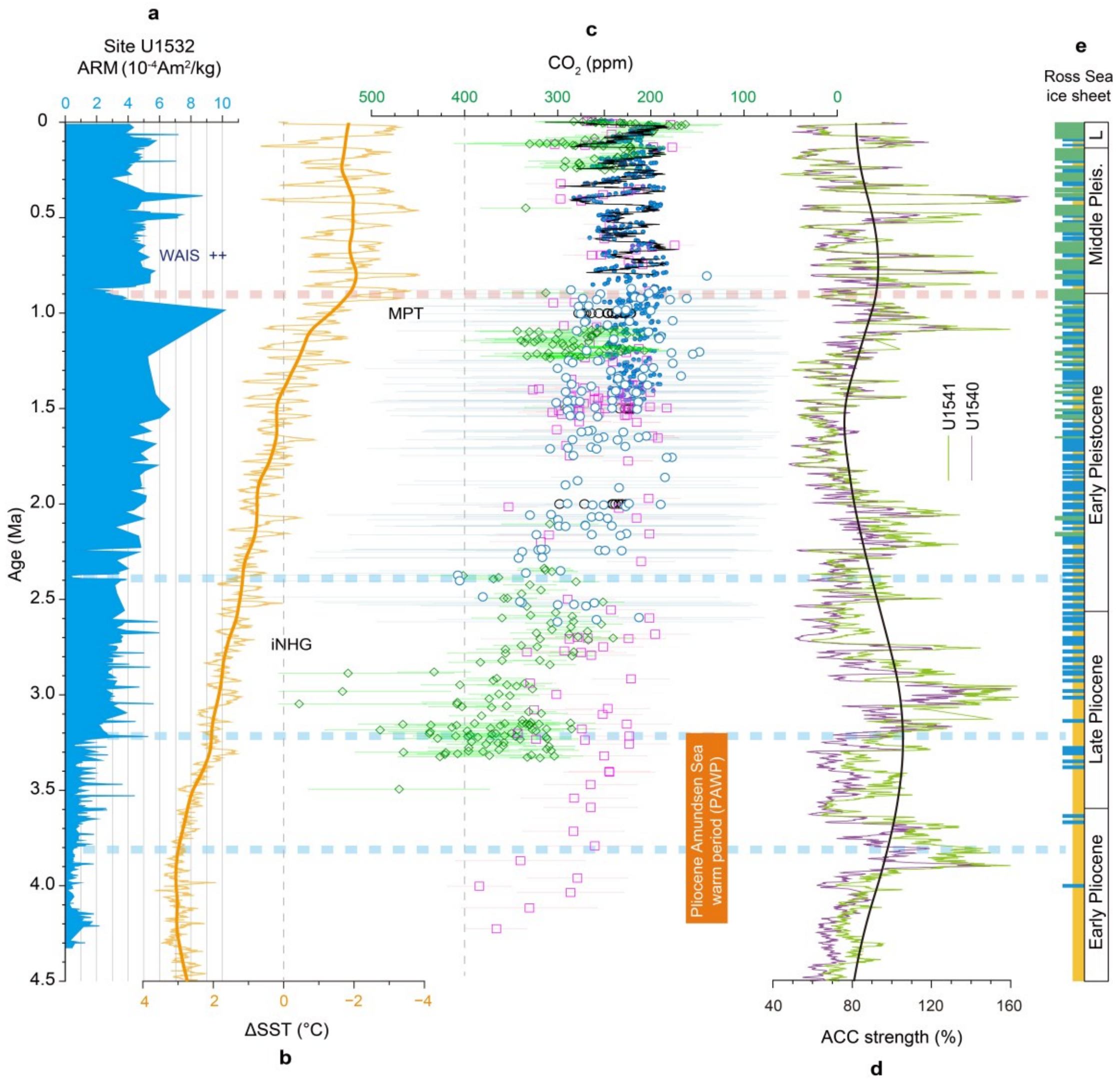

Fig. 6 | WAIS evolution since ~4.33 Ma recorded by ARM at Site U1532. *(a) ARM values of Site U1532, used as indicator for ice sheet advance/retreat. (b) Global sea surface temperature change (ΔSST)[48] and its LOWESS smoothed long-term trend; grey dashed line: preindustrial level[48]. (c) Proxy and ice-core CO_2 data. Magenta and green symbols are harmonized $\delta^{11}B$ values on planktonic foraminifera T. sacculifer and G. ruber, respectively[49]. Blue hollow circles are based on measurements on paleosols from the Chinese Loess Plateau[50]. Blue dots are from $\delta^{13}C$ of leaf wax[51]. Black lines are ice-core CO_2 record over the past 800 ka[52] and black hollow circles are early Pleistocene blue-ice CO_2 snapshots[53]. (d) Relative Antarctic Circumpolar Current (ACC) strength variations at Site U1540 and Site U1541[54]. Black line shows the 1-million-year smoothed ACC strength record[54]. (e) Modeled sediment facies in the Ross Sea, close to AND-1B (yellow = open ocean; blue = floating ice; green = grounded ice)[2]. Yellow and blue/green here correspond to the AND 1B diatomite (yellow) and diamictite (green) intervals[55]. Pliocene Amundsen Sea Warm Period (PAWP): based on the seismic correlation of sediment layers from the drill sites to the continental shelf, grounding zone wedges have been identified in the shelf sediments that predominantly formed during ice sheet retreat within an extended Pliocene warm period from 4.2-3.2 Ma[33]. MPT: Mid-Pleistocene Transition. iNHG: the intensification of the Northern Hemisphere glaciation. Blue dashed lines mark three transition points to a larger WAIS at ~3.8, 3.2 and 2.4 Ma. Pink dashed line marks a transition point to a relatively stable ice sheet at ~0.9 Ma.*

COMMENT 20:

L450-451: “magnetic minerals” used twice within the sentence – perhaps reword?

REPLY: We have rephrased that sentence.

COMMENT 21:

L460-462: both “the Middle Pleistocene Transition” and “MPT” are used

REPLY: We uniformly use the term "Mid-Pleistocene Transition (MPT)" throughout the manuscript.

COMMENT 22:

L499-505: Was the XRF scanning done throughout the 0-300 m interval? Please state.

ln(Ba/Rb) is shown in Figure 2. However, only the 145-149m and 197-201m intervals are shown for Br/Rb and Zr/Rb. There are some general statements in the supplementary info regarding how these element ratios change with glacial/interglacial transitions, yet it is difficult to assess if these are common features (or not), without seeing the actual full data.

REPLY: The intervals 145-149 m and 197-201 m were selected owing to their relatively high magnetic data density. Given that the current resolution is insufficient to resolve glacial-interglacial scale patterns, statements and XRF data descriptions pertaining to such cyclicity have been deleted as they bear limited relevance to the main conclusions. There is no XRF scanning data for the 258-300 m interval (Line 437-438).

COMMENT 23:

L525: Hope that the data will be made available upon publication, either along with the article or through a data repository. Unless there are other valid restrictions/reasons for not making data available, this does not follow the FAIR principles.

REPLY: The data has been uploaded with the revised manuscript.

COMMENT 24:

-I don’t think Figure S1 has been cited within the main MS

REPLY: This figure and relevant text have been deleted.

COMMENT 25:

-There is no Fig. 4 (Fig. 5 comes after Fig. 3), yet Fig 4 is cited in Supplementary text (i.e., under glacial-interglacial time scale magnetic analysis section)

REPLY: We have corrected the figure numbers.

COMMENT 26:

Figure S1: What are the numbers next to red rectangles refer to (1,2,3,4?).

IRD (e.g., "gradual onset of IRD during glacial-interglacial transitions" is mentioned few times here, but it is not mentioned what this is based on. Is there a IRD % data available? Please clarify.

REPLY: We have deleted this part. Since the data in this paper is insufficient to support a glacial-interglacial scale interpretation, and the focus is not on the glacial-interglacial scale, we have removed the relevant content. Future work will conduct detailed glacial-interglacial scale studies.

COMMENT 27:

Figure S2: Can you add a bit more details to the figure text here to clarify. E.g., there are 2 shaded boxes. And which HIRM values / intervals do not correlate with L values? Do you refer to all points within sections A, B, C, F ?

REPLY: We have changed this figure. In the previous version of the manuscript, several points with extremely high HIRM values in uppermost core were excluded from the L-ratio assessment. Based on the current core sectioning, since section A has undergone a change in mineral composition, we conducted L-ratio assessment on section A and sections B and C separately and found that the L-ratio is stable. Therefore, in this study, HIRM is influenced by the concentration of high-coercivity minerals. The L-ratio validation has been moved to the Supplementary Material (Supplementary Text 1 and Supplementary Fig. 1).

COMMENT 28:

Figure S4, S5. A short info describing the figures & a more detailed figure text are also needed here. Referring to main text and main figures in the MS makes it difficult for the reader to go back and forth.

REPLY: We have added more detailed figure text to these figures.

COMMENT 29:

Figure S8: I though the focus here was the 0-300 m of the core, why is data between 405.72-321.68 m presented here ..?

REPLY: This paragraph has been deleted and we have focused on the 0-300 m of the core.

COMMENT 30:

Figure S9 (and S10): I find using both highlights and arrows at the same time too much. I think the highlights are sufficient to show an increase/decrease and suggest removing the arrows not to make the figure too crowded. I also assume most of these are shipboard data, please also add references after each parameter.

REPLY: These figures have been deleted and we have focused on the 0-300 m of the core.

==

Reviewer #3

GENERAL COMMENT:

The manuscript by Zhang et al "Magnetic mineral properties of continental rise sediments in the Amundsen Sea reveal West Antarctic Ice Sheet dynamics since the early Pliocene", presents an interesting and relevant study focused on reconstructing the West Antarctic Ice Sheet (WAIS) dynamics since the middle Pliocene (~4.3 Ma) based on a sedimentary archive from the Amundsen Sea continental rise (Site U1532).

The authors use rock magnetism, transmission electron microscopy and XRF core scanner Ba/Rb ratio, alongside comparisons with kaolinite content and relative diatom abundance to determine the source of magnetic minerals and reconstruct ice sheet dynamics in the region (glacial-interglacial variability). One of the key findings is the possible collapse of the WAIS between 4.1-3.8. Additionally, the authors identify four intervals of colder climatic conditions between 4.1 Ma and the Mid-Pleistocene Transition, occurring at ~3.8, 3.2, 2.4, and 1.6 Ma.

I appreciate the effort to integrate various proxies to address an important paleoclimatic question. However, I must acknowledge that I am not familiar with the specific methodologies used in this study, so I am not in a position to critically evaluate that component of the work. That said, I would like to suggest a few points for consideration, which I believe could help strengthen the manuscript and enhance

its overall clarity.

GENERAL Reply:

We sincerely thank you for your time and thoughtful review, as well as for your positive acknowledgment of our study's importance. Below please find our point-by-point responses to each comment.

COMMENT 1:

In particularly I would like to highlight a recently published study by Passchier et al. (2025) "West Antarctic ice retreat and paleoceanography in the Amundsen Sea in the warm early Pliocene", which analyses the same sedimentary archive, focusing on the early Pliocene interval (5.2–3.6 Ma). Their findings appear to contrast with some of the conclusions presented here. Specifically, provenance data from 4.1 to 3.6 Ma in that study suggest relative WAIS stability during both glacial and interglacial periods, along with indications of a generally more expanded ice sheet. Given that their study presents a refined age model, detailed sedimentary facies interpretations, and a multi-proxy provenance dataset, I believe that a more explicit comparison with their results would considerably strengthen the discussion. It may also lead the authors to reconsider or further clarify some of their interpretations and objectives of their study.

REPLY: Observations by Passchier et al. suggest that the WAIS was generally more extensive during 4.1-3.6 Ma compared to the warm period of 4.6-4.4 Ma. However, due to the lack of data from 4.6-4.4 Ma in this study, a direct comparison cannot be established. Although the interval from 4.33 to 4.21 Ma represents a time period of ice sheet retreat in our study, a long-term episodic ice-advance trend can still be inferred from the gradual increase in ARM until the Mid-Pleistocene Transition. This is consistent with the generally more extended ice sheet between 4.1 and 3.6 Ma described by Passchier et al. (Line 301-306). Therefore, I believe these two studies are not contradictory.

COMMENT 2:

I suggest that the authors consider moving some of the data presented in Figure 2 to a supplementary figure, as the figure currently appears too busy and maybe difficult to follow by non-specialist readers.

REPLY: We have modified Figure 2 and reduced some plots. Magnetic mineralogical, concentration-dependent, and grain-size-dependent parameters are now displayed in distinct colors for

better readability.

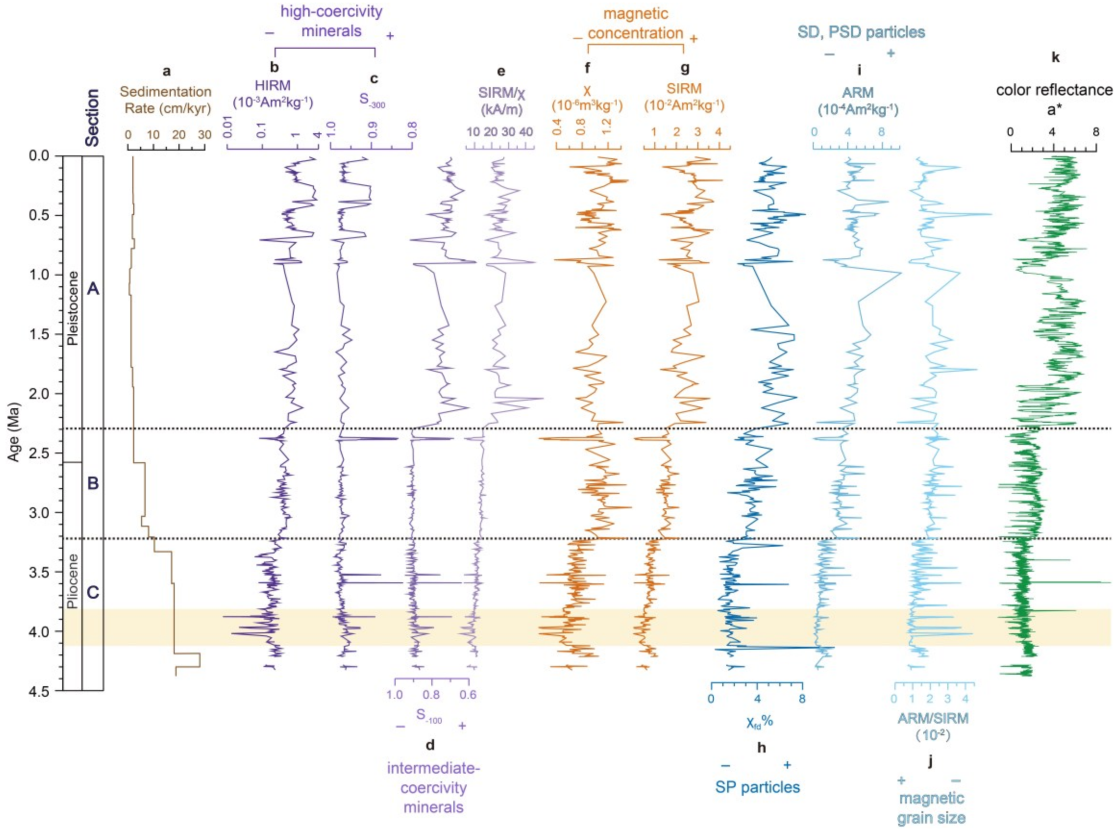

Fig. 2 | Variation of environmental magnetic parameters since ~4.33 Ma (the upper 300 m of the core) at Site U1532. *(a) Sedimentation rates. Magnetic mineralogy indicators (b) HIRM, (c) S_{-300}, (d) S_{-100} and (e) SIRM/χ are plotted in purple lines; magnetic concentration indicators (f) χ and (g) SIRM are plotted in orange lines; magnetic grain size indicators (h) χ_{fd}%, (i) ARM, and (j) ARM/SIRM are plotted in blue lines. (k) Shipboard color reflectance a*[16] are plotted for comparison. Sections A-C are separated by dashed lines: A (39.01-0 m, 2.29-0 Ma), B (88.02-39.01 m, 3.21-2.29 Ma) and C (291.43-88.02 m, 4.33-3.21 Ma). Higher HIRM and lower S_{-300} indicate more high-coercivity minerals; lower S_{-100} indicate more intermediate-coercivity minerals; variable SIRM/χ indicates changes in mineral composition; higher χ and SIRM for higher magnetic concentrations; higher χ_{fd}% and ARM for more SP and SD/PSD particles; higher ARM/SIRM for smaller magnetic grain size. The light-yellow shaded area (~4.12-3.82 Ma) marks a period with extremely low concentrations of magnetic minerals, indicating a period of extreme warmth. SP: superparamagnetic; SD: single domain; PSD: pseudo single domain. See Supplementary Table 3 for age-depth model.*

COMMENT 3:

In addition, I was wondering why the intervals of high paleoproductivity, inferred from Ba/Rb ratio do not correspond to higher diatom abundance?

REPLY: Peaks in Ba/Rb and diatom abundance co-occur and are both found in the greenish layers that represent interglacial periods (following figure). The Ba/Rb ratio also exhibits a long-term trend

consistent with that of diatom abundance, albeit more subtle. We have removed the Ba/Rb ratio from the manuscript, as they were originally intended for glacial-interglacial scale discussions. However, due to insufficient data density to reliably examine depositional patterns at this scale, and considering their limited relevance to the manuscript, these sections have been deleted.

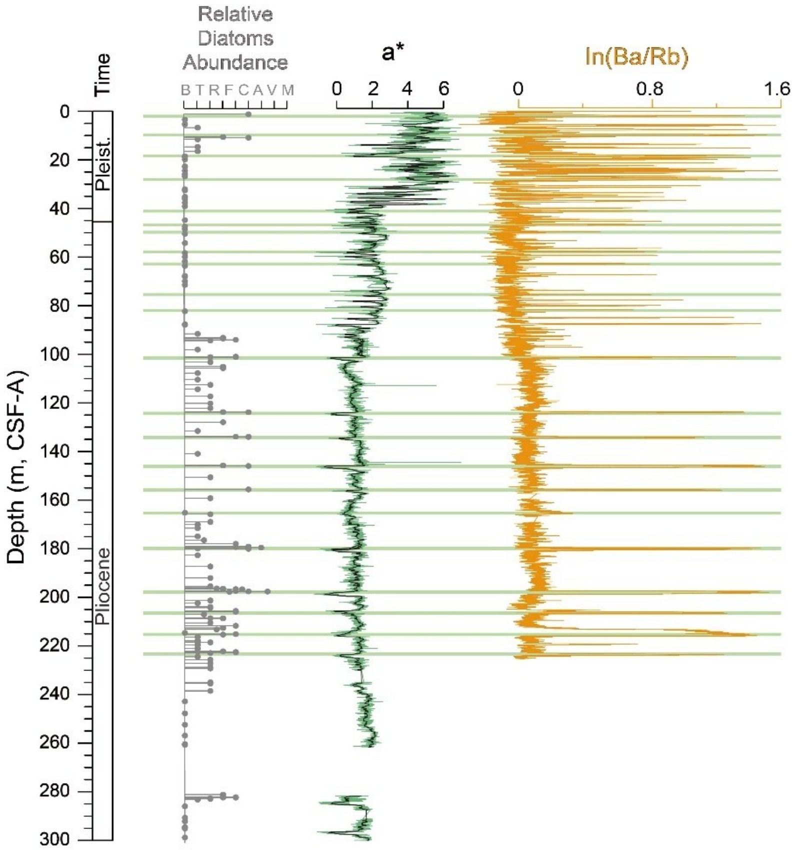

Furthermore, a comparison between the Si/Al ratio and diatom abundance identified a coherent long-term trend.

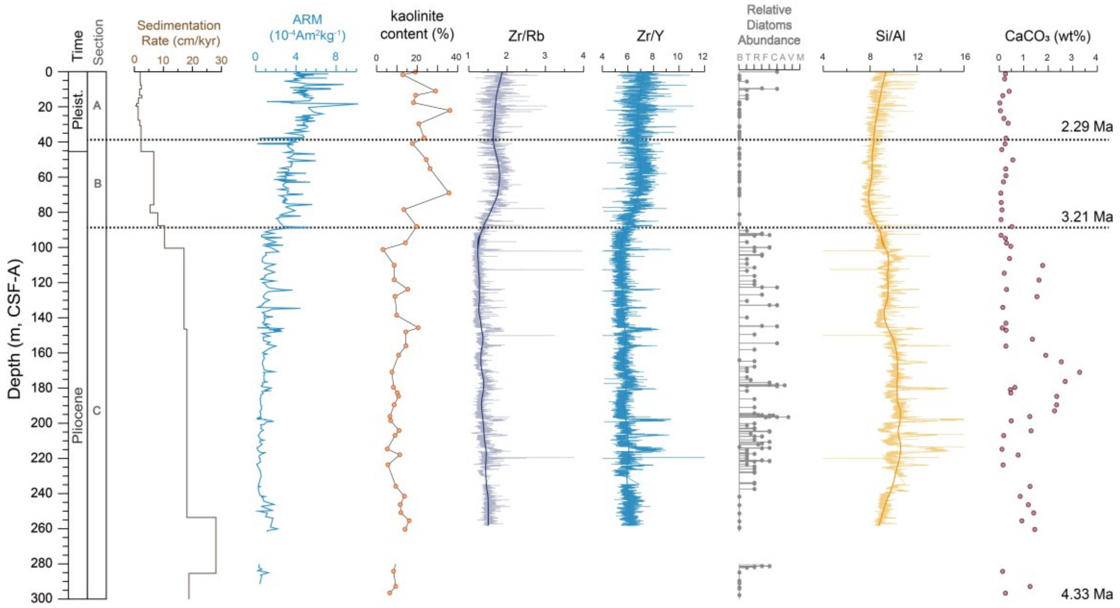

Supplementary Figure 12. Sedimentation rate, ARM, kaolinite content[16]*, Zr/Rb, Zr/Y, relative diatoms abundance*[16]*, Si/Al, and $CaCO_3$ content*[16] *of the 0-300 m core at Site U1532.*

Response to Reviewer's comments

Manuscript No.: NCOMMS-25-21134B

In response to the feedback on our manuscript, we have revised the paper accordingly. The following responses are provided in detail:

Reviewer #1

GENERAL COMMENT:

The authors have undertaken a rigorous review of the manuscript considering all earlier comments. Rebuttal is robust and supported by additional information and analyses that are sufficient to address my initial concerns. The rearrangement and refocusing of the text is most helpful in clarifying the narrative, particularly regarding the movement of highly technical and/or secondary discussions to the Supplementary Material. In line with my first review, I offer no challenges to the fundamental science within or the authors' conclusions, and find it to be a worthwhile addition to the literature. I believe the revised manuscript is well suited for publication pending minor grammatical corrections, with a non-exhaustive list of corrections presented below.

GENERAL REPLY:

We sincerely thank you for your positive assessment of our revised manuscript and for acknowledging the efforts made in addressing the previous comments. We sincerely appreciate your time and constructive feedback, which have greatly helped in improving the clarity and focus of our work. We have carefully reviewed your suggestions and have incorporated them into the manuscript. Below, please find our point-by-point responses to your specific comments.

COMMENT 1:

Line 76 and Supplementary Fig 12: "relative diatom abundance".

REPLY: Corrected.

COMMENT 2:

Line 91: Consider moving this statement on interpretations to the start of the following paragraph near line 134, as the rest of this section only deals with result description.

REPLY: We have moved this statement to the to the start of the following paragraph (Line 135).

COMMENT 3:

Line 93: Can you please indicate the depth ranges of each section?

REPLY: We have indicated the depth ranges behind each section (Line 95-101).

COMMENT 4:

Line 94: Perhaps move the sentence on Supplementary Table 2 to the end of this paragraph, around line 104. As written, it places undue weight on the data table and distracts from the key descriptions that follow.

REPLY: The sentence has been removed to the end of this paragraph (Line 103-105).

COMMENT 5:

Line 123 (Figure 3): Data points overlay the text annotations.

REPLY: We have placed the data points on the bottom layer of the figure.

COMMENT 6:

Lines 133 – 161: This section is incredibly dense and packed with information, but personally still very difficult to read. I would encourage the authors to take every effort to simplify this section, but concede that there is a limit to what can be done while retaining the essential information.

REPLY: We have done our best to condense this section by cutting eight full lines and have worded it to remain accessible to non-specialists (Line 134-157).

COMMENT 7:

Line 141: … indicating a significantly reduced magnetic mineral concentration in the sediments.

REPLY: Corrected.

COMMENT 8:

Line 142 – 149: The back and forth comparison of sections B and C is quite confusing for me, as it is difficult to recall both which has higher/lower values for each parameter and which we are currently talking about.

REPLY: Given that the meaning of each proxy was already explained in Section C and is labeled in Figure 2, we streamlined Section B to present only the magnetic results, avoiding redundancy and potential confusion (Line 143-147).

COMMENT 9:

Line 214: indicates

REPLY: Corrected.

COMMENT 10:

Line 245: ARM increase resulting from

REPLY: Corrected.

COMMENT 11:

Line 247: Site U1532

REPLY: Corrected.

COMMENT 12:

Line 256: ice sheet' ?

Line 256-257: Rephrase: (suggestion) On the other hand, over the million-year timescale, the increasing glacial over-deepening of the shelf could make it difficult for the grounding line of the ice sheet to advance to the shelf edge during subsequent glacial intervals.

REPLY: We have rephrased that sentence (Line 261-263).

COMMENT 13:

Line 296: Acronym for ΔSST is currently introduced in Line 303.

REPLY: Corrected.

COMMENT 14:

Supplementary Figure 11: interpretations misspelled (magnetic sconcentration)

REPLY: Corrected.

COMMENT 15:

Supplementary Figure 13: a) I don't see any black line for the 1 million year smoothed record. b) 413-kyr filtered ACC strength.

REPLY: This figure has been deleted.

==

Reviewer #2

GENERAL COMMENT:

I would like to thank the authors for their comprehensive revision and for thoroughly addressing all my comments & concerns. I think the MS has significantly improved. I only have few minor comments & suggestions, listed below per line numbers.

GENERAL Reply:

Thank you for your valuable feedback and for recognizing the improvements in our revised manuscript. We are pleased to hear that you find the manuscript significantly enhanced. We have carefully considered your additional minor comments and suggestions. Below, please find our point-by-point responses and the corresponding revisions made to the manuscript.

COMMENT 1:

L57: Should "ASE" be shown/mentioned in the Figure 1 map/caption. Also, regarding the Fig1. figure caption: Since both U1532 and U1533 are shown in the map, perhaps the short title "U1532 Site map" could be changed/removed.

REPLY: We have changed the figure caption of Fig. 1 as "A regional map of the Amundsen Sea Embayment (ASE) and coastal Marie Byrd Land (MBL)" (Line 85).

COMMENT 2:

L95-105: I would suggest describing the properties and differences in these sections in full sentences, rather than listing properties & values (e.g., In Section C, xxxx are observed. instead of "Section C: …")

REPLY: We have changed it (Line 95-101).

COMMENT 3:

L97: S-300 "values" approach 1. (also L134)

REPLY: We have changed it (Line 96 and 137).

COMMENT 4:

L282: planktonic foraminifera listed here should be in italic font.

REPLY: We have changed the font (Line 287-288).

COMMENT 5:

L301-306: I found this part to be a bit confusing. In L301-302: 4.1-3.8 Ma interval is referred to as min WAIS extent corresponding to peak warm SSTs, while in L304-305, the same interval is referred to as a "cooling trend". Please clarify.

REPLY: Thank you for pointing out that the statements "minimum WAIS extent at 4.1-3.8 Ma" and "cooling trend during 4.1-3.6 Ma" appear contradictory. We agree that both the warmest extreme and an overall cooling trajectory can coexist within the same interval; however, we found that the sentence referring to the cooling trend is unnecessary at this location and may confuse readers. We have therefore removed it.

COMMENT 6:

L308: Looking at Fig 6 (and also Supl Fig 13), I don't see a significant change at 3.8Ma based on the ARM.. To me it seems to be relatively stable until 3.2 Ma.. Are these "key transitions" based on U1532, or other data? I think it would help to justify the timings of key transitions a bit more here?

REPLY: 3.8 Ma is a transition point based on our ARM data at Site U1532 (following figure). But it is not very obvious so we have deleted it.

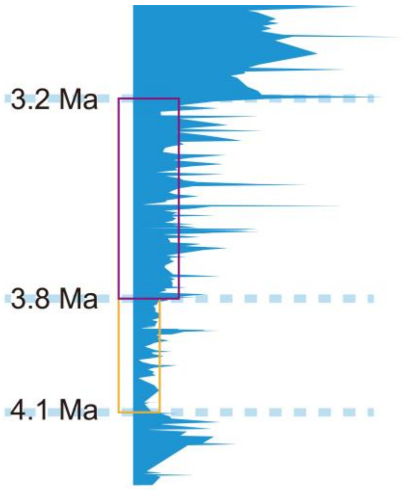

COMMENT 7:

L308: Could refer to an additional figure or change "Figs. 6" to "Fig. 6" (..also note that, in general there are too many references to Fig 6 on this page)

REPLY: We have corrected it to "Fig. 6" and we have decreased the amount of "(Fig. 6)" on this page.

COMMENT 8:

L357: Sentence starting with "Although" reads like missing something

REPLY: We have deleted "Although" (Line 349).

COMMENT 9:

Suppl. Fig. 10: What do the blue bands represent? Please provide more details in the figure text. It would also be good to include a short sentence to highlight & remind that the full record (0-800m) is presented in this figure, however the focus of this study is the upper ~0-300 m?

REPLY: We have changed the caption of Supplementary Figure 10.

Supplementary Figure 10. Downcore variation of magnetic susceptibility (MS), kaolinite content, total organic carbon (TOC), interstitial water sulfate, Mn and Fe concentrations at Site U1532. Blue bands mark intervals of elevated MS and kaolinite content. The full record (0-800 m) of Site U1532 is presented here to clarify that downcore dissolution does not affect the characterization of magnetic concentration and grain size based on magnetic parameters. The focus of this study is the upper ~0-300

m core.

COMMENT 10:

Suppl. Fig. 11: Would suggest using "Age" instead of "Time". And perhaps also "Ma" instead of "ka" to be consistent throughout the paper.

REPLY: We have changed that in Suppl. Fig. 11.

COMMENT 11:

Suppl. Fig. 13:

"Black line shows the 1-million-year smoothed ACC strength record" – I unfortunately could not see a black line here.

"Both gray and orange boxes mark phases of ACC decline, with gray indicating WAIS expansion and orange representing WAIS retreat. "

In general, I think it would help to have highlights/bands instead of boxes. Regardless, I found the argument here a bit confusing (also see comment above regarding key transitions), most of these intervals seem relatively stable (e.g., 3.8-3.6, 3.4-3.2, 2.1-1.7; etc) -- are the changes within these intervals significant?

REPLY: This part has been deleted according to Reviewer 4's comment 6.

==

Reviewer #4

GENERAL COMMENT:

The authors' study is built upon the assumption that subglacial erosion by glaciers draining into the Amundsen Sea Embayment leads to export of magnetic minerals from the ice sheet, and that these are transported across the continental shelf, before being deposited in the sampled drifts. Sediment cores sampling the drifts then enable a reconstruction of ice sheet changes. Previous studies discussed in this paper support that WAIS (even ASE continental shelf) is indeed the source of terrigenous material in the these drifts – although this is perhaps less certain than the authors suggest. In my opinion the main result of this paper (ice sheet growth from 5Ma until stabilization at about 0.9Ma) very much warrants

publication and is supported by clear discussion and figures. (Although “stabilization” should more clearly acknowledge that transient collapses of WAIS might have occurred since 900 ka). Some of the more detailed discussion/interpretation is also interesting, but as I have referred to below, it is not justified by the available data and this paper would be a lot stronger without it. Particularly the references to events and thresholds. See specific comments as follows.

Finally, I think the title should reflect some uncertainties here, e.g. "West Antarctic Ice Sheet advance since the early Pliocene", since the episodic nature of the advance is questionable, and it's not so clear where the sediments were sourced from.

GENERAL Reply:

We sincerely thank you for your time and thoughtful review, as well as for your positive acknowledgment of our study’s importance. We have made changes to our discussion concerning the “transition points”. We have changed the title to "West Antarctic Ice Sheet advance since the early Pliocene". Below please find our point-by-point responses to each comment.

COMMENT 1:

One question to me is the nature of the cross-shelf transport and subsequent deposition, and dilution of glacigenic material (indicative of ice dynamics) with other material. Although this is discussed (L187) I think it would be good if a version of Fig 2 was provided where quantity X is plotted in units of flux (X/m2/yr), rather than concentration (X/kg). That would give you the actual supply rate of glacigenic debris. It should be easily calculated from sedimentation rate and density?

REPLY: We sincerely thank you for this important suggestion. We have conducted an analysis accordingly. We multiplied several magnetic mineral concentration indicators (χ, HIRM, SIRM, and ARM) by the mass accumulation rate (MAR, calculated as dry density × sedimentation rate). The results are presented in the figure below.

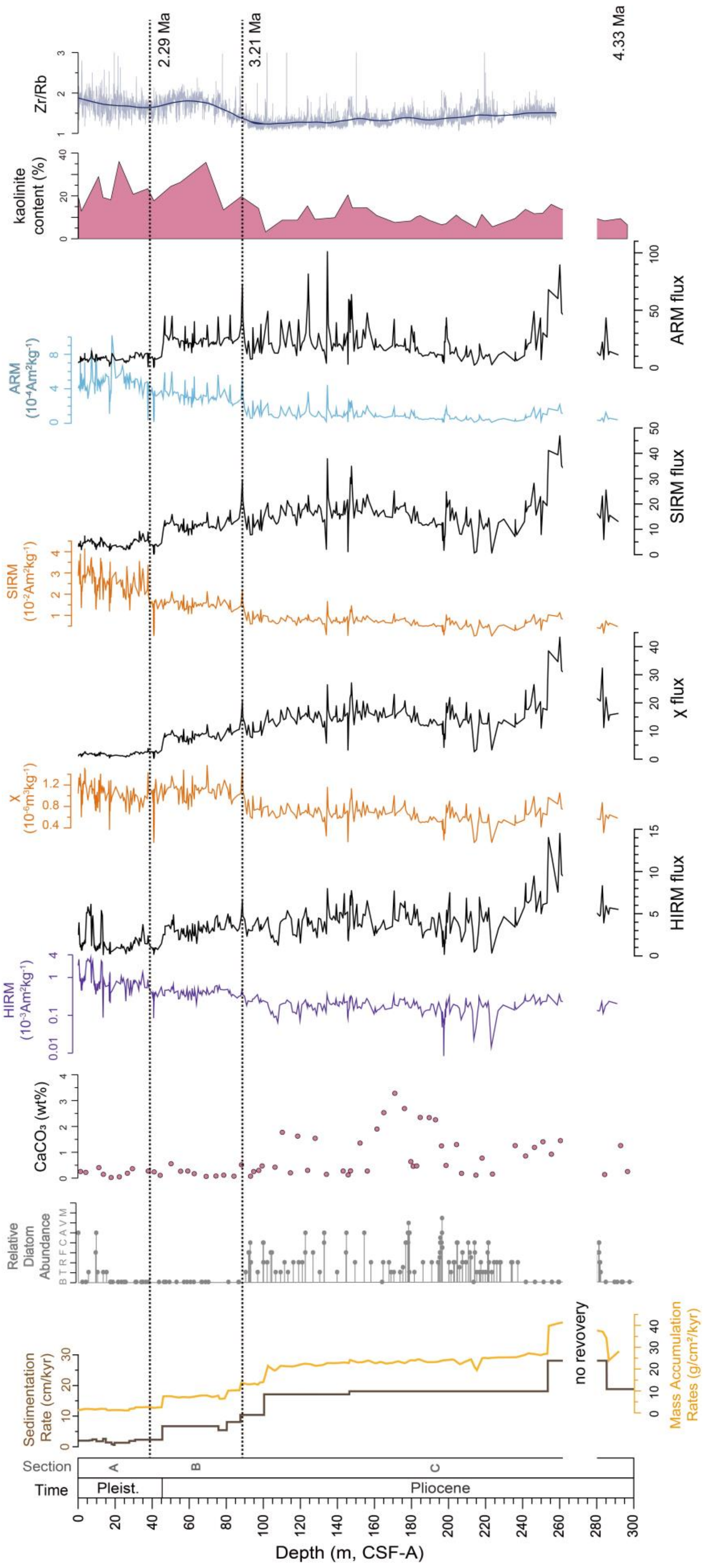

Zr/Rb
2.29 Ma
3.21 Ma
4.33 Ma
kaolinite content (%)
ARM (10⁻⁴Am²kg⁻¹)
ARM flux
SIRM (10⁻²Am²kg⁻¹)
SIRM flux
χ (10⁻⁶m³kg⁻¹)
χ flux
HIRM (10⁻³Am²kg⁻¹)
HIRM flux
CaCO₃ (wt%)
Relative Diatom Abundance
B T R F C A V M
Sedimentation Rate (cm/kyr)
Mass Accumulation Rates (g/cm²/kyr)
no revovery
Section
A
B
C
Time
Pleist.
Pliocene
Depth (m, CSF-A)

Our analysis reveals that the MAR of the studied core closely follows the sedimentation rate and is strongly modulated by variations in biogenic components (e.g., relative diatom abundance). Consequently, the term ARM × MAR conflates the "terrigenous magnetite flux" with a noise term, "magnetite concentration × biogenic MAR", which obscures its physical meaning and could lead to misleading interpretations. As noted in our manuscript, χ, HIRM, and ARM all show positive correlations with kaolinite content (Figure 4). Kaolinite has been established by prior studies as an indicator of input from a specific ancient provenance beneath the ice sheet, linked to ice-sheet advance phases (Line 204-208, 211-213). Therefore, the co-variation of ARM with kaolinite itself serves as a direct tracer of ice-sheet erosion of this source region, circumventing the uncertainties introduced by secondary calculations using the total MAR.

Furthermore, during the ice-sheet growth period from 4.1 to 0.9 Ma, ARM values exhibit positive correlations with reconstructed sea level, deep-sea temperature, sea surface temperature, and benthic $\delta^{18}O$ (Supplementary Figure 13). This consistency with multiple independent climate proxies indirectly corroborates ARM as a reliable indicator of ice-sheet dynamics (Line 255-257).

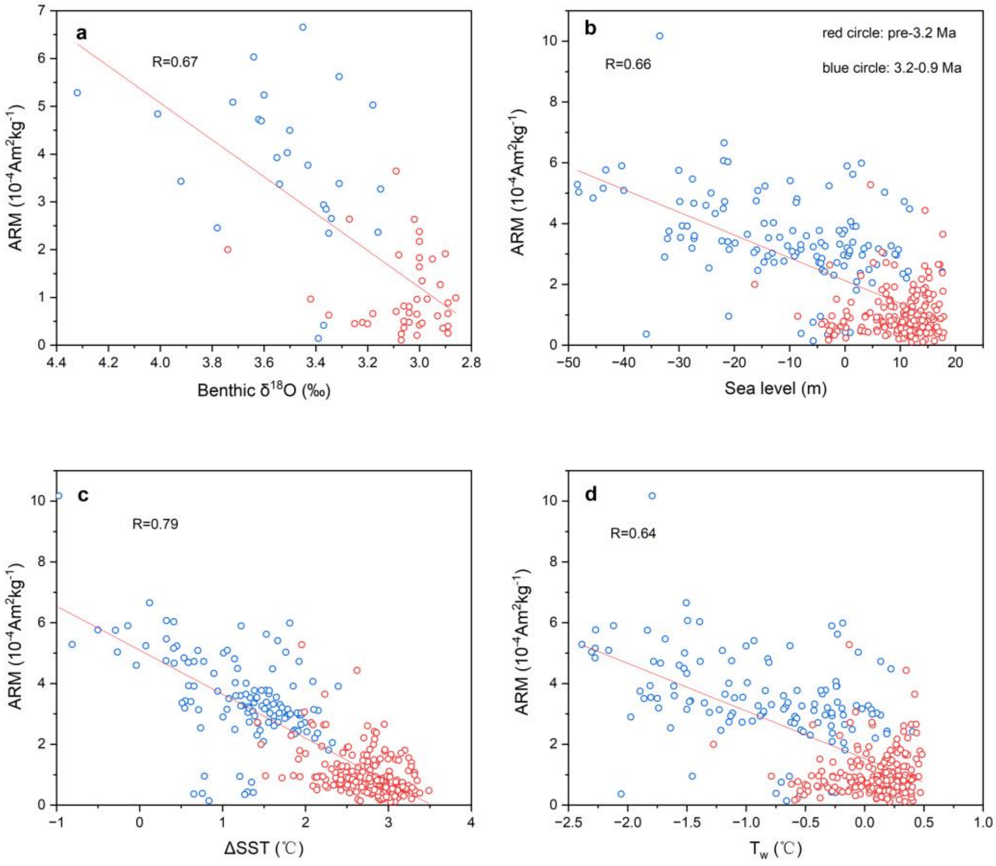

Supplementary Figure 13. Correlation of ARM with global climate proxies. (a) ARM vs. benthic δ^{18}O; (b) ARM vs. sea level; (c) ARM vs. global sea surface temperature change (ΔSST); (d) ARM vs. deep ocean temperature (T_w). R: Pearson's correlation coefficient.

COMMENT 2:

Another question is the source of the sediments. The authors argue that the clay minerology supports a source region on the Amundsen Sea Embayment (ASE) continental shelf (L208), and that "Terrigenous sedimentation on the Antarctic continental rise is controlled by glacial processes that generate glaciogenic detritus on land and the continental shelf, which is then transported downslope via gravity flows—such as slumps and turbidity currents—through deep-sea channels to the continental slope and rise". Although there are indeed troughs cut into the shelf north of Pine Island/Thwaites, the floors of these troughs still slope upwards towards the shelf break (BedMachine Antarctica). How are these sediments transported uphill except by sporadic ice advances under peak glacial conditions? Dependence on local cross-shelf ocean transport (e.g, by outwards flowing buoyant meltwater plumes) opens up many further questions regarding how even small changes in ocean circulation will bias transfer of sediments from the ASE ice stream groundling lines to the core site. I further question how this occurs specific to the core location given that there is a bathymetric rise at the edge of the shelf, between the core site and ASE (just to the right of the word 'Drift' in Fig 1). If the drift is mainly capturing suspended particles in eastward flowing bottom currents (L192), which I assume here is the ACC, what is the origin of these particles? Probably not the ASE, except during peak glacial conditions when the ASE grounding line is right at the shelf edge. So, yes I'm sure the record reflects ice dynamic changes somewhere in WAIS, but I would suspect over a wider region than ASE itself.

REPLY: Our original manuscript intended to convey that sediment delivery to the continental margin is primarily achieved through glacial advances, and we acknowledge that this point could have been articulated more clearly. To address this, we have added the following clarifying statement: "This process occurs primarily when the ice sheet advances onto the continental shelf during glacial periods" (Lines 187-188). This is consistent with our earlier statement that terrigenous downslope supply is significantly reduced during warmer interglacial periods (Lines 192-194).

Concerning the potential influence of ocean currents in transporting and mixing sediments, we wish to emphasize two key lines of evidence from our study that argue against a dominant current-driven

signal at our site: (1) Shipboard clay mineral analyses consistently point to an ASE shelf source; (2) The detrital isotope data (Sr-Nd-Pb) presented in Rahaman et al. (2025) were explicitly compared to potential current influences. That analysis concluded that ocean currents played a negligible role in sediment delivery to our core site, reaffirming the ASE as the dominant and consistent provenance. We have expanded the discussion in the manuscript (Lines 208-216) to integrate this finding, which strengthens our conclusion that the sedimentary record at Site U1532 archives ice-dynamic signals predominantly from the ASE.

Regarding the specific question about the bathymetric rise between the ASE source and the core site, we propose several viable mechanisms that could facilitate sediment bypass: (1) The local topographic high may not be sufficiently pronounced to act as an absolute barrier; (2) Sediments may be routed through alternative, adjacent channels; (3) The ice-sheet advances during glacial maxima could provide the necessary energy to overcome minor topographic obstacles. Most importantly, the clay mineral and geochemical provenance results, which confirm a clear signal from the ASE in the core, demonstrate that the bathymetric rise between the source and the site did not act as an effective barrier to sediment transport.

COMMENT 3:

L296 / Fig 6. SST is not so directly relevant for AIS dynamics, since it's the deep ocean temperature that drives ice loss (specifically CDW in ASE as shown by many studies). Especially in Fig 6 the Clark et al. SST compilation is nearly all from the tropics and Northern Hemisphere. Of course, over glacial/interglacial timescales then CO_2, SST and deep water temperature are well correlated. If deep ocean temperature is more insightful – maybe plot the Rohling et al (2022, Rev Geophys) reconstruction in Fig 6? Some modelling studies (Garbe et al. 2020 Nature, Golledge et al. 2021 GRL, Chandler et al. 2025 Comms Earth & Env) have suggested a deep ocean temperature threshold close to present-day is sufficient to trigger WAIS collapse, does that tie in with your reconstruction?

REPLY: We have updated Figure 6 by incorporating the deep ocean temperature reconstruction from Rohling et al. (2022) for direct comparison, as recommended (Line 313-318). Additionally, we have included a Southern Hemisphere mid-to-high-latitude SST record to provide a more regionally relevant surface temperature context. We note that the long-term trends of this Southern Hemisphere SST are consistent with the global compilation. Given the strong correlation between SST and ARM

(Supplementary Figure 13), we have retained the SST comparison in our analysis. We have also briefly addressed the concept of a deep-ocean temperature threshold for WAIS stability in the discussion (Line 316-318, 325-326).

COMMENT 4:

L307-320. Picking these three transitions at 3.8, 3.2, 2.4 is perhaps optimistic – I can't really see these standing out in the Fig 6 ARM record, and given my discussion above I'm very much not convinced this level of interpretation is justified.

REPLY: We agree with the reviewer's assessment. In response, we have removed the identification of the transitions at 3.8 Ma and 2.4 Ma from the manuscript, as they were not standing out. However, we consider the shift at ~3.2 Ma to be a more distinct feature in the ARM record (Fig. 6), and therefore have retained this single transition in our interpretation (Line 318-326).

COMMENT 5:

L326: 400ppm CO_2 threshold for marine ice sheets – this is an unhelpful generalization which doesn't need propagating through the literature. The Eurasian Ice Sheet was in parts a marine ice sheet (just like Antarctica is in parts) but its disintegration started long before we reached 400 ppm CO2. Suggest this related discussion should be removed or at least very clearly given a regional context if it's just Antarctica.

REPLY: In the revised manuscript, we have specified that the ~400 ppm CO_2 threshold discussed pertains specifically to the Antarctic Ice Sheet (Line 322), thereby providing the regional context as suggested.

COMMENT 6:

L333, Link to ACC strength, CDW upwelling and WAIS response. Probably the ACC strength isn't the full story here. Other factors come into play when considering heat transport to WAIS, including (i) meridional changes in core of the SH westerlies that controls location of Ekman driven CDW upwelling, (ii) CDW temperature, in turn related to NADW formation in N Atlantic; (iii) strength of slope current. So yes, interesting to plot ACC strength alongside the other records in Fig 6, but later statements ("new finding of a phase shift at 1.5Ma on 400ka eccentricity bands) is too speculative to me. The proposed relationship isn't clear to me at all, in Figs 6 or S13. At the very least can you plot the ARM and ACC

records against each other in a scatter plot and show the envelopes of the scatter are significantly different before/after 1.5Ma?

REPLY: We have removed the speculative discussion regarding a phase shift between ACC and WAIS on the 400-kyr eccentricity band. We have expanded the discussion on the factors controlling heat transport (Lines 331-346) to include the other critical mechanisms highlighted by the reviewer.

COMMENT 7:

L350-366 (Summary), also the Abstract. Some conclusions here (episodic expansion and link to temperature thresholds; synchronicity with major global events) should be presented with more acknowledgement of uncertainty. Or really removed – the headline message that the U1532 record shows a gradual trend of increasing WAIS glaciation through the last 5 Ma, until 900 ka, is already a great result, and I think the deeper but more speculative interpretation isn't warranted or necessary.

REPLY: We have modified both the Abstract (Lines 45-49) and the Summary section (Lines 347-358) accordingly. Specifically, we have removed references to synchronicity with major global events and have toned down language regarding episodic expansion to present a more measured interpretation. However, we have chosen to retain the concept of a threshold around 3.2 Ma, as our records indicate a clear onset of sustained WAIS growth at this time. We have revised the text to explicitly acknowledge the uncertainties associated with this interpretation.

COMMENT 8:

Fig 5: A "Stable large ice sheet" extending to the shelf break isn't a good depiction of the Pleistocene Antarctic Ice Sheet, which has repeatedly retreated from the shelf edge during interglacials. Perhaps "Pleistocene glacials" would be a more accurate title?

REPLY: We have changed "Stable large ice sheet" to "Pleistocene glacials" in Fig. 5.

COMMENT 9:

L59 "changing at the fastest rate" needs some clarification, i.e., in terms of what metric?

REPLY: We have clarified that the Amundsen Sea Embayment (ASE) is the largest contributor to Antarctic ice mass loss (Line 56-57).

COMMENT 10:

L61 “Holocene records link this retreat to inflow of CDW”. Likely true that CDW was the underlying case of 1940s retreat, but the cited Holocene records don’t actually resolve this recent period. A stronger case could be made by linking recent retreat to observed CDW warming?

REPLY: We have linked recent retreat to observed CDW warming (Line 59-62).

COMMENT 11:

L155 Sentence shouldn’t start with “And”.

REPLY: We have deleted the “and”.

COMMENT 12:

L182 Affection -> affect.

REPLY: We have changed this word.

Response to Reviewer's comments

Manuscript No.: NCOMMS-25-21134C

In response to the feedback on our manuscript, we have revised the paper accordingly. The following responses are provided in detail:

Comments:

(1) Fig 2, Fig S12, and discussion: using concentrations of quantity X (e.g. kg/kg), that are dependent on both source strength of quantity X but also dilution by other terrigenuous or biogenic materials (ie., records of concentration are partly dependent on mass accumulation rate MAR). I picked this up in the previous round (Comment 1 in author responses) but don't feel this has been addressed in the reply. The authors supplied a new figure (stated as X * MAR) but this looks like it's still plotted as conc (X/kg) not flux (X/m2/kyr).

Actually, the authors implicity acknowledge this problem: "Our analysis reveals that the MAR of the studied core closely follows the sedimentation rate and is strongly modulated by variations in biogenic components". If you are plotting a record as X/kg, then surely that record is also strongly modulated in the same way?

So this remains a big weakness from my point of view. For example, at L243: "Meanwhile, the declines in diatom abundance, Si/Al and carbonate content in Section B support a reduction in biogenic components resulting from climatic cooling and ice-sheet expansion (Supplementary Fig. 12), suggesting that decreased biogenic dilution also contributed to the higher magnetite concentration."

The same argument could apply to kaolinte concentration. In Fig S12 both kaolinite content and ARM appear to be strongly negatively correlated with sedimentation rate. Can you really discount the "null hypothesis" that there has been very little long-term change in WAIS glaciation, and instead your ARM & kaolinite records reflect changing dilution? Both could easily be explained by local circulation changes, as could Zr/Rb and Zr/Y.

When the authors then continue: "Therefore, the co-variation of ARM with kaolinite itself serves as a direct tracer of icesheet erosion of this source region, circumventing the uncertainties introduced by secondary calculations", I disagree with this argument. I'm not disputing that that kaolinite and ARM are proxies for ice sheet erosion, but I do dispute that a record confounded by changes in sediment supply can be interpreted as changes in quantity X, when X is the concentration and not the absolute flux. Both the ARM and kaolinite records will be diluted the same amount, so they will of course covary.

The authors have then added (L255) "ARM shows correlations with deep ocean temperature (Tw), sea surface temperature change (ΔSST), benthic δ 256 18O, and sea level (Supplementary Fig. 13), corroborating its relationship with ice-sheet dynamics" I agree this is not inconsisent, but on the other hand it's not strongly supportive either. Very many environmental proxies are well correlated in the Southern Ocean (and globally) at these time scales. Even if the ARM record was not related to ice sheet changes (e.g., if related to circulation-driven changes in deposition rate) it would still very likely be correlated with SST, deep water temp at d18O. Those three variables are of course themselves all very strongly correlated. Or, the correlation could arise because dilution by biogenic material is correlated with SST, sea ice conc, etc.

In summary: the records based on concentrations can be interpreted as a compelling story that supports other evidence for environmental change, but given the ambiguity introduced by dilution I don't think that concentration itself is strong enough for a Nature Comms article. Really you need to convince us that the flux of ASE-sourced sediments has increased at U1532.

Reply:

Thank you for pointing out the limitations in our initial interpretation of the magnetic concentration data. To address this, we have conducted additional opal analyses to evaluate the potential influence of biological dilution, re-examined our dataset with careful consideration of the concentration-versus-flux framework, and implemented substantial revisions throughout the manuscript accordingly.

First, we calculated the fluxes of χ, ARM, SIRM, and HIRM (flux = concentration × sedimentation rate × dry density). HIRM flux represents the flux of high-coercivity minerals, while χ flux and SIRM flux represent the flux of total magnetic minerals. ARM flux represents the flux of fine-grained magnetite. The results show that χ flux, SIRM flux, and HIRM flux all decrease after ~3.2 Ma, closely tracking the sedimentation rate, confirming an overall reduction in terrigenous input, including magnetic minerals. However, ARM flux remains slightly elevated between 3.2 and 2.6 Ma compared to the preceding interval, decoupling from the other magnetic parameters (Fig. 4). This decoupling is consistent with independent magnetic grain-size indicators, which show a fining of the magnetic assemblage after 3.2 Ma and an increased proportion of SD magnetite (Fig. 3). Thus, despite the overall decline in bulk magnetic mineral flux, the supply of fine-grained SD magnetite increased.

Two mechanisms could potentially explain the increase in magnetic concentration despite decreasing flux:

(1) Reduced biogenic dilution. We consider this unlikely to be the dominant mechanism. First, most of our samples are from glacial periods, when sedimentation on the Amundsen Sea continental rise is predominantly terrigenous (Line 186-195). Productivity proxy Ba/Al show little change across the interval (Fig. 4). To quantitatively assess dilution, we analyzed opal content in 53 additional samples. The results show consistently low opal values (<8%) throughout the studied interval. Opal content, combined with shipboard measurements of $CaCO_3$ and total organic carbon (TOC), decreased by 3.62%

from Section C to Section B, yielding a theoretical maximum dilution contribution of only ~3.9% to ARM (Supplementary Figure 12). In contrast, the observed ARM increase is 200%, demonstrating that biogenic dilution cannot be the dominant cause of the magnetite enrichment (Supplementary Fig. 12). Furthermore, opal content exhibits no correlation with magnetic parameters (Fig. R1). When magnetic concentration parameters are normalized to conservative terrigenous elements (Al, Ti), the resulting ratios (ARM/Al, ARM/Ti, etc.) increase after 3.2 Ma (Supplementary Fig. 13), mirroring the raw concentration data (Supplementary Fig. 16). Normalization to terrigenous elements eliminates dilution effects from both biogenic and other terrigenous materials. This confirms that the observed magnetic enrichment reflects a true increase in magnetite within the terrigenous fraction, not an artifact of changing dilution. Additionally, the normalized magnetic parameters show a strong correlation with the raw concentration data (Supplementary Fig. 14), indicating that the raw data are largely unaffected by variations in dilution from other components over long-term trends.

(2) A change in sediment provenance. Multiple independent lines of evidence converge on the Thwaites Glacier (TG) catchment as the source of this magnetite enrichment. First, kaolinite content, measured as a relative percentage within the clay mineral assemblage after removal of biogenic and non-clay components, is immune to the same dilution effects that affect bulk concentrations and serves as a provenance tracer (Ehrmann et al., 2011; Hillenbrand et al., 2003). Second, surface sediment studies in the Amundsen Sea have shown that detritus from the TG catchment and western Amundsen is characterized by higher detrital ε_{Nd}, higher kaolinite content, higher Zr/Y ratios, and a higher abundance of mafic minerals compared to the Pine Island Glacier (PIG) catchment (Simões Pereira et al., 2018, 2020; Fig. R2). At Site U1532, detrital ε_{Nd} (Rahaman et al., 2025; Horikawa et al., 2026), kaolinite content (Gohl et al., 2021; Passchier et al., 2025), and Zr/Y ratios all increase after 3.2 Ma and remain elevated through the Pleistocene (Fig. R3), synchronous with the magnetic enrichment. The convergence of multiple independent proxies, each sensitive to different aspects of source rock composition, provides compelling evidence for a provenance shift. Notably, among all magnetic parameters, ARM shows the strongest correlations with detrital ε_{Nd}, kaolinite, and Zr/Y (Supplementary Fig. 15, Fig. 5), indicating that SD magnetite dominates the magnetic assemblage of this newly sourced provenance. ARM shows consistent long-term trends with ARM/Al and ARM/Ti (Supplementary Fig. 16). Critically, recent aerogeophysical surveys have revealed a zone of Cretaceous mafic magmatism extending ~200 km inland from the coast beneath the Thwaites Glacier catchment (Jordan et al., 2023; Fig. R4). Such mafic rocks are characterized by high content of magnetic minerals, providing a direct geological source for the magnetite-rich detritus deposited at Site U1532. Meanwhile, sedimentary basins situated 150-200 km inland of the coast, covering 20% of the TG catchment (Jordan et al., 2023; Fig. R4), are a potential source of kaolinite (Passchier et al., 2025; Ehrmann et al., 2011). Although direct ε_{Nd} values of subglacial bedrock beneath Thwaites Glacier are lacking due to limited exposure, surface sediment data show elevated values near the glacier (Fig. R2). The mafic intrusions formed during West Antarctic Rift System extension (Jordan et al., 2023; Borthwick et al., 2025) and belong to the Marie Byrd Land tectonic domain (Mukasa & Dalziel, 2000). Coeval mafic intrusive rocks (113-98 Ma) in the Fosdick Mountains yield ε_{Nd}

values of -1.6 to +2.5, interpreted to reflect derivation from a metasomatized, hydrous mantle source modified by subduction processes (Saito et al., 2013). Given the shared tectonic setting and magmatic affinity, the mafic intrusions beneath Thwaites Glacier likely exhibit ε_{Nd} values within a comparable range—predominantly positive to weakly positive—and thus contributed to the observed ε_{Nd} increase after 3.2 Ma. Furthermore, Passchier et al. (2025) showed that the sedimentary composition on the Amundsen Sea continental rise, based on early Pliocene samples from Site U1532, is particularly tied to the dynamics of Thwaites Glacier.

Additionally, direct erosion of fresh mafic bedrock by ice sheets is generally difficult due to the high mechanical strength and low joint density of these rocks. During ice-free periods, these mafic rocks could undergo weathering at the surface, which would generate mafic debris that is subsequently mixed with other sediment sources, such as the adjacent sedimentary basin fill (Vogel et al., 2006). When ice sheets subsequently expanded, they preferentially eroded these pre-weathered, mechanically weaker sedimentary horizons rather than the intact mafic bedrock. The resulting glacial sediments, containing recycled weathering products, were then transported to our sampling locations. This mixed material was then transported to the sampling site by glacial processes. In this case, the sediments may be enriched in both magnetite and kaolinite.

In summary, the post-3.2 Ma increase in ARM records the seaward expansion of the WAIS, particularly Thwaites Glacier, and the progressive erosion of this inland mafic and sedimentary basin province. The correlations between ARM and global climate proxies (deep-ocean temperature, sea level, etc.) therefore likely reflect the natural coupling between WAIS dynamics and global environmental change.

Based on the above considerations, we have thoroughly revised the "Magnetite tracks ice sheet dynamics" section (Lines 185-291) of the Discussion.

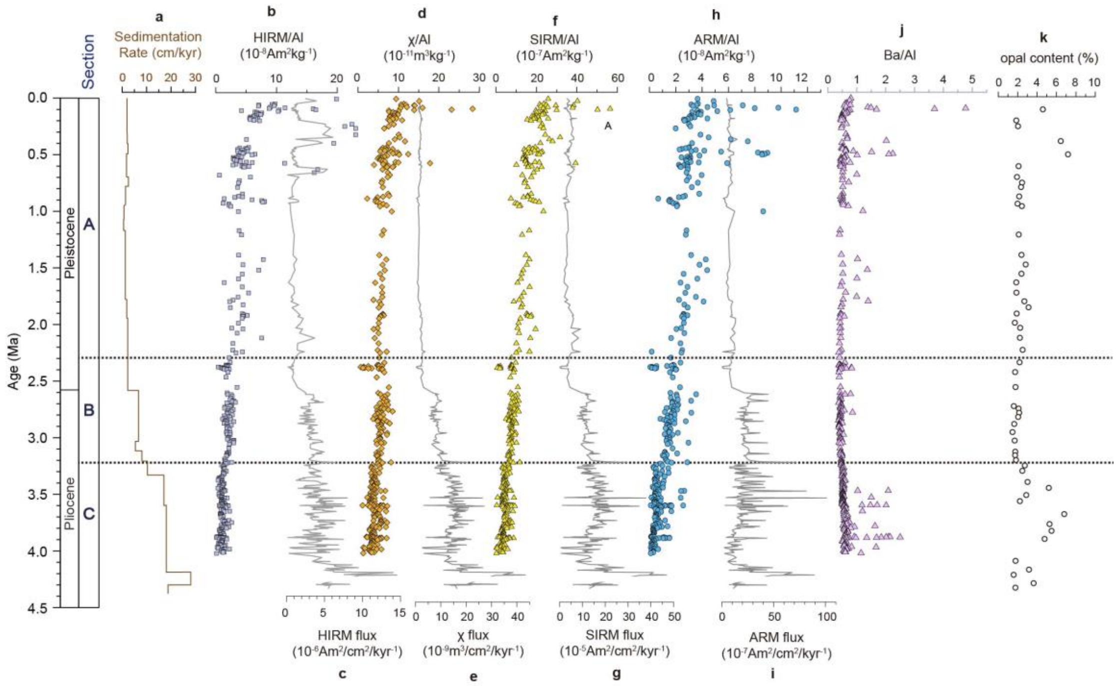

Fig. 4 | Terrigenous and biogenic input variations since ~4.33 Ma at Site U1532. (a) Sedimentation rates. (b) HIRM/Al. (c) HIRM flux. (d) χ/Al. (e) χ flux. (f) SIRM/Al. (g) SIRM flux. (h) ARM/Al. (i) ARM flux. (j) Ba/Al, indicative of paleoproductivity. (k) Opal content. The flux of magnetic minerals was obtained by multiplying the environmental magnetic parameters by the sedimentation rate and dry density. HIRM flux represents the flux of high-coercivity minerals, while χ flux and SIRM flux represent the flux of total magnetic minerals. ARM flux represents the flux of fine-grained magnetite.

Component	Section C average (pre-3.2 Ma)	Section B average (post-3.2 Ma)	Change
**Opal**	3.53%	1.88%	-1.65%
**$CaCO_3$**	2.25%	0.23%	-2.02%
**TOC**	0.20%	0.25%	+0.05%
**Biogenic material**	~5.98%	~2.36%	-3.62%
**Terrigenous (balance)**	~94.02%	~97.64%	+3.62%

Relative increase in terrigenous fraction:

- Section C: 94.02%
- Section B: 97.64%
- Relative increase = (97.64 - 94.02) / 94.02 = **3.62% / 94.02 ≈ 3.85%**

Theoretical maximum contribution to ARM:

- ARM is derived from the terrigenous fraction, and assuming that the ARM intensity of the terrigenous fraction remains constant
- Then ARM concentration is proportional to the terrigenous proportion
- Maximum theoretical ARM increase = **3.85%**

Observed ARM increase:

- Section C mean ARM: 1×10^{-4} Am^2/kg
- Section B mean ARM: 3×10^{-4} Am^2/kg
- Observed increase = (3 - 1) / 1 × 100% = **200%**

Comparison:

- Theoretical maximum dilution contribution: **3.85%**
- Observed ARM increase: **200%**
- Observed increase exceeds the theoretical maximum by a factor of **>50**

Supplementary Figure 12. Calculation of influence on ARM by biogenic dilution effect. Calcium carbonate and total organic carbon (TOC) contents were obtained from shipboard measurements.

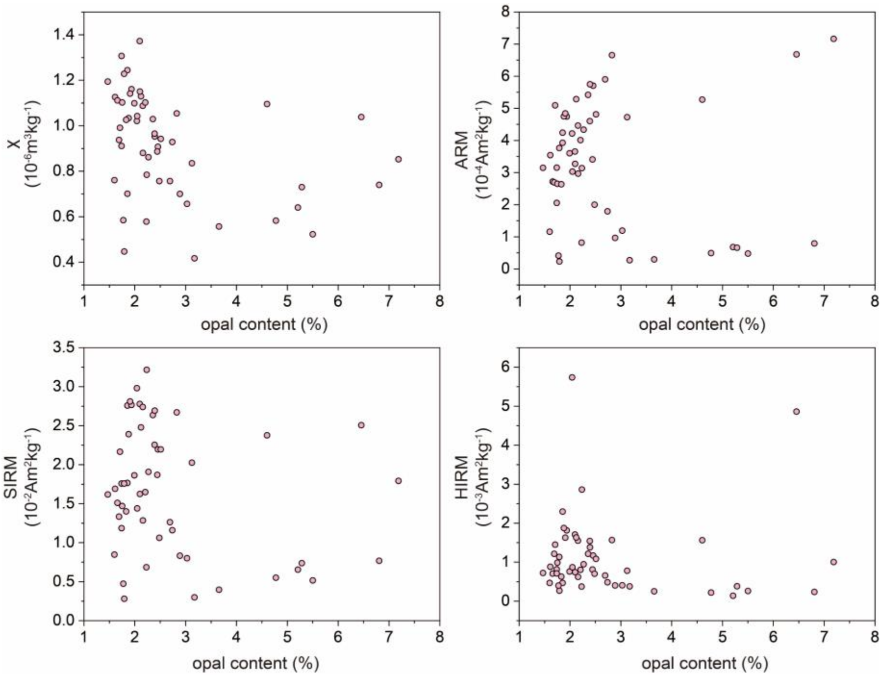

Fig. R1 Bi-plots of opal content vs. magnetic-concentration-related parameters.

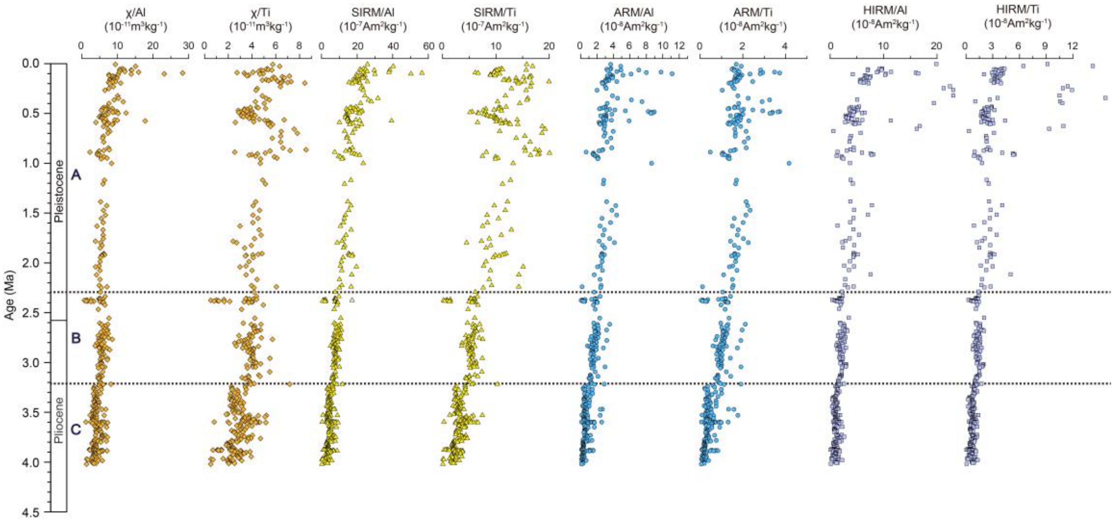

Supplementary Figure 13. Normalized concentration parameters to conservative terrigenous elements Al and Ti.

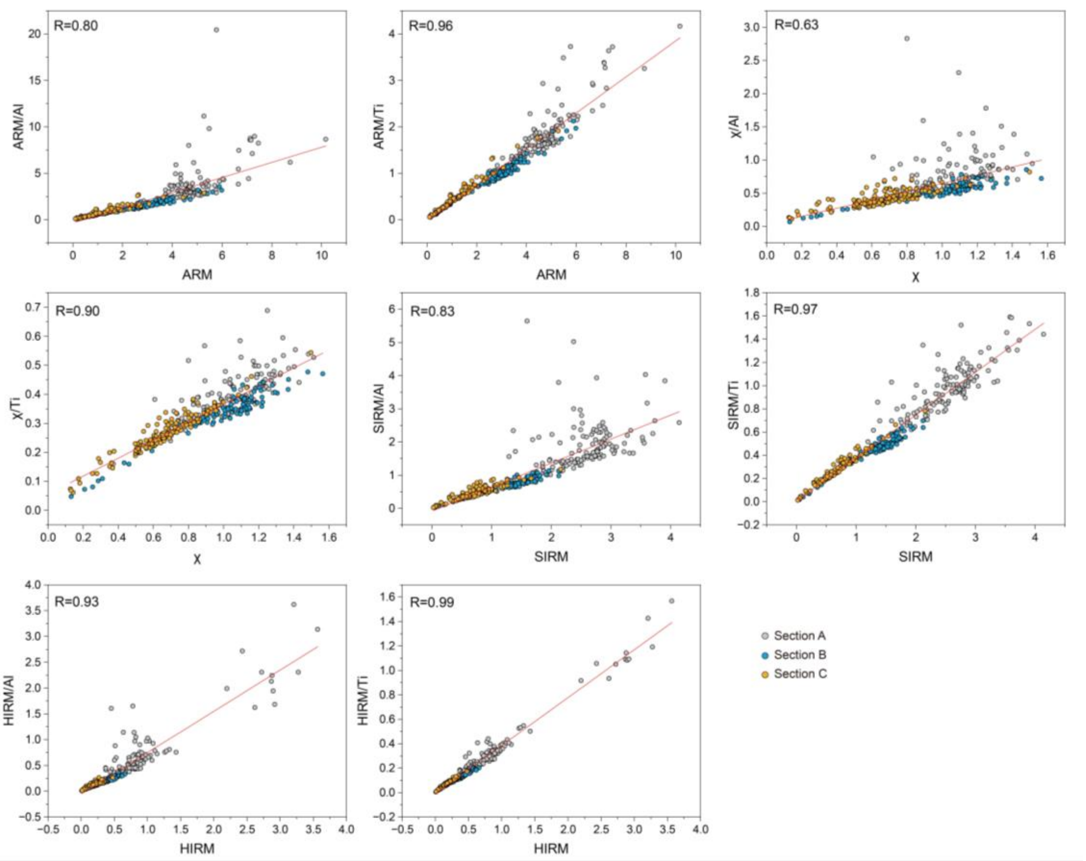

Supplementary Figure 14. Correlation of normalized concentration parameters with conservative terrigenous elements Al and Ti.

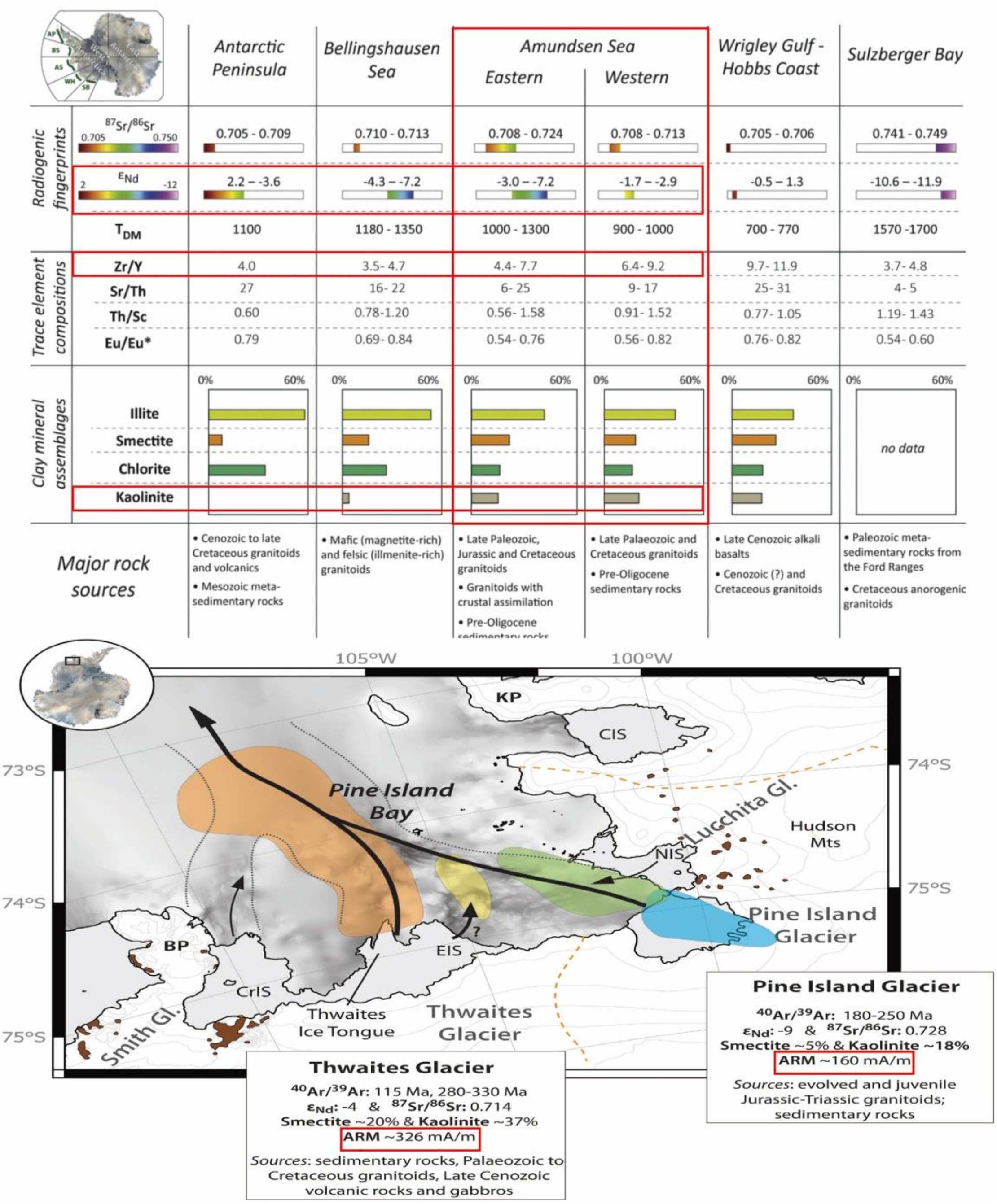

Fig. R2. Distribution characteristics of surface sediments in the Amundsen Sea.

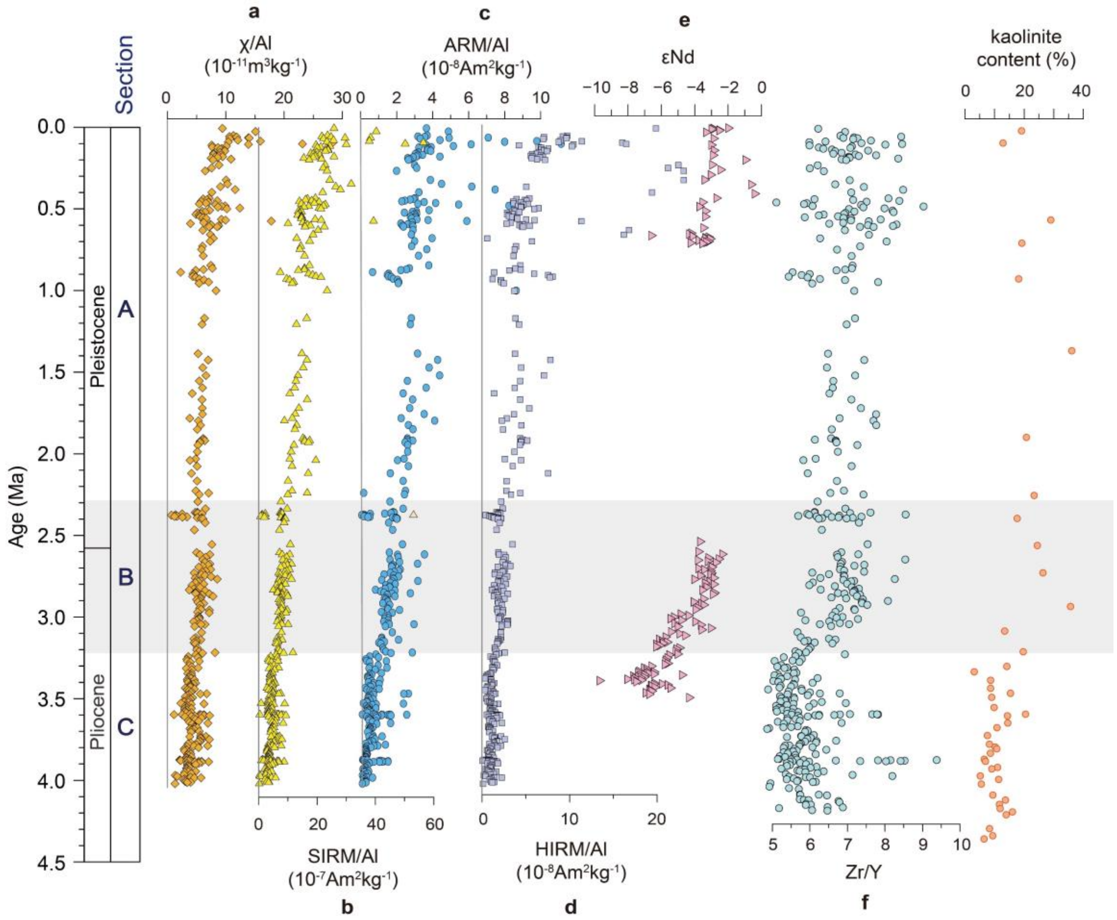

Fig. R3. Variations in magnetic and provenance proxies at Site U1532 since 4.33 Ma.

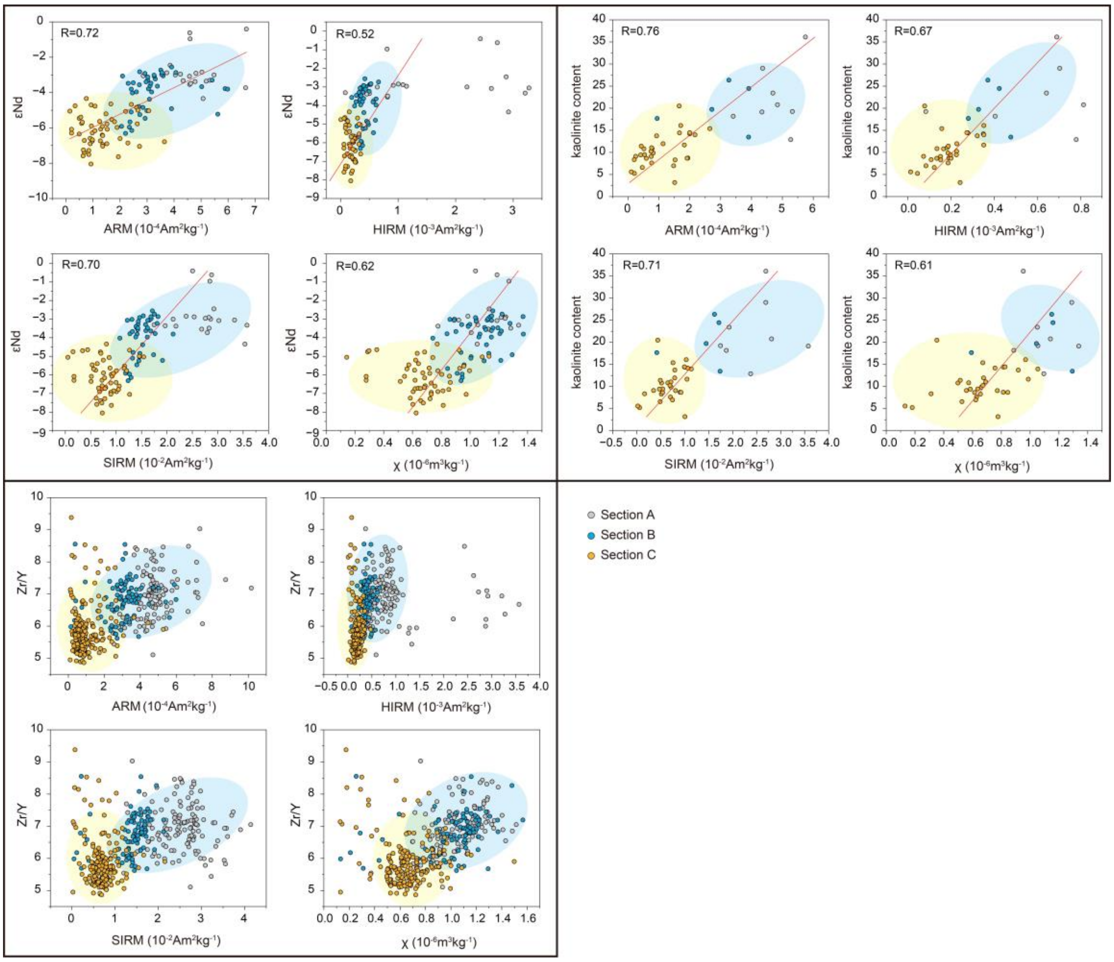

Supplementary Figure 15. Correlation of magnetic concentration-related parameters with detrital Nd, kaolinite content and Zr/Y. Yellow shading denotes the range of most data points before 3.2 Ma, and blue shading denotes the range after 3.2 Ma. Kaolinite content data is from[16]. Data of detrital ε_{Nd} is from[2,17].

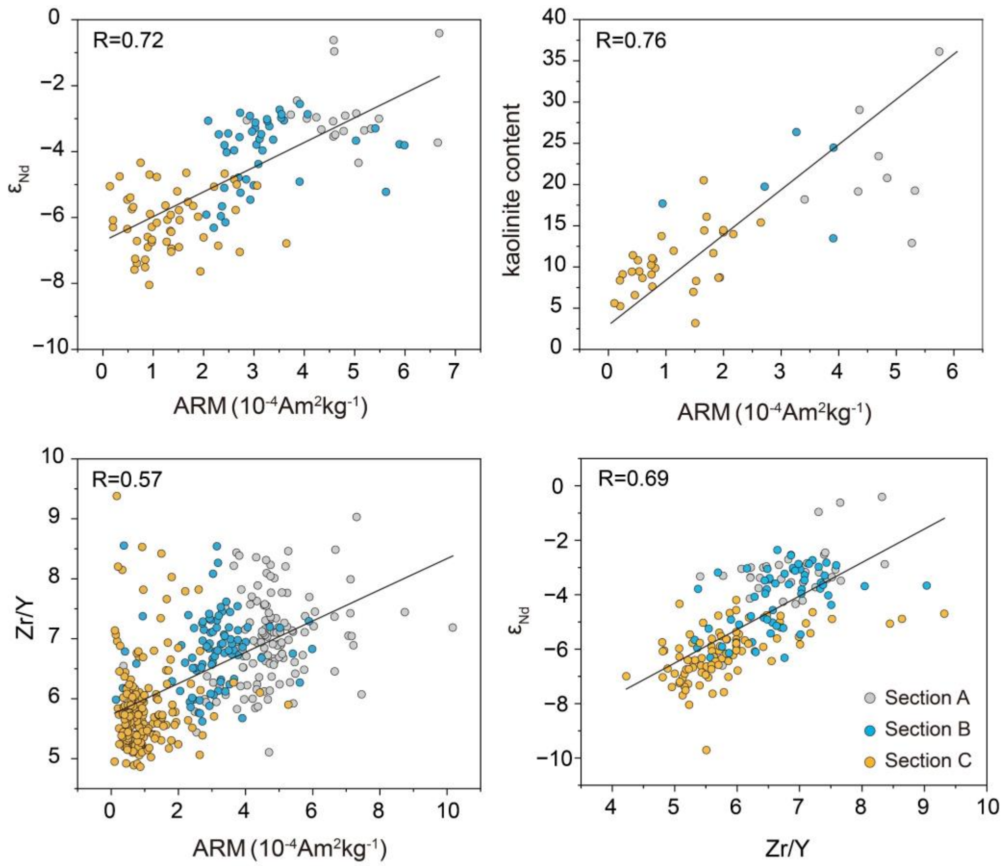

Fig. 5 | Variations in terrigenous sediment composition. (a) biplot of ARM vs. detrital ε_{Nd}; (b) ARM vs. kaolinite content; (c) ARM vs. Zr/Y; (d) Zr/Y vs. ε_{Nd}. R: Pearson's correlation coefficient. Yellow symbol: section C; blue symbol: section B; gray symbol: section A. Data of detrital ε_{Nd} is from[39,43].

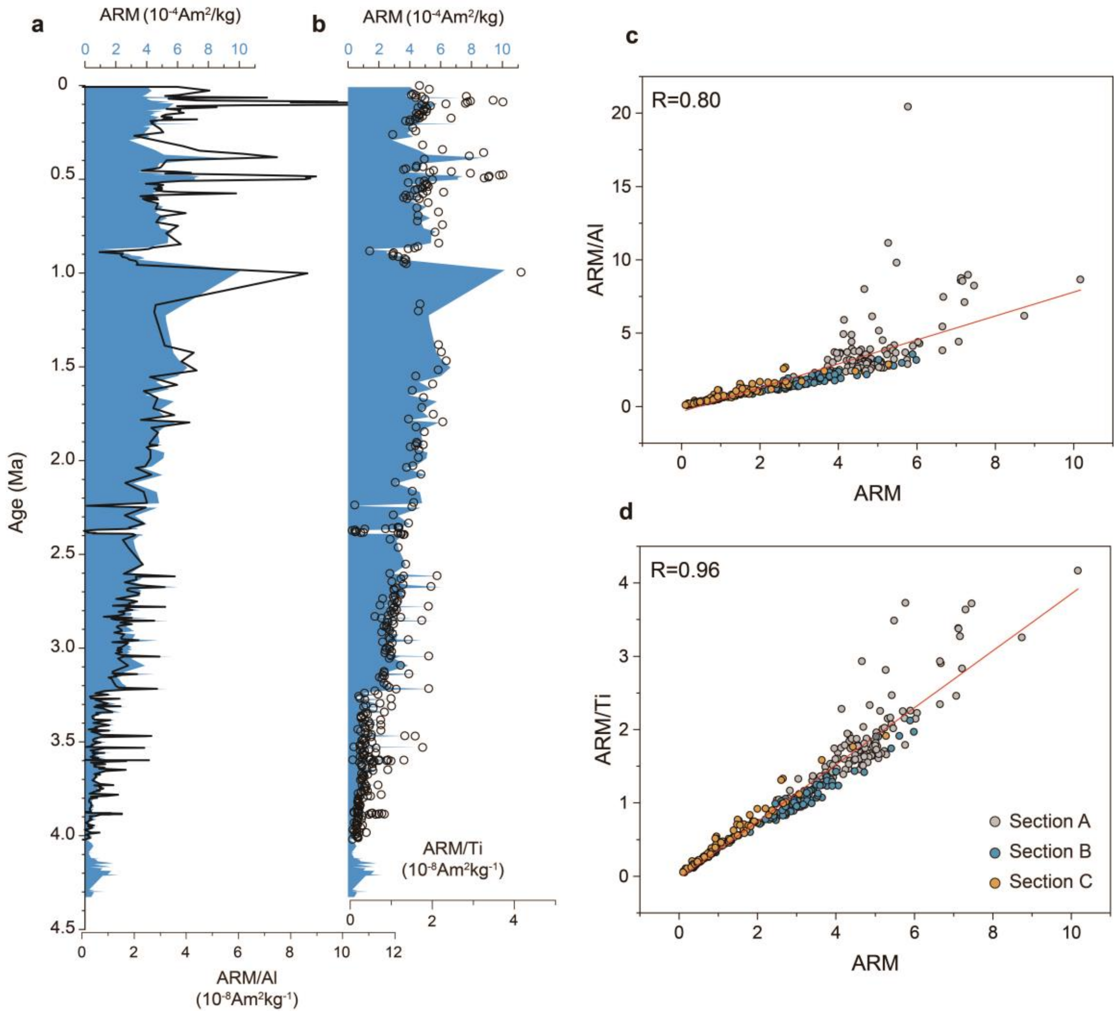

Supplementary Figure 16. Consistent long-term trends between ARM and terrigenous-normalized magnetic parameters (ARM/Al, ARM/Ti). (a) Comparison of ARM (blue shading) and ARM/Al (black line). (b) Comparison of ARM (blue shading) and ARM/Ti (black circles). (c) Bi-plot of ARM vs. ARM/Al. (d) Bi-plot of ARM vs. ARM/Ti. R: Pearson's correlation coefficient.

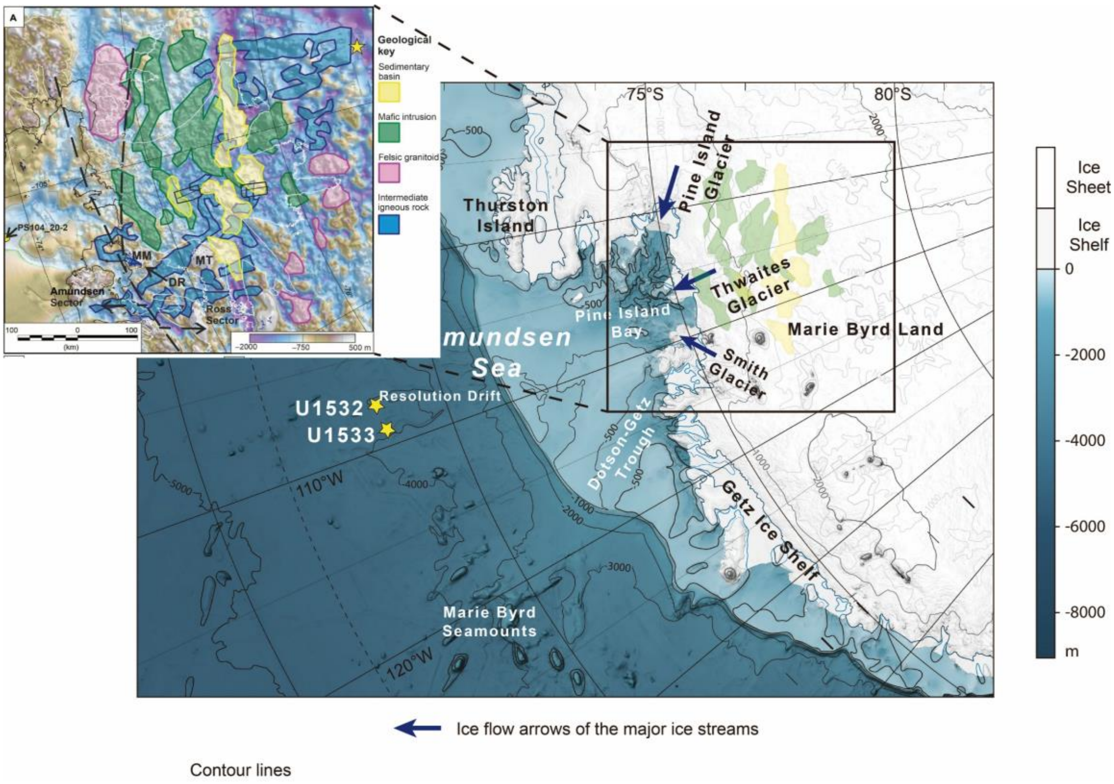

Fig. R4. Mafic intrusion under Thwaites Glacier. The geological map in the upper left is from Jordan et al. (2023).

(2) L237 "The distribution and abundance of preserved grounded-ice wedges from the Pliocene to the Pleistocene indicate repeated ice-sheet advances to the shelf edge during the Pliocene, followed by limited further progression in the Pleistocene [Ref37]". Can you build on the conclusions of Ref 37 Gohl et al 2021? I'm now wondering what can we confidently learn from the U1532 data, that was not evident from that finding in Gohl et al. 2021? Great that your results here support that earlier work, but are the grounding zone wedges combined with subsurface ocean T already more compelling than the U1532 record (given Comment 1 above)?

Reply:

Gohl et al. (2021) provided two key insights from seismic stratigraphy: (1) the Amundsen Sea shelf grew oceanward by ~80 km during the Pliocene due to repeated advances of grounded ice, indicating highly dynamic WAIS behavior; and (2) multiple buried grounding zone wedges on the outer shelf, separated by thick sediment drapes, suggest that major WAIS advances were absent during many orbital cycles between 4.2 and 3.2 Ma, a period they termed the Pliocene Amundsen Sea Warm Period (PAWP), consistent with elevated diatom abundance. Gohl et al. (2021) did not, however, discuss ice-sheet dynamics after 3.2 Ma, though they noted a seaward shift of the shelf break in the Pleistocene relative to the Pliocene, as inferred from shelf-break formations.

Our magnetic record from Site U1532 is consistent with and extends these findings. The interval of low magnetic mineral concentration during 4.1-3.2 Ma supports the PAWP proposed by Gohl et al. (2021). More importantly, magnetic data provide new insights into WAIS behavior after 3.2 Ma that cannot be obtained from seismic stratigraphy alone. We observe a decoupling between decreasing terrigenous flux and increasing magnetic mineral concentration after 3.2 Ma, accompanied by a fining of magnetite grain size (Figs. 2-4). The synchronous increase in SD magnetite with kaolinite, detrital ε_{Nd}, and Zr/Y, combined with aerogeophysical evidence of Cretaceous mafic belt and sedimentary basins beneath Thwaites Glacier (Jordan et al., 2023), reveals a progressive increase in sediment contribution from the Thwaites Glacier catchment after 3.2 Ma. This provenance shift records the seaward expansion of the WAIS into this mafic province, a level of source-specific detail that seismic data alone cannot provide.

While seismic stratigraphy can document that ice advances occurred and provide a structural framework, it cannot constrain the precise timing of these advances beyond the resolution of available age tie points, nor can it identify the specific source regions being eroded, or the continuity of erosion between advances. In contrast, continuous, high-resolution, age-constrained magnetic record offers complementary insights: it allows precise timing of provenance shifts linked to global climate events. When combined with subglacial bedrock drilling, this approach can help constrain ice-sheet extent. Together, the seismic and magnetic approaches provide a more complete picture of WAIS evolution.

(3) Cross shelf transport

L184 "Terrigenous sedimentation on the Antarctic continental rise is controlled by glacial processes that generate glaciogenic detritus on land and the continental shelf, which is then transported downslope via gravity flows—such as slumps and turbidity currents—through deep-sea channels to the continental slope and rise. This process occurs primarily when the ice sheet advances onto the continental shelf during glacial periods. Sedimentary facies analysis of Expedition 379 cores indicates that terrigenous debris was primarily delivered to the continental rise by glacial-advance gravity flows and deglacial hyperpycnal plumes generated while the grounding line remained near the shelf break and meltwater discharge intensified"

Here I think is an important point to make, that under current conditions (maybe at least Pleistocene) the inland sloping shelf means that the downslope transport of sediments off the continental shelf occurs only when the ice advances to the shelf edge. See BedMachine plot below. Note that in Fig 1, an arrow showing "downslope" sediment transfer is pointing uphill away from the grounding line. It seems like a minor point but I think is an important part of the story. If as the authors suggest in their reply that "Sediments may be routed through alternative, adjacent channels" this opens up other ambiguities as to how the concentration signal at U1532 will likely be modified by changes in transport characteristics along a less direct pathway, presenting further difficulties with interpreting the record.

Reply:

We agree that downslope transport of terrigenous sediment to the continental rise predominantly occurs when the ice sheet advances to the shelf edge. The arrow in Fig. 1 was intended only as a schematic illustration of sediment dispersal and has been removed from the revised manuscript to avoid any confusion about specific transport pathways.

This is described in the "Drill site description" subsection of the Methods:

Line 389-395: *"Sediments retrieved at Site U1532 are mostly of terrigenous origin, with occasional occurrences of pelagic or hemipelagic components, including microfossils. Lithofacies cyclicity was observed along the U1532 core. Thin greenish (in the uppermost part of the site brownish) muddy units were characterized by the presence of siliceous microfossils, bioturbation, and ice-rafted debris (IRD). These greenish units have been interpreted to represent interglacial deposits. They alternate with thick, gray laminated, predominantly terrigenous silty-clay units interpreted to represent glacial periods."*

The majority of our samples were collected from the gray layers, representing glacial intervals.

More importantly, multiple lines of evidence indicate that sediment transport processes did not significantly modify the provenance signal recorded at Site U1532. The strong correlation between magnetic parameters and independent provenance tracers, including detrital ε_{Nd}, kaolinite content, and Zr/Y ratios, suggests that these parameters share a common source control rather than being independently modulated by transport.

Thus, while the exact pathways of downslope transport are complex and difficult to reconstruct, the multi-proxy provenance dataset provides confidence that the sediment reaching Site U1532 reliably records the composition of its source region in the ASE hinterland.

(4) Sources of kaolinite and magnetic minerals

The kaolinte and magnetic minerals are proposed to derive from on-shelf pre-Oligocene strata (L205 "The content of clay mineral kaolinite is generally associated with chemical weathering of rock in warm and humid climates, and it has been suggested that pre Oligocene sedimentary strata on the ASE shelf and its hinterland are the sources for the relatively high kaolinite contents found in the offshore sediments of the Amundsen Sea" … then later L224 "the magnetic minerals in the sediments at Site U1532 are mainly derived from the pre-Oligocene sedimentary strata on the ASE shelf.")

I'm not disputing the provenance analysis, but the logic later is not entirely convincing.

The above statements seem sound on the basis that the pre-Oligocene strata were eroded by overlying ice, transported subglacially to the shelf break, and transported downslope to U1532 by gravity currents/slumping. However, the increase in kaolinte content and ARM since 3.2 Ma is not immediately consistent with the model proposed in Fig 5, where the Pliocene-Pleistocene ice sheet advance after 3.2 Ma is attributed to increasing stability provided by increasing sediment thickness on the shelf. That

deposited sediment will reduce erosion of underlying rocks.

Are you suggesting the pre-Oligocene strata were eroded by the smaller Pliocene ice sheet, resulting sediments were trapped on the shelf, and were then reworked / transported subglacially to shelf edge during subsequent late-Pliocene advance? That explains the initial increase in kaolinite, but given that Pleistocene sediments eventually cover the Pliocene sediments (top panel in Fig 5), where does the continued supply of kaolinite come from in the Pleistocene? Is it from further inland, then transported outwards episodicallly during glacial maxima?

This part of the interpretation and Fig 5 I think needs looking at carefully or rewording to make sure it's not contradictory (and make sure is consistent with the kaolinite & ARM flux records so we're not falsely building this interpretation on a record of dilution).

Reply:

Thank you for pointing out the ambiguity in our previous description.

After re-examining our data, we confirm that the terrigenous flux at Site U1532 indeed decreased after 3.2 Ma, as noted by the reviewer. Following your suggestion, we have revised our interpretation accordingly.

First, we conducted opal analyses to test whether the increase in magnetic concentration could be explained by reduced dilution from biogenic components. Moreover, normalizing the magnetic parameters to conservative terrigenous elements (Al, Ti) yields ratios that represent the magnetic mineral content per unit of terrigenous detritus, independent of dilution. These ratios show a clear increase after 3.2 Ma, mirroring the raw magnetic data, confirming that the magnetite enrichment is intrinsic to the terrigenous fraction.

Second, we find that the post-3.2 Ma increase in magnetite concentration, accompanied by a fining of magnetic grain size, coincides with synchronous increases in independent provenance proxies: kaolinite content, detrital ε_{Nd} values, and Zr/Y ratios. This convergence of multiple proxies points to a change in sediment source rather than a dilution effect. Environmental magnetic parameters are widely used to track sediment provenance through variations in magnetic mineral concentration, assemblage, and grain size, and our data are consistent with such an interpretation.

Accordingly, we have refined our depositional model. We propose that as the WAIS expanded seaward after 3.2 Ma, it progressively eroded a distinct geological source region characterized by high SD magnetite concentrations and high kaolinite content. Previous provenance studies have shown that sediments delivered by Thwaites Glacier exhibit these characteristics (Simões Pereira et al., 2018, 2020). While direct bedrock samples from beneath the ice are not available, aerogeophysical surveys have revealed a Cretaceous mafic magmatic belt and sedimentary basins extending ~200 km inland beneath Thwaites Glacier (Jordan et al., 2023), providing independent geophysical support for the presence of such a source region.

Regarding previous Fig. 5, we apologize for the confusion. This figure was adapted from the seismic profiles of Gohl et al. (2021) and was intended only to illustrate the advance of the ice sheet margin (i.e., decreasing transport distance to the core site), not to imply that the sediment source was on the shelf itself, or ice sheet advance after 3.2 Ma was attributed to increasing stability provided by increasing sediment thickness on the shelf. The orange line in Fig. R5 marks the location of the seismic line from Gohl et al. (2021), which served as the basis for the schematic diagram in our previous version. As shown in Fig. R6 of Gohl et al. (2013), Pliocene and Pleistocene sediment cover exists only on the outer shelf and therefore does not cover the provenance area beneath Thwaites Glacier. The outer shelf, which is overlain by Pliocene and Pleistocene sediments, serves as a depositional area receiving material transported from further inland. These outer shelf strata are continuous with the sedimentary sequence at Site U1532 (Fig. R5). We therefore redrew the figure to illustrate the changes in the erosion zone with ice sheet growth in Pleistocene (Supplementary Fig. 17).

Therefore, following the revisions made in response to Comment 1, the sedimentary pattern revealed by the post-3.2 Ma increase in magnetite, kaolinite, detrital ε_{Nd}, and Zr/Y indicates progressive erosion of the bedrock beneath Thwaites Glacier as the WAIS advanced.

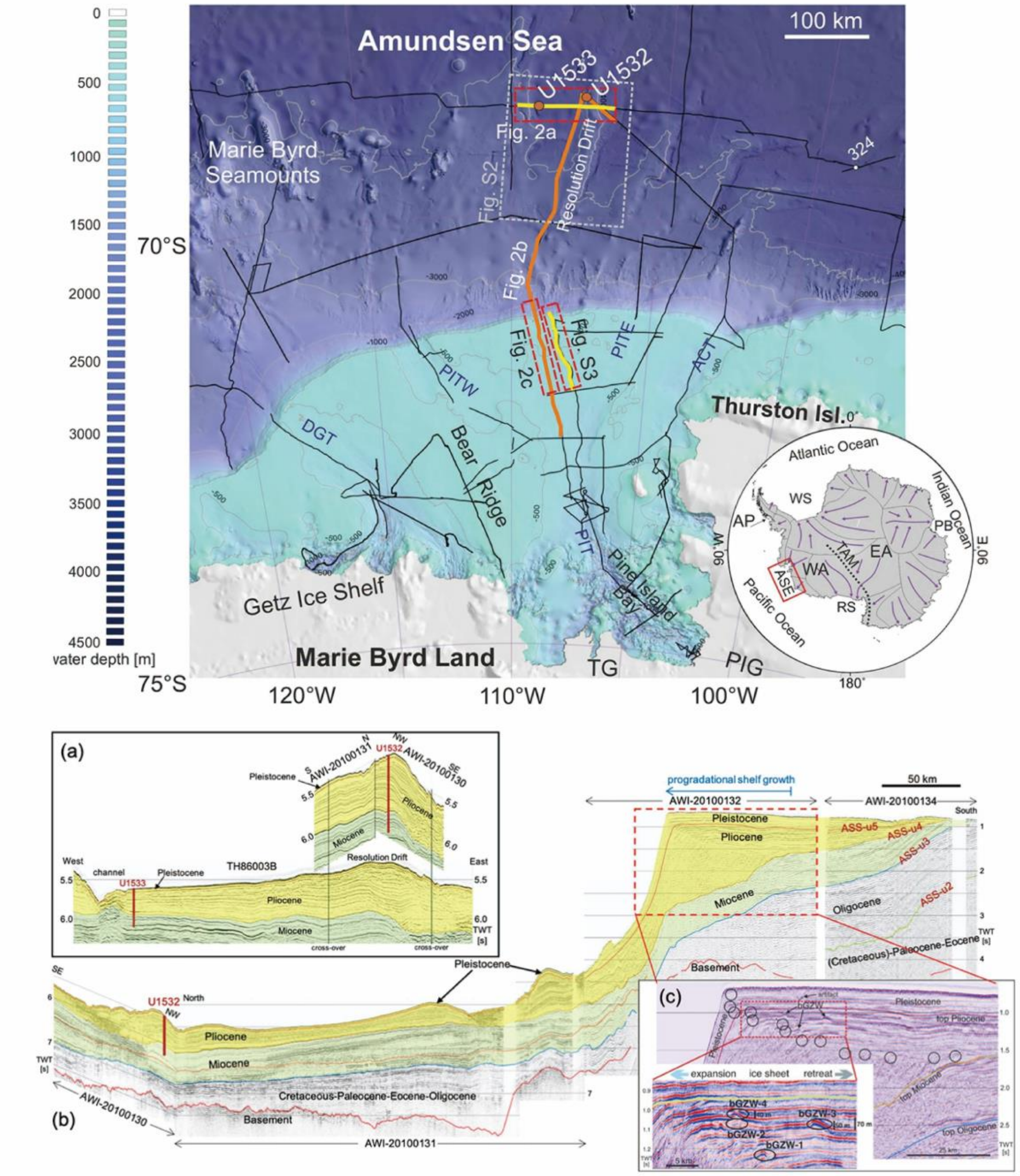

Fig. R5. Seismic transect from Site U1532 on the rise to the outer shelf.

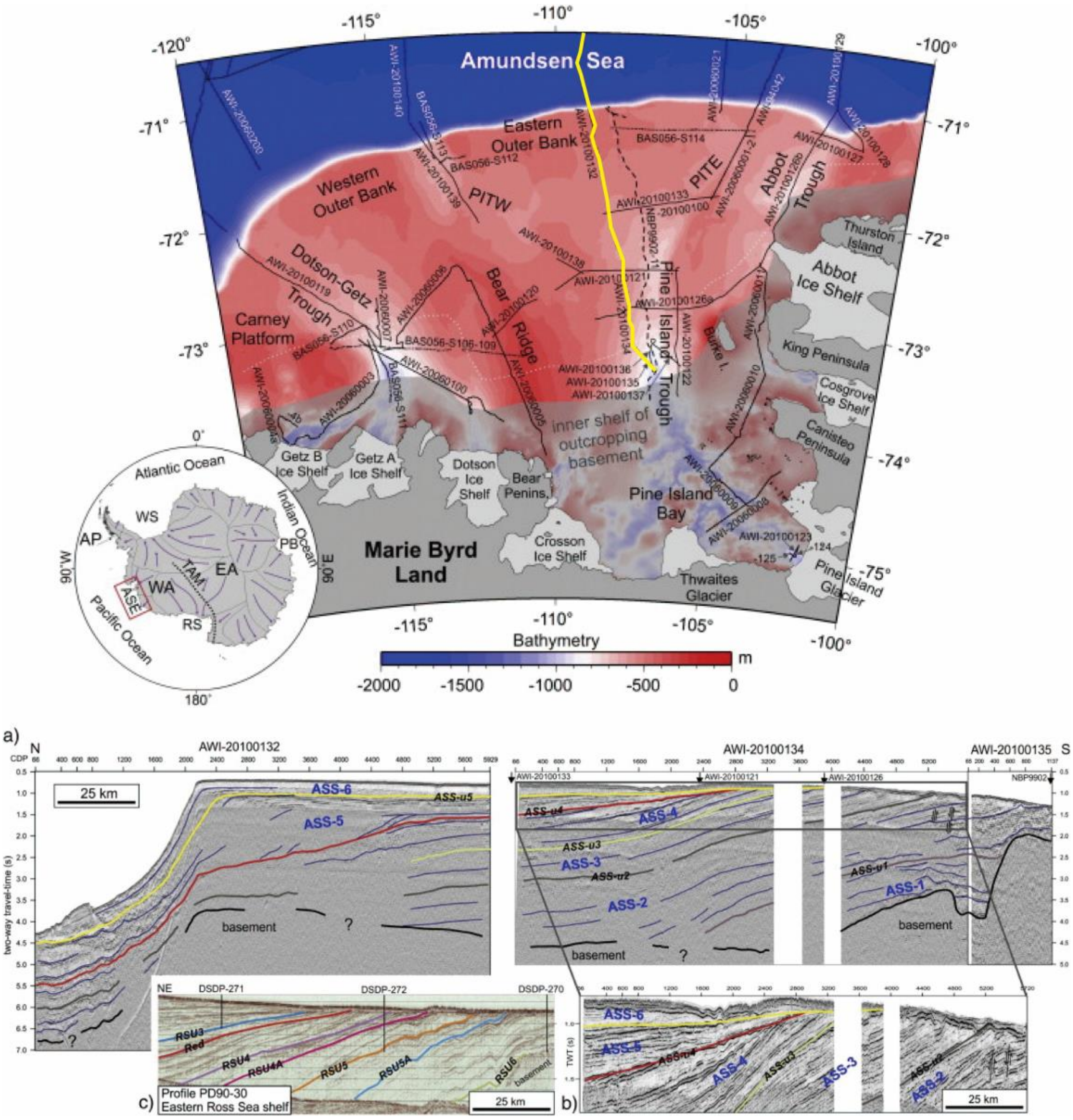

Fig. R6. Composite of seismic profiles from the central Pine Island Trough across the outer shelf and slope of the eastern ASE shelf.

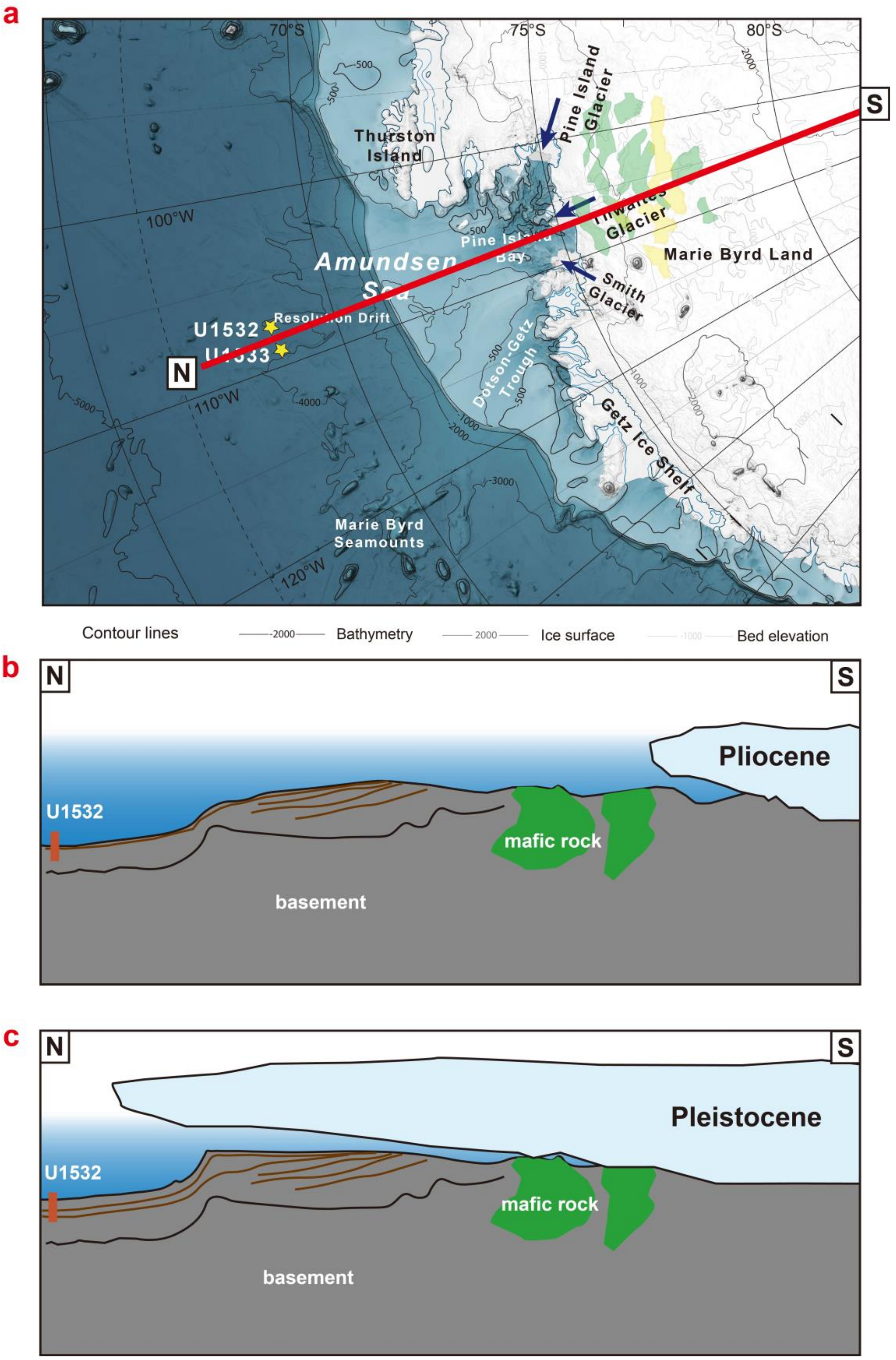

Supplementary Figure 17. Schematic diagram of the change in ice-sheet growth and erosion patterns from the Pliocene to the Pleistocene. (a) A regional map of the Amundsen Sea Embayment (ASE) and

coastal Marie Byrd Land (MBL). Resolution Drift in the Amundsen Sea sector and the drill sites (U1532 and U1533, yellow stars) of the International Ocean Discovery Program (IODP) Expedition 379 are shown. Bathymetry is from International Bathymetric Chart of the Southern Ocean (IBCSO) v2[18]. Blue arrows indicate the ice flow directions of the major ice streams. Green shaded areas represent mafic intrusive rocks, and yellow shaded areas represent sedimentary basins, based on Jordan et al.[19]. The red line delineates the extents of the schematic diagrams in (b) and (c). (b) A smaller ice sheet in the Pliocene. (c) An advanced ice sheet in the Pleistocene. The larger Pleistocene ice sheet eroded the mafic intrusive rocks and sedimentary basins beneath Thwaites Glacier, transporting substantial magnetite to Site U1532.

Minor points:

L258 "Despite typically higher sedimentation rates during glacial periods due to enhanced terrigenous input, a long-term decrease in rates accompanied ice advance after ~4.1 Ma (Fig. 2a). Decreased glacial erosion rates resulting from colder, drier subglacial conditions may contribute to the decreased sedimentation rates associated with cooling (Fig. 2a)"

Very speculative and the cited reference (#49) alludes to the Antarctic Peninsula and Patagonia, not WAIS. If I wanted to speculate on ASE ice stream dynamics I would put my money on these being active, warm-based fast ice flow features throughout even the coldest glacial periods, with subglacial heating/melting sustained by frictional heating and localised strain heating as is the case today. If the ice streams shut down (as implied by colder subglacial conditions, which would switch off sliding) you would effictively shut down nearly all dynamic ice loss from this catchment.

Reply:

Thank you for this insightful comment. We agree that invoking colder, drier subglacial conditions to explain the long-term decrease in sedimentation rates is speculative and not well supported by the cited reference, which focuses on the Antarctic Peninsula and Patagonia rather than the WAIS. We also acknowledge your point that the Amundsen Sea sector ice streams are likely warm-based and active even during cold glacial periods, sustained by frictional and strain heating. Therefore, this statement has been removed from the revised manuscript.

In its place, we cited Rahaman et al. (2025): as the WAIS expanded to its near-modern size during the Plio-Pleistocene transition, glacial-interglacial fluctuations became confined to the marine realm, reducing erosion of continental bedrock and thus lowering total sediment supply. Additionally, glacial over-deepening and the higher erosion resistance of mafic rocks may have also contributed to the decrease in sedimentation rate (Line 275-285).

L302 “Atmospheric CO_2 concentrations, ocean temperature and ocean currents are proposed to be the key factors influencing WAIS variability”

CO_2 influences WAIS stability through its influence on ocean temp & circulation. Could reword something like “Atmospheric CO_2-driven changes in ocean temp/circulation are proposed as key factors… etc”

Reply:

This sentence has been rephrased as recommended.

Line 311-312: *“Atmospheric CO_2-driven changes in ocean temperature and circulation are proposed as key factors influencing WAIS variability.”*

WAIS advance since the early Pliocene

Zhang et al.

I'd like to thank the authors for taking the time to reply to my comments on the previous version. I'm happy with most of the replies and corresponding changes. However there are some remaining points that I think are important as they're central to this paper, but have not been adequately resolved. It would be great if you could revisit these.

(1) Fig 2, Fig S12, and discussion: using concentrations of quantity X (e.g. kg/kg), that are dependent on both source strength of quantity X but also dilution by other terrigenuous or biogenic materials (ie., records of concentration are partly dependent on mass accumulation rate MAR). I picked this up in the previous round (Comment 1 in author responses) but don't feel this has been addressed in the reply. The authors supplied a new figure (stated as X * MAR) but this looks like it's still plotted as conc (X/kg) not flux (X/m2/kyr).

Actually the authors implicity acknowledge this problem: "Our analysis reveals that the MAR of the studied core closely follows the sedimentation rate <u>and is strongly modulated by variations in biogenic components</u>". If you are plotting a record as X/kg, then surely that record is also strongly modulated in the same way?

So this remains a big weakness from my point of view. For example at L243: "*Meanwhile, the declines in diatom abundance, Si/Al and carbonate content in Section B support a reduction in biogenic components resulting from climatic cooling and ice-sheet expansion (Supplementary Fig. 12), suggesting that decreased biogenic dilution also contributed to the higher magnetite concentration.*"

The same argument could apply to kaolinte concentration. In Fig S12 both kaolinite content and ARM appear to be strongly negatively correlated with sedimentation rate. Can you really discount the "null hypothesis" that there has been very little long-term change in WAIS glaciation, and instead your ARM & kaolinite records reflect changing dilution? Both could easily be explained by local circulation changes, as could Zr/Rb and Zr/Y.

When the authors then continue: "*Therefore, the co-variation of ARM with kaolinite itself serves as a direct tracer of icesheet erosion of this source region, circumventing the uncertainties introduced by secondary calculations*", I disagree with this argument. I'm not disputing that that kaolinite and ARM are proxies for ice sheet erosion, but I do dispute that a record confounded by changes in sediment supply can be interpreted as changes in quantity X, when X is the concentration and not the absolute flux. Both the ARM and kaolinite records will be diluted the same amount, so they will of course covary.

The authors have then added (L255) "ARM shows correlations with deep ocean temperature (Tw), sea surface temperature change (ΔSST), benthic δ 256 18O, and sea level (Supplementary Fig. 13), corroborating its relationship with ice-sheet dynamics" I agree this is not inconsisent, but on the other hand it's not strongly supportive either. Very many environmental proxies are well correlated in the Southern Ocean (and globally) at these time scales. Even if the ARM record was not related to ice sheet changes (e.g., if related to circulation-driven changes in deposition rate) it would still very likely be correlated with SST,

deep water temp at d18O. Those three variables are of course themselves all very strongly correlated. Or, the correlation could arise because dilution by biogenic material is correlated with SST, sea ice conc, etc.

In summary: the records based on concentrations can be interpreted as a compelling story that supports other evidence for environmental change, but given the ambiguity introduced by dilution I don't think that conentration itself is strong enough for a Nature Comms article. Really you need to convince us that the *flux* of ASE-sourced sediments has increased at U1532.

(2) L237 "*The distribution and abundance of preserved grounded-ice wedges from the Pliocene to the Pleistocene indicate repeated ice-sheet advances to the shelf edge during the Pliocene, followed by limited further progression in the Pleistocene [Ref37]"*.

Can you build on the conclusions of Ref 37 Gohl et al 2021? I'm now wondering what can we *confidently* learn from the U1532 data, that was not evident from that finding in Gohl et al. 2021? Great that your results here support that earlier work, but are the grounding zone wedges combined with subsurface ocean T already more compelling than the U1532 record (given Comment 1 above)?

(3) Cross shelf transport

L184 "*Terrigenous sedimentation on the Antarctic continental rise is controlled by glacial processes that generate glaciogenic detritus on land and the continental shelf, which is then transported downslope via gravity flows—such as slumps and turbidity currents—through deep-sea channels to the continental slope and rise.* ***This process occurs primarily when the ice sheet advances onto the continental shelf during glacial periods****. Sedimentary facies analysis of Expedition 379 cores indicates that terrigenous debris was primarily delivered to the continental rise by glacial-advance gravity flows and deglacial hyperpycnal plumes generated while the grounding line remained near the shelf break and meltwater discharge intensified*"

Here I think is an important point to make, that under current conditions (maybe at least Pleistocene) the inland sloping shelf means that the downslope transport of sediments off the continental shelf **occurs only when the ice advances to the shelf edge**. See BedMachine plot below. Note that in Fig 1, an arrow showing "downslope" sediment transfer is pointing uphill away from the grounding line. It seems like a minor point but I think is an important part of the story. If as the authors suggest in their reply that "*Sediments may be routed through alternative, adjacent channels*" this opens up other ambiguities as to how the concentration signal at U1532 will likely be modified by changes in transport characteristics along a less direct pathway, presenting further difficulties with interpreting the record.

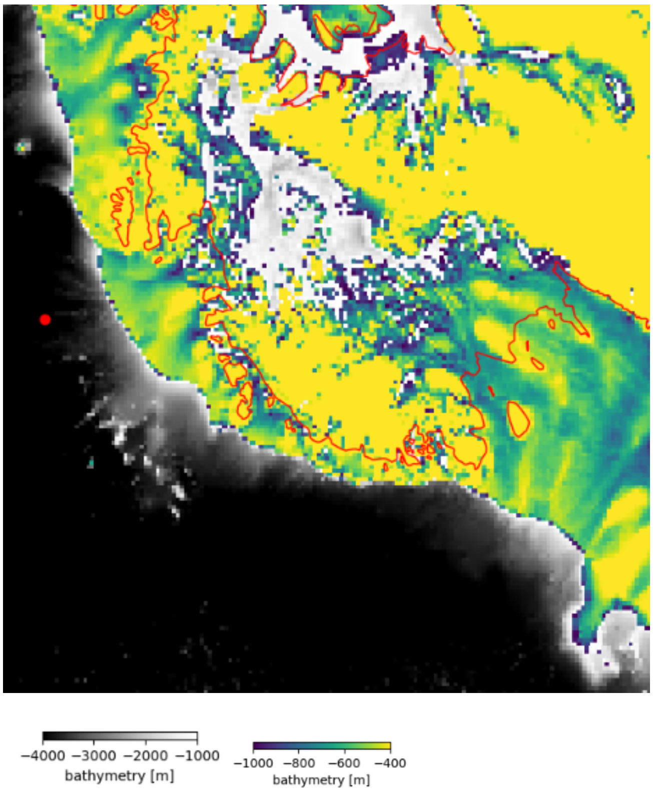

Fig plotted from BedMachine3 data, I've tried to adjust the colorscales to pick out the pertinent details. Red line is the Cartopy coast (approximates the groundling line quite well), red blob is U1532.

---

(4) Sources of kaolinite and magnetic minerals

The kaolinte and magnetic minerals are proposed to derive from on-shelf pre-Oligocene strata (L205 "*The content of clay mineral kaolinite is generally associated with chemical weathering of rock in warm and humid climates, and it has been suggested that pre-Oligocene sedimentary strata on the ASE shelf and its hinterland are the sources for the relatively high kaolinite contents found in the offshore sediments of the Amundsen Sea"* … then later L224 *"the magnetic minerals in the sediments at Site U1532 are mainly derived from the pre-Oligocene sedimentary strata on the ASE shelf."*)

I'm not disputing the provenance analysis, but the logic later is not entirely convincing.

The above statements seem sound on the basis that the pre-Oligocene strata were eroded by overlying ice, transported subglacially to the shelf break, and transported downslope to U1532 by gravity currents/slumping. However, the increase in kaolinte content and ARM since 3.2 Ma is not immediately consistent with the model proposed in Fig 5, where the Pliocene-Pleistocene ice sheet advance after 3.2 Ma is attributed to increasing stability provided by *increasing sediment thickness* on the shelf. That deposited sediment will reduce erosion of underlying rocks.

Are you suggesting the pre-Oligocene strata were eroded by the smaller Pliocene ice sheet, resulting sediments were trapped on the shelf, and were then reworked / transported subglacially to shelf edge during subsequent late-Pliocene advance? That explains the initial

increase in kaolinite, but given that Pleistocene sediments eventually cover the Pliocene sediments (top panel in Fig 5), where does the continued supply of kaolinite come from in the Pleistocene? Is it from further inland, then transported outwards episodicallly during glacial maxima?

This part of the interpretation and Fig 5 I think needs looking at carefully or rewording to make sure it's not contradictory (and make sure is consistent with the kaolinite & ARM *flux* records so we're not falsely building this interpretation on a record of dilution).

Minor points

L258 "*Despite typically higher sedimentation rates during glacial periods due to enhanced terrigenous input, a long-term decrease in rates accompanied ice advance after ~4.1 Ma (Fig. 2a). Decreased glacial erosion rates resulting from colder, drier subglacial conditions may contribute to the decreased sedimentation rates associated with cooling (Fig. 2a)"*

Very speculative and the cited reference (#49) alludes to the Antarctic Peninsula and Patagonia, not WAIS. If I wanted to speculate on ASE ice stream dynamics I would put my money on these being active, warm-based fast ice flow features throughout even the coldest glacial periods, with subglacial heating/melting sustained by frictional heating and localised strain heating as is the case today. If the ice streams shut down (as implied by colder subglacial conditions, which would switch off sliding) you would effictively shut down nearly all dynamic ice loss from this catchment.

L302 "*Atmospheric CO2 concentrations, ocean temperature and ocean currents are proposed to be the key factors influencing WAIS variability*"

CO2 influences WAIS stability through its influence on ocean temp & circulation. Could reword something like "*Atmospheric CO2-driven changes in ocean temp/circulation are proposed as key factors… etc*"
